# ESCRT-III assembles around mis-segregated DNA to protect genome stability

**DOI:** 10.1038/s41594-026-01841-4

**Published:** 2026-07-15

**Authors:** James Glover, Nathaniel Talledge, John McCullough, Akram Alian, Jessica B. A. Sadler, Sahil Kamboj, Nathaniel W. M. Dempsey, Thomas G. Laughlin, Henry C. Nguyen, Dawn M. Wenzel, Matthew S. Lalonde, Elliott L. Paine, Barbie Ganser-Pornillos, Leandro N. Ventimiglia, Dollie LaJoie, Janet H. Iwasa, Christopher P. Arthur, D. John Lee, Frank R. Moss, Meera Venkatesh, Lydia M. Arnold, Jarrod S. Johnson, Katharine S. Ullman, Adam Frost, Wesley I. Sundquist, Juan Martin-Serrano

**Affiliations:** 1 Department of Infectious Diseases, Faculty of Life Sciences & Medicine, King’s College London, London, UK; 2 Altos Labs, Bay Area Institute of Science, Redwood City, CA USA; 3 Department of Biochemistry, University of Utah School of Medicine, Salt Lake City, UT USA; 4 Chan Zuckerberg Biohub, San Francisco, CA USA; 5 Department of Biochemistry and Biophysics, University of California, San Francisco, CA USA; 6 Department of Oncological Sciences, Huntsman Cancer Institute, University of Utah, Salt Lake City, UT USA; 7 Division of Microbiology and Immunology, Department of Pathology, University of Utah School of Medicine, Salt Lake City, UT USA; 8 Quantitative Biosciences Institute, University of California, San Francisco, CA USA

**Keywords:** Checkpoints, Cryoelectron microscopy

## Abstract

Chromosome mis-segregation events that remain unresolved during cytokinesis threaten genome stability. Persistent ultrafine DNA bridges engage the Aurora B-dependent abscission checkpoint (termed NoCut), which delays abscission by phosphorylating components of the ESCRT complex. Here we show that NoCut surveillance repurposes human ESCRT-III, the membrane-remodeling complex that seals the reforming nuclear envelope in anaphase. In response to persistent ultrafine DNA bridges, ESCRT-III transfers from the reforming nuclear envelope to the mis-segregated DNA bridge and ESCRT-III complexes protect the DNA from damage, as evidenced by increased DNA damage upon CHMP1B depletion. Complementary in vitro assembly reactions show that the human ESCRT-III proteins CHMP1B and IST1 can copolymerize into double-stranded filaments that encase double-stranded DNA and nucleosomes and prevent nuclease digestion and cGAS recognition, demonstrating that ESCRT-III complexes can directly bind and protect DNA. Lastly, cells expressing a DNA-binding mutant of CHMP1B exhibit cytokinesis failure and binucleation when ultrafine DNA bridges persist, revealing a mechanism of safeguarding genome stability.

## Abstract (web summary)

The authors show that persistent ultrafine DNA bridges become coated by membranes and the ESCRT-III proteins CHMP1B and IST1, which protect the genome within from damage.

## Main

**Extended Data Fig. 1:**
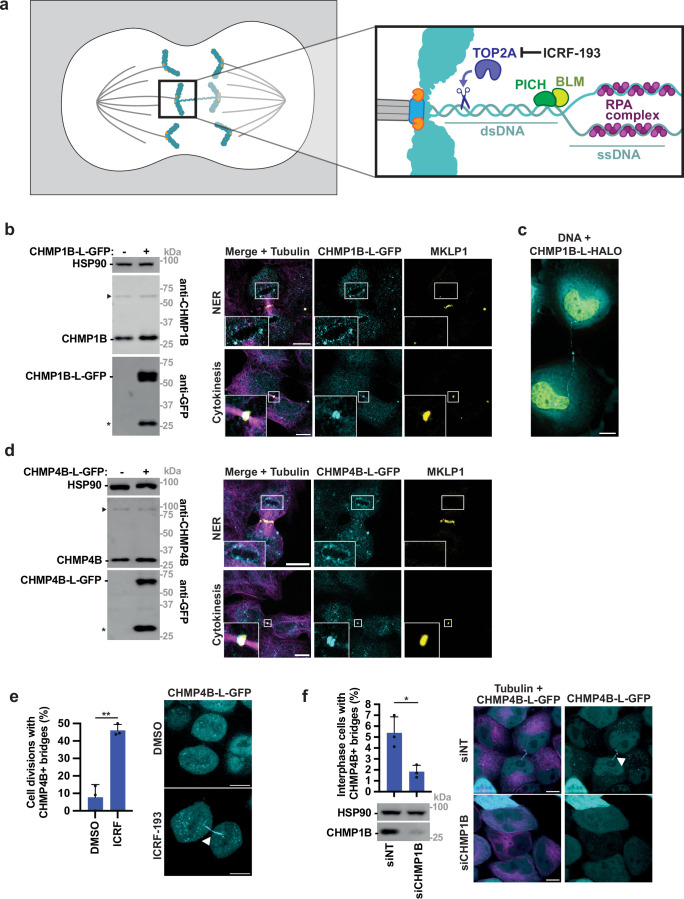
Validation of HeLa^CHMP1B-L-GFP^ and HeLa^CHMP4B-L-GFP^ cells and CHMP1B requirement to form CHMP4B bridges. **a**, Key factors that initially recognize and help resolve mis-segregating UFBs. Left panel: Dividing cell with a UFB present (teal double helix). Right panel: Zoomed in view of the UFB. The DNA translocase PICH (green) localizes to catenated dsDNA (teal double helix, underlined) and recruits the BLM helicase (yellow). As BLM mediates dsDNA conversion to ssDNA (teal single helices, underlined), heterotrimeric RPA complex (magenta), composed of RPA1-3 subunits (also known as RPA70, RPA32 and RPA14), is recruited. TOP2A (blue) mediates catenated DNA resolution. ICRF-193 poisons TOP2A, increasing the proportion of cell divisions with UFBs. **b**, Validation of HeLa^CHMP1B-L-GFP^ cells. Left panel: Western blots of HeLa and HeLa^CHMP1B-L-GFP^ cells (anti-HSP90, anti-CHMP1B and anti-GFP). Note that CHMP1B-L-GFP is not visible on the anti-CHMP1B blot. Arrowhead indicates a non-specific band near the expected size of exogenous CHMP1B-L-GFP and asterisk denotes an apparent GFP cleavage band. Right panels: Fixed cells stained with anti-GFP (cyan), anti-MKLP1 (yellow) and anti-tubulin (magenta) antibodies. Localization of CHMP1B-L-GFP to the NE during NER (top-middle panel) and midbody during cytokinesis (bottom-middle panel). **c**, CHMP1B-L-HaloTag localizes to UFBs. HeLa^CHMP1B-L-HaloTag^ cells treated with 100 nM ICRF-193 for 18 h, then visualized with JF635 HaloTag ligand (cyan) and Hoechst-33342 (yellow) by live-cell widefield fluorescence microscopy. **d**, Validation of HeLa^CHMP4B-L-GFP^ cells. Left panel: Western blots of HeLa and HeLa^CHMP4B-L-GFP^ cells (anti-HSP90, anti-CHMP4B and anti-GFP). Note that CHMP1B-L-GFP is not visible on the anti-CHMP1B blot. Arrows indicate a non-specific band near the expected exogenous CHMP4B-L-GFP size. Arrowhead and asterisk denote the same features as in b. Right panels: Cells fixed and stained with anti-GFP (cyan), anti-MKLP1 (yellow) and anti-tubulin (magenta) antibodies. Localization of CHMP4B-L-GFP to the NE during NER (top-middle panel) and midbody during cytokinesis (bottom-middle panel). **e**, ICRF-193 induces CHMP4B positive bridges during mitosis. HeLa^CHMP4B-L-GFP^ cells treated with DMSO (n = 37 cells) or 80 nM ICRF-193 (n = 35 cells), analyzed by live-cell microscopy, and quantified for CHMP4B-L-GFP (cyan)-positive bridges that formed during cell division. White arrowhead indicates a bridge. **f**, Depletion of CHMP1B reduces the formation of CHMP4B positive bridges during interphase. HeLa^CHMP4B-L-GFP^ cells treated with 80 nM ICRF-193 (siNT n = 437 cells; siCHMP1B n = 331 cells) for 24 h and incubated with SiR-Tubulin (magenta) and quantified for CHMP4B-L-GFP (cyan)-positive bridges that formed during cell division. Western blot shows CHMP1B silencing. Right panels: White arrowhead indicates a bridge. Representative images in **b-f** are from 3 independent experiments. Scale bars in **b-f** are 10 µm. Statistical analysis in **e,f**, unpaired two-tailed t-test. Error bars in **e,f** show mean ± SD, independent experiments plotted as dots. *P* > 0.05 was not significant (ns); *P* ≤ 0.05 (*), *P* ≤ 0.01(**), *P* ≤ 0.001(***), *P* ≤ 0.0001 (****) were considered significant. Source data

DNA mis-segregation threatens genome stability, as failure to resolve these mitotic conflicts can result in severe genomic rearrangements^1^. Several mechanisms exquisitely sense and resolve mis-segregation events, including the spindle assembly checkpoint (SAC), which arrests mitosis in response to aberrantly attached kinetochores^2^. However, persistent interchromatid entanglements known as ultrafine DNA bridges (UFBs) are not sensed by the SAC as these bridges do not interfere with microtubule–kinetochore attachments^3,4^. Instead, UFB resolution is initiated by PICH, a DNA translocase and the heterotrimeric RPA complex composed of the RPA1, RPA2 and RPA3 subunits (also known as RPA70, RPA32 and RPA14, respectively), which binds to single-stranded DNA (ssDNA)^5,6^. UFB resolution can then be completed by topoisomerase IIa and the BLM helicase^7^ (Extended Data Fig. 1a). Although these protective mechanisms are efficient, UFBs are not always resolved and can instead persist after reformation of the nuclear envelope (NE) and extend between daughter nuclei^8,9^.

Persistent UFBs and chromatin bridges pose multiple threats to the genome. First, these bridges can cause tetraploidization by inducing cytokinesis failure^10–12^. Moreover, bridges that persist beyond cytokinesis can be broken or can damage the NE, initiating iterative cycles of genome instability that rapidly generate hallmark features of cancer cells such as micronucleation, chromothripsis and karyotype alterations^9,13–15^. Such alterations are also strongly associated with cell divisions that exhibit DNA bridges in individual-derived cancer organoids^16^. In many individual cases, however, naturally occurring DNA bridges do not produce genome instability^16^, indicating that protective mechanisms resolve a subset of these conflicts with high fidelity. Crucially, UFBs and other mis-segregation events that persist beyond anaphase engage the NoCut checkpoint^8,11,17–19^. Sustained NoCut signaling delays cytokinetic abscission (the final step in cell division), prevents binucleation and minimizes downstream DNA damage associated with mis-segregation^20^.

The ESCRT (endosomal sorting complex required for transport) pathway is ideally placed to manage persistent DNA bridges because it drives both closure of the NE at the end of anaphase and orchestrates cytokinetic abscission^21–23^. In the former case, the inner nuclear membrane (INM) protein LEM2 recruits ESCRT-III membrane-remodeling complexes to seal remaining holes in the reforming NE^24^. LEM2 activates the ESCRT factor CHMP7, which in turn nucleates formation of ESCRT-III filaments that seal the NE fenestrations^25–27^. The spatiotemporal distribution of the CHMP7–ESCRT-III axis is determined by CC2D1B, a regulator of ESCRT-III polymerization that ensures orderly NE reformation (NER)^28^. During cytokinesis, the ESCRT machinery then mediates abscission through a similar but CHMP7-independent cascade of events within the midbody^29,30^. Early-acting ESCRT factors are first recruited to the midbody by CEP55, where they facilitate the assembly of large ESCRT-III filaments that mediate membrane constriction^30–35^. Irreversible separation of the daughter cells also requires microtubule severing and F-actin removal at the abscission site^36–38^.

Tight integration between ESCRTs and NoCut preserves genome stability^19,39–42^. A unique ESCRT-III subunit, CHMP4C, coordinates NoCut with abscission delay. CHMP4C phosphorylation by Aurora B initiates a signaling cascade that delays abscission^19,43^ and sustaining this inhibitory signal beyond cytokinesis requires the downstream phosphorylation of ESCRT-III proteins by ULK3 (ref. ^44^). Importantly, we showed that CHMP4C^T232^, an allele that predisposes to ovarian cancer^45^, disrupts NoCut and increases genome instability under conditions that increase the burden of chromosome mis-segregation, leading to elevated DNA damage and aneuploidy^14^.

Here, we reveal that NoCut signaling redirects ESCRT-III subunits from the reforming NE onto mis-segregated DNA. We find that ESCRT-III subunits, including CHMP1B and IST1, coassemble with unresolved UFBs inside an LEM2-rich membrane sleeve, thereby creating a protective coating around the mis-segregating DNA in the NoCut-arrested state. ESCRT-III coat formation requires CHMP7, indicating that it initiates at the reforming NE. Coat formation is also integrated with NoCut signaling because efficient coat formation requires key NoCut regulatory factors, including CHMP4C and ULK3 kinase. Conversely, ESCRT-III coats also contribute to NoCut maintenance because CHMP1B is selectively required to trigger NoCut responses to mis-segregated DNA. Loss of CHMP1B or weakening of its DNA-binding activity leads to increased DNA damage, cytokinetic bridge regression and tetraploidization. Thus, coating of UFBs by CHMP1B represents a key NoCut mechanism for genome stability protection.

## Results

### ESCRT-III proteins associate with DNA bridges

Humans express 12 distinct but homologous ESCRT-III subunits that are classified into 8 related families termed CHMP1–CHMP7 and IST1. ESCRT-III subunits form membrane-remodeling filaments that constrict membranes and coordinate other ESCRT-associated biochemical activities, such as cargo deubiquitination during intralumenal endosomal vesicle formation and microtubule severing during cell division and NE closure^37,46–48^. To study ESCRT-III behavior and function, we fused enhanced green fluorescent protein (eGFP) to the ESCRT-III subunit CHMP1B through a 25-nm flexible linker, which preserves ESCRT-III function^28^. The resulting CHMP1B-L–GFP construct was undetectable by anti-CHMP1B antibodies, indicating stable expression at subphysiological levels in HeLa cells (Extended Data Fig. 1b). This construct accurately localized transiently to the reforming NE and then to the midbody, as analyzed by spinning-disk microscopy (Extended Data Fig. 1b).

**Fig. 1:**
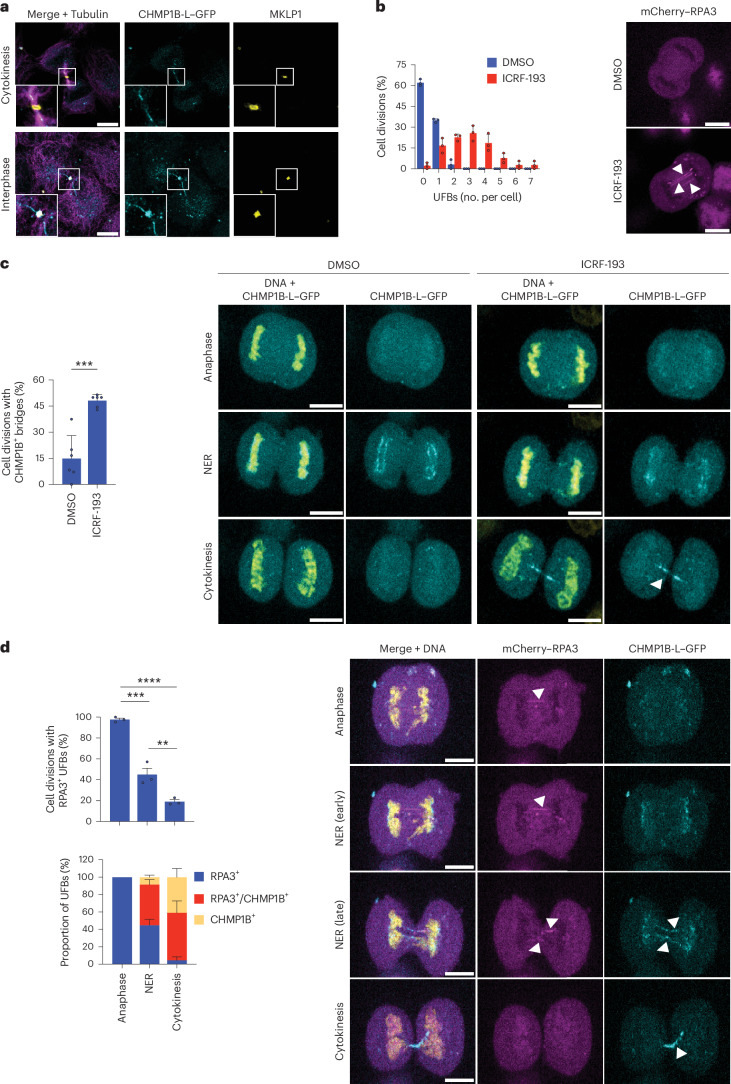
ESCRT-III bridges derive from unresolved UFBs and persist into interphase. **a**, CHMP1B-L–GFP forms bridges during mitosis that persist into interphase. HeLa^CHMP1B-L–GFP^ cells were fixed and stained with anti-GFP (cyan), anti-MKLP1 (yellow) and anti-tubulin (magenta) antibodies. Representative images are shown (*n* = 3 independent experiments). **b**, ICRF-193 induces RPA3-positive UFBs during mitosis. HeLa^mCh–RPA3^ cells were treated with DMSO (*n* = 102 cells) or 80 nM ICRF-193 (*n* = 122 cells) for 1 h and analyzed by live-cell microscopy. Quantification of the number of distinct mCh–RPA3-positive (magenta) UFBs that formed during each cell division (*n* = 3 independent experiments). White arrowheads indicate mCh–RPA3 bridges. **c**, ICRF-193 induces CHMP1B-positive bridges during mitosis. HeLa^CHMP1B-L–GFP^ cells were treated with DMSO (*n* = 75 cells) or 80 nM ICRF-193 (*n* = 94 cells), labeled with Hoechst 33342 (DNA; yellow) and analyzed by live-cell microscopy. Quantification of CHMP1B-L–GFP-positive bridges (cyan) that formed during cell division (*n* = 6 independent experiments). CHMP1B-L–GFP localization to the NE was used to determine NER. Anaphase was defined as the period between mitotic onset and NER. Cytokinesis was defined as the point after NER and before the completion of cell division. White arrowheads indicate bridges. **d**, CHMP1B bridges are derived from unresolved RPA3-positive UFBs. HeLa^CHMP1B-L–GFP/mCh–RPA3^ cells were treated with 80 nM ICRF-193 (*n* = 122 cells), labeled with Hoechst 33342 (DNA; yellow) and analyzed by live-cell microscopy. Top, quantification of mCh–RPA3-positive UFBs that formed and/or remained present during anaphase, NER or cytokinesis (*n* = 3 independent experiments). CHMP1B-L–GFP localization to the NE was used to determine the onset and completion of NER. Anaphase was defined as the time before NER, while cytokinesis was defined as the time after NER completion. Bottom, quantification of bridges that were positive for mCh–RPA3 (magenta), CHMP1B-L–GFP (cyan) or both during anaphase, NER and cytokinesis (*n* = 3 independent experiments). White arrowheads indicate bridges. Scale bars, 10 µm (**a**–**d**). Statistical analysis was conducted using an unpaired two-tailed *t*-test (**c**) or Tukey’s multiple-comparison test (**d**). Error bars in **b**–**d** represent the mean ± s.d.; independent experiments are plotted as dots. *P ≤ 0.05, **P ≤ 0.01, ***P ≤ 0.001 and ****P ≤ 0.0001. Source data

Notably, in a subset of cells, CHMP1B-L–GFP also accumulated at exceptionally long tubular structures visible at the midbody stage of cytokinesis and throughout interphase (Fig. 1a). These elongated structures stretched between the daughter nuclei and resembled previously reported interphase intercellular bridges containing CHMP4B (ref. ^44^) and LEM2-rich nuclear membrane^49^. Given that CHMP4B-positive intercellular bridges have been associated with DNA bridges^50^, we reasoned that mis-segregated DNA might also induce formation of CHMP1B-L–GFP-containing bridges at the end of anaphase. To test this idea, we used low doses of the topoisomerase II inhibitor, ICRF-193, to increase UFB formation by inhibiting the cell’s capacity to resolve DNA entanglements^8^ while avoiding abortive anaphases^51^. We generated HeLa cells stably expressing mCherry (mCh)–RPA3, an RPA subunit that prevents nonspecific UFB breakage^6^. Low-dose ICRF-193 treatment increased the frequency of cell divisions exhibiting mCh–RPA3-positive UFBs to nearly 100%, with an average of ~3 RPA bridges per cell, as determined by live-cell microscopy (Fig. 1b). These bridges were primarily visible during anaphase but a subset persisted into cytokinesis (Supplementary Video 1). Similar analysis of CHMP1B-L–GFP showed that ~15% of dividing cells exhibited CHMP1B-L–GFP at tubular structures that formed during mitotic NER (Fig. 1c). Importantly, ICRF-193 treatment tripled their frequency to 45% (Fig. 1c). Lastly, a second fluorescently labeled CHMP1B fusion protein (CHMP1B-L–HaloTag) similarly localized to internuclear bridges, thereby validating this CHMP1B localization pattern with two different fluorescent markers (Extended Data Fig. 1c). We then asked whether other ESCRT-III subunits are also incorporated into intercellular bridges following ICRF-193 treatment. We focused on CHMP4B, an abundant and essential ESCRT-III subunit required for midbody severing and NE reassembly^25,26,30,31^. CHMP4B-L–GFP was stably expressed at subphysiological levels and localized both to the reforming NE and to the midbody during cytokinesis, as expected (Extended Data Fig. 1d). Analysis by live-cell confocal microscopy showed internuclear bridges containing CHMP4B-L–GFP in 8% of cell divisions, which increased more than five-fold to 45% upon ICRF-193 treatment (Extended Data Fig. 1e). In agreement with previous observations, CHMP4B-L–GFP intercellular bridges were also observed in interphase^50^ and, notably, their frequency was reduced upon short interfering RNA (siRNA) depletion of CHMP1B compared to nontargeting siRNA control (Extended Data Fig. 1f). Thus, persistent DNA entanglements induce the formation of mitotic ESCRT-III bridges that persist beyond cytokinesis and CHMP1B facilitates the incorporation of additional ESCRT-III subunits into these bridges.

### Temporal maturation of ESCRT bridges

Despite their common induction by ICRF-193, CHMP1B-positive bridges were less prevalent than RPA3 bridges and the two types of bridges appeared to be enriched at different stages of mitotic exit. To establish the spatiotemporal relationship between these markers, we stably coexpressed mCh–RPA3 and CHMP1B-L–GFP in HeLa cells. Analysis by live-cell microscopy following ICRF-193 treatment showed that, as expected^52^, the overall frequency of daughter cells with mCh–RPA3-decorated bridges decreased as cell division progressed from anaphase to NER to cytokinesis (Fig. 1d).

**Extended Data Fig. 2:**
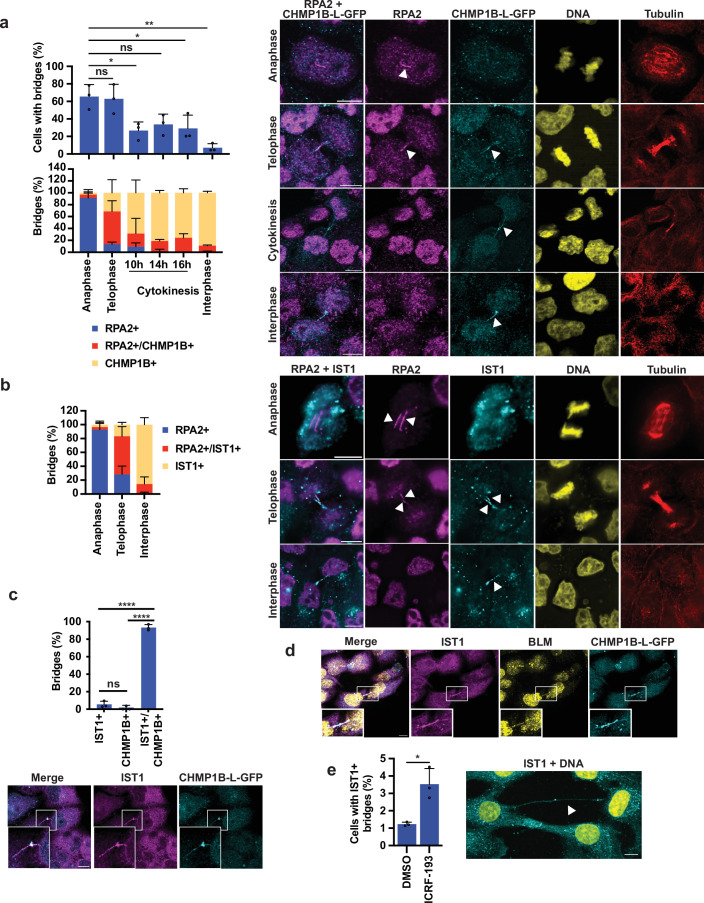
ESCRT-III proteins CHMP1B and IST1 co-localize with the UFB markers RPA2 and BLM in bridges. **a**, CHMP1B progressively replaces RPA2 on UFBs that persist into interphase. HeLa^CHMP1B-L-GFP^ cells were synchronized with a single thymidine block (2 mM, 24 h) and treated with 160 nM ICRF-193. Top left panel: Quantification of the proportion of cells with bridges positive for either CHMP1B-L-GFP or RPA2 at anaphase (n = 69 cells), telophase (n = 141 cells), cytokinesis-10h (n = 130 cells) cytokinesis-14h (n = 124 cells), cytokinesis-16h (n = 136 cells), and interphase (n = 1096 cells). Bottom left panel: Quantification of the proportion of UFBs that were positive for either RPA2, CHMP1B-L-GFP or both at anaphase (n = 81), telophase (n = 157), cytokinesis-10h (n = 65), cytokinesis-14h (n = 67), cytokinesis-16h (n = 62) and interphase (n = 106). Right panels: Representative cells fixed and stained with anti-tubulin (red), anti-RPA2 (magenta), anti-GFP (cyan) antibodies and DAPI (yellow). **b**, IST1 progressively replaces RPA2 in UFBs that persist into interphase. HeLa cells were treated with 160 nM ICRF-193, fixed, and stained with anti-tubulin (red), anti-RPA2 (magenta), anti-IST1 (cyan) antibodies and DAPI (yellow). Quantification shows the proportion of UFB structures that were positive for RPA2, IST1 or both at anaphase (n = 122 bridges), telophase (n = 139 bridges) and interphase (n = 130 bridges) and images show representative cells. **c**, IST1 coats CHMP1B bridges. HeLa^CHMP1B-L-GFP^ cells were treated with 100 nM ICRF-193 for 18 h, fixed and stained with anti-IST1 (magenta) and anti-GFP (cyan) antibodies. Quantification reports bridges (n = 103) that were positive for either IST1, CHMP1B-L-GFP or both and images show representative cells. **d**, BLM coats CHMP1B/IST1 bridges. Representative image of HeLa^CHMP1B-L-GFP^ cells treated with 80 nM ICRF-193 for 24 h, fixed and stained with anti-IST1 (magenta), anti-BLM (yellow) and anti-GFP (cyan) antibodies. **e**, Endogenous IST1 localizes to UFBs in RPE cells. RPE cells treated with DMSO (n = 559 cells) or 80 nM ICRF-193 (n = 584 cells) for 24 h, fixed and stained with anti-IST1 (cyan) antibody and SPY555-DNA (yellow). Quantification reports the fraction of cells with IST1+ bridges and image shows a IST1+ bridge (arrowhead). Representative images in **a-e** are from 3 independent experiments. Scale bars in **a-e** are 10 µm. Statistical analysis performed in **a-c**, Tukey’s multiple comparisons test, **e**, unpaired two-tailed t-test. Error bars in **a-c,e**, represent mean ± SD, independent experiments are plotted as dots. *P* > 0.05 was considered not significant (ns), while *P* ≤ 0.05 (*), *P* ≤ 0.01(**), *P* ≤ 0.001(***), *P* ≤ 0.0001 (****) were considered significant. Source data

Unresolved UFBs switched from exclusive labeling by mCh–RPA3 in anaphase to dual labeling by both mCh–RPA3 and CHMP1B-L–GFP during NER. Then, as cells progressed into cytokinesis, the UFBs remained stably labeled with CHMP1B-L–GFP, with a subset remaining dually labeled with both mCh–RPA3 and CHMP1B-L–GFP (Fig. 1d and Supplementary Video 1). To characterize these observations further, we synchronized HeLa^CHMP1B-L–GFP^ cells by single thymidine block and performed immunofluorescence upon release using antibodies against endogenous RPA2, an RPA subunit. In agreement with the live-cell analysis, the proportion of bridge-connected cells diminished as cell division progressed (Extended Data Fig. 2a). This experimental approach confirmed that exclusively coated RPA2 bridges transitioned into dual RPA2–CHMP1B coating during telophase and became largely positive for CHMP1B as cells completed cytokinesis and progressed into interphase.

We also imaged IST1, an ESCRT-III subunit that preferentially copolymerizes with CHMP1B (ref. ^53^). In agreement with the CHMP1B data, endogenous IST1 colocalized with RPA2-positive UFBs in telophase and these UFBs transitioned to IST1-only bridges in interphase (Extended Data Fig. 2b). Lastly, endogenous IST1 strongly localized with CHMP1B-L–GFP in interphase bridges that were induced by ICRF-193 (Extended Data Fig. 2c) and both ESCRT-III subunits colocalized with the UFB component BLM in intercellular bridges (Extended Data Fig. 2d), thus supporting the presence of a CHMP1B–IST1 coassembly within persistent UFBs. These observations indicate that unresolved UFBs evolve into ESCRT-III-containing bridges during NER, and that these structures can persist into the subsequent interphase.

**Fig. 2:**
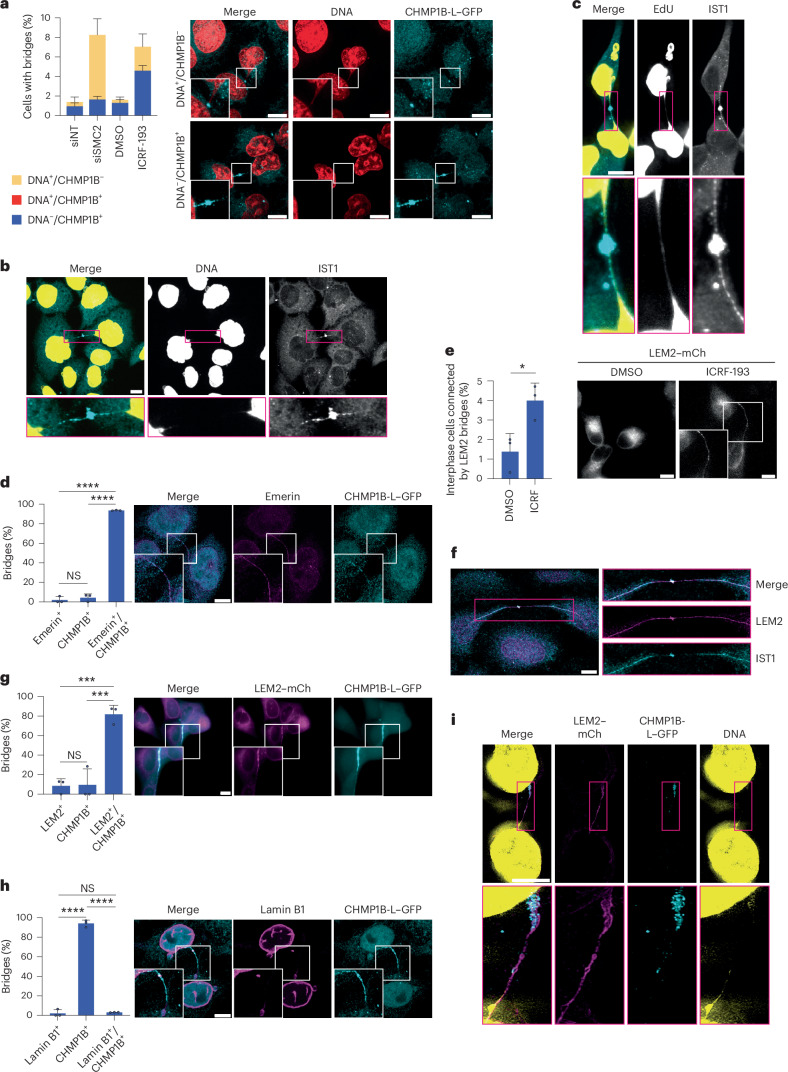
ESCRT-III associates with DNA bridges coated in NE proteins. **a**, Low-dose ICRF-193 specifically induces DNA^−^ CHMP1B^+^ bridges. HeLa^CHMP1B-L–GFP^ cells were transfected with non-targeting siRNA control (siNT; *n* = 934 cells) or SMC2 siRNA (siSMC2; *n* = 962 cells) or treated with DMSO (*n* = 929 cells) or 80 nM ICRF-193 (*n* = 935 cells), then fixed and stained with anti-GFP (cyan) and SPY555-DNA (red). Quantification of the proportion of cells with bridges that were positive for DNA, CHMP1B or both is shown. **b**, Interphase IST1 bridges originate at the boundary of nuclei. HeLa cells were treated with 80 nM ICRF-193 for 24 h, fixed and stained with SPY555-DNA (yellow) and anti-IST1 (cyan) antibody. **c**, Interphase IST1 bridges colocalize with DNA. HeLa cells were treated with 80 nM ICRF-193 and 10 µM EdU for 24 h, fixed and stained with Click-iT EdU 555 (yellow) and anti-IST1 antibody (cyan). Images are maximum intensity projections combining seven *z* stacks of 0.3-µm steps. **d**, Emerin coats CHMP1B bridges. HeLa^CHMP1B-L–GFP^ cells were treated with 100 nM ICRF-193 for 18 h, fixed and stained with anti-emerin (magenta) and anti-GFP (cyan) antibodies. Quantification of bridges (*n* = 94) that were positive for emerin, CHMP1B-L–GFP or both. **e**, Interphase LEM2 bridges are induced by ICRF-193. Quantification of LEM2–mCh (gray) bridges connecting interphase HeLa^LEM2–mCh^ cells following treatment with 100 nM ICRF-193 (*n* = 1,213 cells) or DMSO (*n* = 1,325 cells) for 18 h. **f**, LEM2 coats IST1 bridges. HeLa cells were fixed and stained with anti-LEM2 (magenta) and anti-IST1 (cyan) antibodies. **g**, LEM2 coats CHMP1B bridges. HeLa^CHMP1B-L–GFP/LEM2–mCh^ cells were treated with 100 nM ICRF-193 for 18 h. Bridges (*n* = 53) were quantified as positive for LEM2–mCh (magenta), CHMP1B-L–GFP (cyan) or both. **h**, Lamin B1 is not present in CHMP1B bridges. HeLa^CHMP1B-L–GFP^ cells were treated with 100 nM ICRF-193 for 18 h, fixed and stained with anti-lamin B1 (magenta) and anti-GFP (cyan) antibodies. Bridges (*n* = 94) were quantified as positive for lamin B1, CHMP1B-L–GFP or both. **i**, CHMP1B assemblies are located within LEM2-positive membranes that surround UFBs. Representative structured illumination reconstruction of HeLa^LEM2–mCh/CHMP1B-L–GFP^. Cells were treated with 100 nM ICRF-193 for 18 h and stained with anti-GFP and anti-mCh antibodies. DNA (yellow), LEM2–mCh (magenta) and CHMP1B-L–GFP (cyan) are shown. Scale bars, 10 µm (**a**–**i**). Representative images in **a**–**i** are from three independent experiments. Statistical analysis was conducted using a Tukey’s multiple-comparison test (**a**,**d**,**g**,**h**) or unpaired two-tailed *t*-test (**e**). Error bars in **a**,**d**,**e**,**g**,**h** represent the mean ± s.d.; independent experiments are plotted as dots. *P ≤ 0.05, **P ≤ 0.01, ***P ≤ 0.001 and ****P ≤ 0.0001. NS, not significant. Source data

We then asked whether CHMP1B-positive bridges formed in response to mis-segregated chromatin and/or to UFBs. We performed siRNA against SMC2, as depletion of this condensin subunit induces formation of massive chromatin bridges by perturbing the compact architecture of chromosomes^54^. As expected, depletion of SMC2 increased the frequency of chromatin bridges that were stained with SPY555-DNA (Fig. 2a) but these bridges lacked CHMP1B-L–GFP. By contrast, ICRF-193 treatment increased the frequency of CHMP1B-L–GFP bridges and these bridges were predominantly negative for an SPY555-DNA signal, as would be expected for UFBs because of their low DNA content and single-stranded nature^52^.

We next investigated whether the ESCRT-III-positive bridges contained mis-segregated DNA. Immunofluorescence analysis of endogenous IST1 showed that interphase ESCRT-III bridges emanated from NE extensions formed at the boundaries between the two connected nuclei in HeLa (Fig. 2b) and retinal pigment epithelium (RPE-1) cells (Extended Data Fig. 2e). These bridges often connected nuclei under tension, as evidenced by protrusion of the chromatin mass at the points of contact with the IST1 bridge (Fig. 2b). This tension was consistent with the presence of genomic DNA tethers within the bridges but DNA in the internuclear bridges beyond the NE-proximal extension sites was undetectable using DNA-intercalating probes, presumably because conventional DNA dyes cannot detect naked DNA in UFBs^52^.

To visualize the DNA contents of ESCRT-III bridges, we took advantage of a Click-iT compatible EdU incorporated at the time of DNA synthesis and then fluorescently labeled^55^, together with immunofluorescence detection of endogenous IST1 (Fig. 2c). This procedure enabled the visualization of DNA within the ESCRT-III-positive intercellular bridges that connected daughter nuclei. Collectively, these results support the idea that ESCRT-III selectively associates with unresolved UFBs but not with chromatin bridges.

### Organization of cellular DNA–ESCRT-III complexes

The NE surrounds mis-segregated chromatin from late anaphase onward^11^. Therefore, we reasoned that ESCRT-III bridges might also be coated by an extended NE membrane. Cells stably expressing CHMP1B-L–GFP were fixed and labeled with antibodies against another LEM domain-containing integral INM protein, emerin. This analysis showed that nearly all interphase CHMP1B-L–GFP bridges colocalized with endogenous emerin (Fig. 2d). Similarly, the INM marker LEM2–mCh also labeled internuclear bridges that were induced by low-dose ICRF-193 treatment (Fig. 2e). Immunofluorescence analysis showed colocalization of endogenous IST1 and LEM2 at internuclear bridges (Fig. 2f). Stably coexpressed CHMP1B-L–GFP and LEM2–mCh similarly colocalized to ICRF-193-induced CHMP1B-L–GFP bridges (Fig. 2g). Thus, ESCRT-III bridges that transition from unresolved UFBs are coated with a sleeve of NE membrane. The NE in chromatin bridges is often depleted of structural proteins such as lamins^15^, compromising stability, and this was also true of the UFBs because the lamina component lamin B1 did not colocalize with CHMP1B-L–GFP interphase bridges (Fig. 2h).

We then studied the radial organization of ESCRT-III bridges. Given that extended ESCRT-III bridges may have diameters below the diffraction limit, we took advantage of the larger diameter of CHMP1B-L–GFP bridges near their junctions with nuclei. This strategy was combined with structured illumination microscopy (SIM), a super-resolution approach that revealed the localization of membrane-associated LEM2–mCh on the outermost layer of the bridge complex. By contrast, CHMP1B-L–GFP localized largely within the limiting membrane (Fig. 2i). Thus, CHMP1B resides within a NE sleeve that contains transmembrane LEM domain proteins, including emerin and LEM2.

### ESCRT-III proteins associate with cytoplasmic DNA

**Fig. 3:**
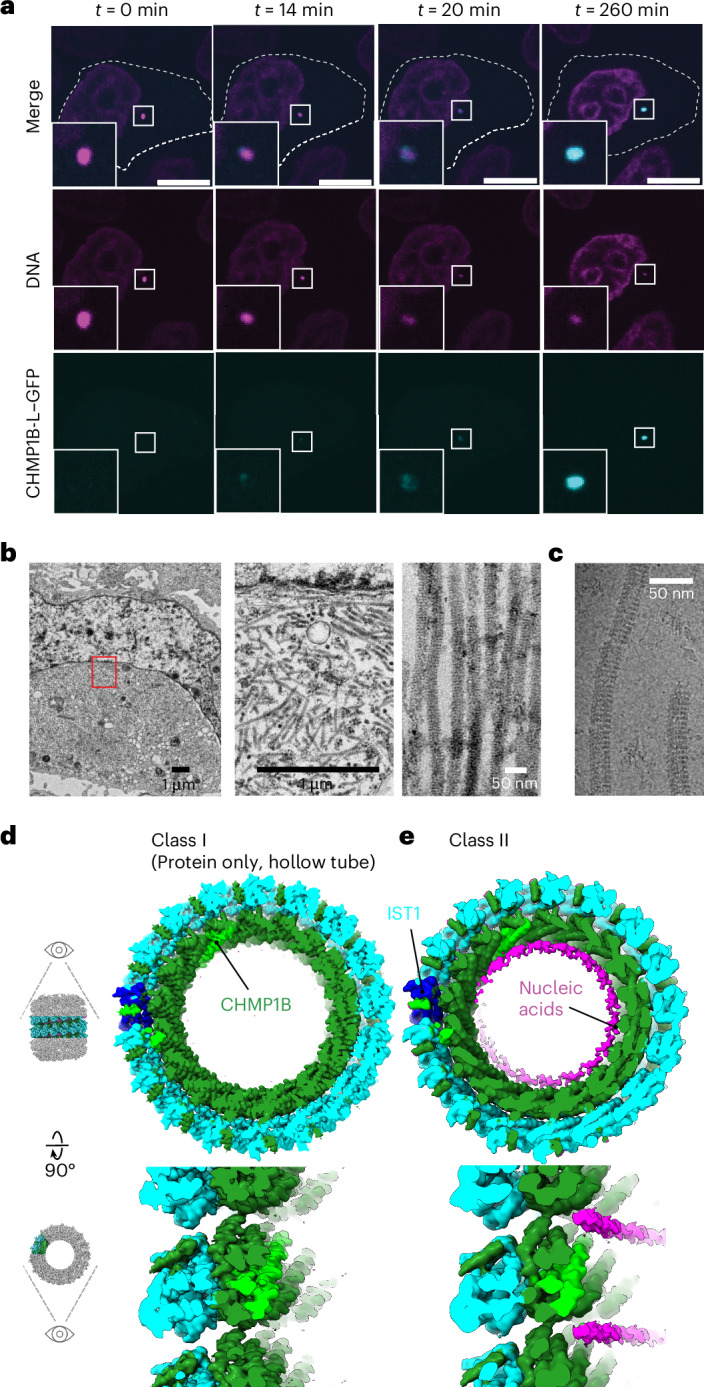
CHMP1B and IST1 engage DNA in cells. **a**, CHMP1B encapsulates transfected DNA. HeLa^CHMP1B-L–GFP^ cells were transfected with pCR3.1 plasmid, stained with SPY555-DNA and analyzed by live-cell imaging 4 h after transfection. Frames were taken every 2 min across five *z* stacks with 1-µm steps. Each image represents a single *z* plane. CHMP1B-L–GFP (cyan) and SPY555-DNA (magenta) are shown with the cell boundary outlined (dashed white line). Time is indicated. Scale bars, 10 µm. **b**, Transmission electron microscopy (TEM) images of plastic-embedded, thin-sectioned HeLa cells overexpressing CHMP1B and IST1. Scale bars indicate increasing magnifications. **c**, Cryo-EM image of CHMP1B–IST1 copolymers isolated from extracts of HeLa cells overexpressing CHMP1B and IST1. **d**,**e**, Cryo-EM reconstructions of extracted CHMP1B–IST1 copolymers. Cryo-EM reconstructions of two CHMP1B–IST1 tube classes isolated from HeLa cells. Class I comprises only protein (33% of particles) (**d**), whereas class II has bound nucleic acid (4.7% of particles) (**e**). IST1 forms the outer strand (cyan; dark blue highlights one monomer) in both reconstructions. CHMP1B forms the inner strand (green; light green highlights one monomer). Nucleic acids density (magenta) between rungs of the class II helical assembly.

**Extended Data Fig. 3:**
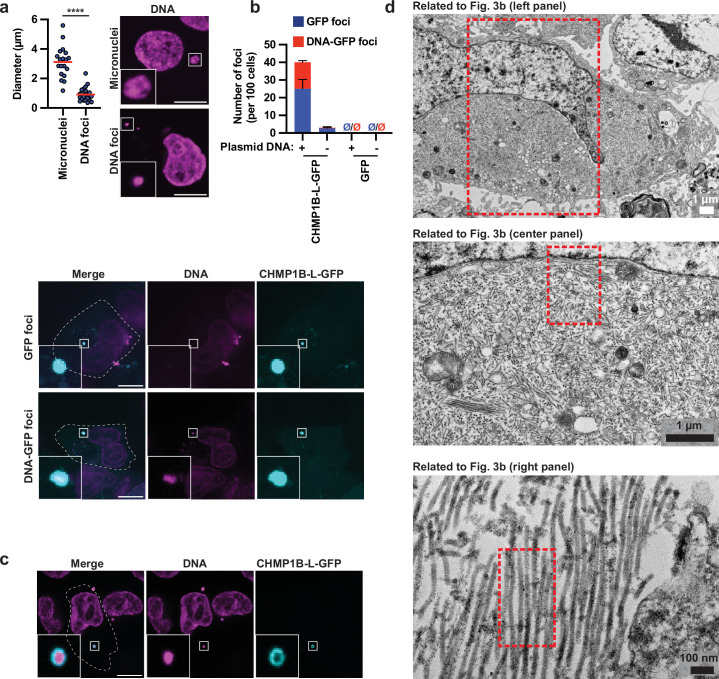
Transfected DNA induces formation of CHMP1B/DNA complexes. **a**, Transfected DNA foci are distinct from micronuclei. HeLa^CHMP1B-L-GFP^ cells were transfected with or without pCR3.1 DNA and labelled with SPY555-DNA. Live cells were imaged 4 h post-transfection. Images were taken and quantified at the point where the longest diameter of DNA could be observed. The diameters of micronuclei (n = 19 cells) and DNA foci (n = 22 cells) were measured in ImageJ, shown with mean (red line). Micronuclei were scored in cells transfected without plasmid. **b**, Transfected DNA induces the formation of CHMP1B foci. HeLa^CHMP1B-L-GFP^ or HeLa^GFP^ cells were transfected with or without pCR3.1 DNA and labeled with SPY555-DNA. Quantification shows the number of GFP and DNA-GFP foci per 100 cells, from 3 independent experiments. Slashed zeroes indicate values of zero in the graph. Images show max intensity projections of 0.3 µm x 3 Z-stacks. **c**, CHMP1B foci are filled with DNA. Low exposure was used to illuminate the DNA-CHMP1B foci. A representative, single Z-plane confocal image of HeLa^CHMP1B-L-GFP^ cells incubated with SPY555-DNA (magenta), with CHMP1B-L-GFP (cyan). A white dotted line outlines the cell boundary. **d**, TEM images of plastic-embedded, thin sectioned HeLa cells overexpressing CHMP1B and IST1. Red dashed boxes indicate regions used for the images shown in Fig. 3b. Scale bars are indicated. Representative images in **a-d** are from 3 independent experiments. Scale bars in **a-c** are 10 µm. Statistical analysis performed in **a**, unpaired two-tailed t-test, **b**, Tukey’s multiple comparisons test. Error bars in **b** represent mean ± SD, independent experiments are plotted as dots. *P* > 0.05 was considered not significant (ns), while *P* ≤ 0.05 (*), *P* ≤ 0.01(**), *P* ≤ 0.001(***), *P* ≤ 0.0001 (****) were considered significant. Source data

The experimental evidence above suggested that CHMP1B and perhaps other ESCRT-III proteins might associate directly with DNA. To test this idea in cells, we introduced ectopic DNA in the cytoplasm by transfecting plasmid DNA into HeLa cells that stably expressed subphysiological levels of CHMP1B-L–GFP. The transfected cells exhibited small cytoplasmic DNA foci (~1 µm in diameter) that were typically significantly smaller than conventional micronuclei (~3 µm), as visualized by SPY555-DNA dye staining (Extended Data Fig. 3a). Over a period of several hours, these cytoplasmic DNA foci gradually became coated with CHMP1B-L–GFP (Fig. 3a and Supplementary Video 2). It was noteworthy that cytoplasmic CHMP1B-L–GFP assemblies were also present at sites where nucleating DNA could not be detected by SPY555-DNA staining. Nevertheless, formation of these CHMP1B-L–GFP foci still required DNA transfection (Extended Data Fig. 3b), indicating either that they nucleated around DNA templates that were too small to be detected by SPY555-DNA (for example, short isolated tracks of naked DNA) or that the presence of cytoplasmic DNA induced a signal that promoted CHMP1B-L–GFP clustering, even in the absence of nucleating DNA (for example, through protein phosphorylation). In foci where DNA was detectable, an outer CHMP1B-L–GFP layer surrounded a central DNA core (Extended Data Fig. 3c) and the two layers partially overlapped at sites where CHMP1B-L–GFP could be contacting the surface of the DNA mass directly. Thus, the presence of DNA in the cytoplasm induces formation of CHMP1B-containing foci that can either surround clustered DNA or lack DNA detectable with SPY555-DNA. Intriguingly, NE components also surround transfected DNA in the cytoplasm, thus raising the possibility that DNA-bound ESCRT complexes may help assemble stable NE-derived protective structures, also termed exclusomes^56^, both in the cytoplasm and in the persisting DNA bridge, although further investigation is required to explore this model.

### CHMP1B and IST1 copolymerize around nucleic acids

**Extended Data Fig. 4:**
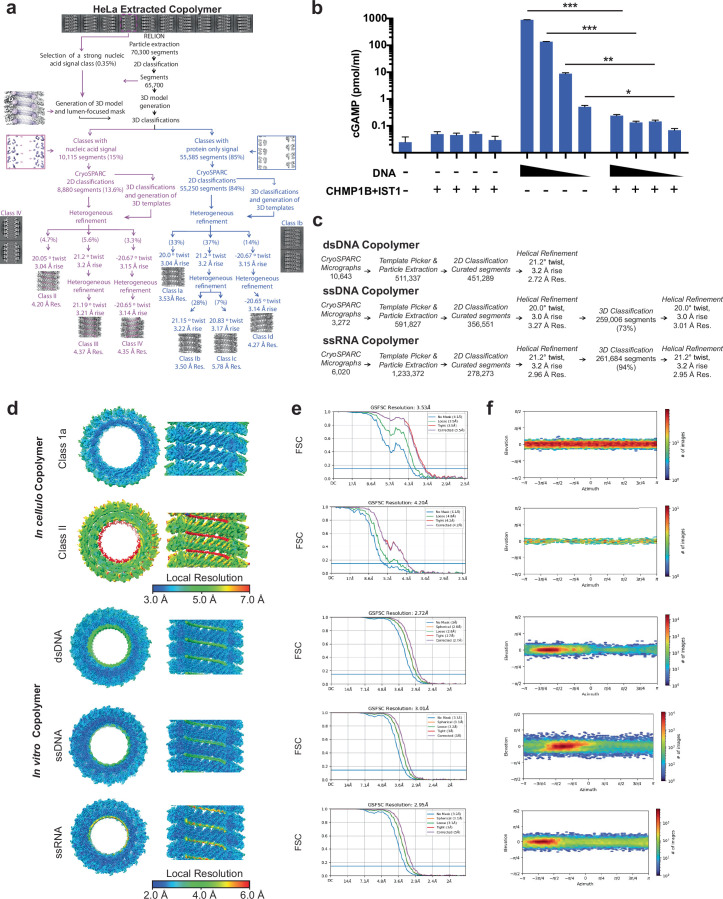
Cryo-EM reconstruction pipelines, validation, and cGAS sensing. **a**, Cryo-EM data processing pipeline for CHMP1B/IST1 filaments isolated from HeLa cells. Final symmetry values and resolutions for the different reconstructions are indicated. **b**, CHMP1B-IST1 filaments protect dsDNA from cGAS innate sensing. *In vitro* sensing assay for CHMP1B + IST1 (each 20 µM) in the presence or absence of linearized dsDNA puc19 vector (79 ng). CHMP1B + IST1 was incubated with DNA for 12 h at 23 C, and samples were then incubated with recombinant cGAS for 8 h at 37 C. Equivalent reactions were prepared with protein alone (CHMP1B + IST1), dsDNA alone, and dsDNA + CHMP1B + IST1 (4-fold serial dilutions). 2´3´-cyclic GMP-AMP (cGAMP) levels were determined by ELISA. Graphs depict mean ± SD from 3 samples from a representative experiment (n = 3). **c**, Cryo-EM data processing pipeline for nucleic acid-bound CHMP1B/IST1 filaments formed *in vitro*. Workflows for the dsDNA-, ssDNA-, and ssRNA-bound filaments, with final symmetry values and resolutions for the different reconstructions indicated. **d**, Local resolution estimates for the final helical refinements. Resolution estimates of reconstructions of CHMP1B/IST1 filaments extracted from cells ranged between 3.0-7.0 Å (top two panels) and nucleic acid-nucleated co-polymers formed *in vitro* ranged between 2.0-6.0 Å (bottom three panels). **e**, Cryo-EM resolution estimates. Gold standard Fourier shell correlations (FCS) for indicated reconstructions. Blue horizontal lines indicate FSC 0.143 threshold. **f**, Particle view distributions. Heat maps showing the angular distributions of particle views calculated in cryoSPARC for the indicated reconstructions. Statistical analysis performed in **b**, unpaired two-tailed t-test with Welch’s correction. Error bars in **b** represent mean ± SD. *P* > 0.05 was considered not significant (ns), while *P* ≤ 0.05 (*), *P* ≤ 0.01(**), *P* ≤ 0.001(***), *P* ≤ 0.0001 (****) were considered significant. Source data

To characterize the cellular ESCRT-III–DNA complexes in greater detail, we cotransfected HeLa cells with overexpression plasmids for CHMP1B and IST1. Remarkably, the cytoplasm of cotransfected cells filled with filamentous tubes that resembled the double-stranded helical CHMP1B–IST1 structure previously determined for recombinant CHMP1B and IST1 assemblies in vitro^53,57^ (Fig. 3b and Extended Data Fig. 3d). For high-resolution analyses, the soluble cellular CHMP1B–IST1 tubes were enriched from cell lysates by differential centrifugation, imaged by cryo-electron microscopy (cryo-EM), segmented, sorted into different classes and reconstructed in three dimensions (3D) (Fig. 3c–e and Extended Data Fig. 4a). These analyses showed that most of the cellular CHMP1B–IST1 tubes corresponded to a family of related one-start double-stranded helical CHMP1B–IST1 tubes that matched the tubes formed by the two pure recombinant proteins in vitro, either in isolation or surrounding acidic membranes^53,57,58^. The cellular structures had an outer strand of IST1 subunits that adopt the closed configuration, an inner strand of CHMP1B subunits that adopt the open configuration and a hollow interior (classes Ia–Id, 85% of the overall population, both right-handed and left-handed one-start helices, 17–18 subunits per turn) (Fig. 3d and Extended Data Fig. 4a).

Three additional distinct 3D classes of tubes were also observed at lower frequencies (class II, 4.7% overall; class III, 4.7% overall; class IV, 2.6% overall) (Extended Data Fig. 4a). A 3D reconstruction at 4.2-Å resolution revealed that class II tubes resembled class Ia tubes (right-handed one-start helix, rise = 3.04 Å, twist = 20.05°) but they also contained strong additional internal density that tracked along the groove between the rungs of the CHMP1B strand (magenta in Fig. 3e). Class III tubes also contained internal density and resembled class Ib/c tubes (right-handed one-start helix, rise = 3.21 Å, twist = 21.19°, resolution = 4.4 Å). Lastly, class IV tubes again contained internal density and resembled class Id tubes (left-handed one-start helix, rise = 3.14 Å, twist = −20.65°, resolution = 4.3 Å). Thus, overexpressed CHMP1B and IST1 spontaneously coassemble in cells and a subset of the tubular assemblies contain strong internal densities that track along the basic groove formed between adjacent rungs of the CHMP1B strands.

**Fig. 4:**
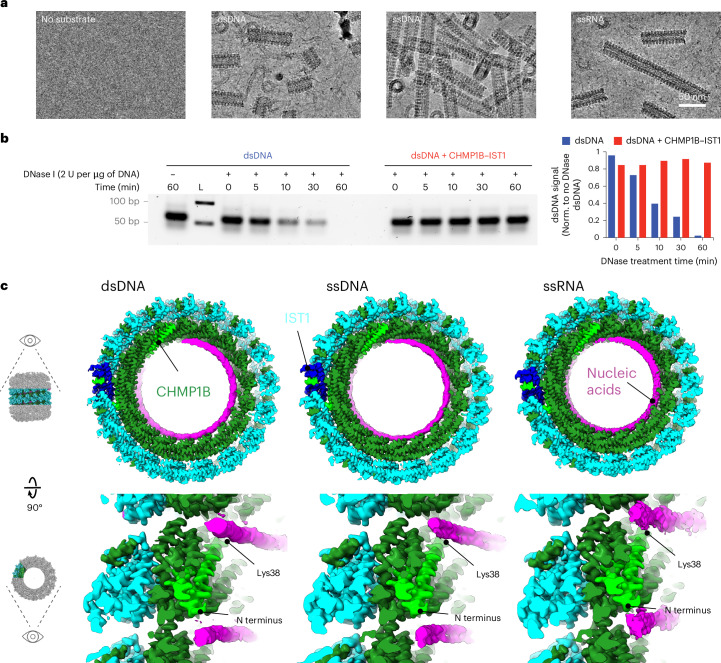
IST1–CHMP1B copolymers encapsulate nucleic acids. **a**, Nucleic acids induce the assembly of IST1–CHMP1B copolymers. Cryo-EM micrographs of CHMP1B–IST1 incubated in the presence of dsDNA, ssDNA or ssRNA at protein-to-base/base pair molar ratios of 1:20. Left, CHMP1B–IST1 filaments do not form in the absence of nucleic acids at these solution conditions (125 mM NaCl and 50 mM Tris pH 8.0). Scale bars, 50 nm. **b**, Copolymerization with CHMP1B and IST1 protects nucleic acids from nuclease digestion. DNase I-treated dsDNA 60-mer oligo mixed with or without CHMP1B and IST1. Left, quenched reactions were separated by agarose gel electrophoresis (SYBR green dye). L, marker ladder (sizes as indicated). Right, densitometry analysis of the DNase-treated dsDNA samples signal (normalized to the untreated dsDNA, lane 1). **c**, Cryo-EM reconstructions of the indicated nucleic-acid-induced assemblies. The 3D reconstructions of dsDNA-templated, ssDNA-templated and ssRNA-templated CHMP1B–IST1 filaments viewed down the helix axis (top) or as a slice through the side view (bottom). CHMP1B (green; light green highlights one monomer) forms the inner strand, while IST1 (cyan; dark blue highlights one monomer) forms the outer strand. Nucleic acid density (magenta) and key CHMP1B-interacting residues are indicated.

We hypothesized that the internal densities in the cellular class II–IV CHMP1B–IST1 tubes might correspond to bound nucleic acid polymers. To test this idea, we evaluated the effects of nucleic acids on CHMP1B–IST1 filament assembly in vitro under stringent dilution and ionic strength conditions in which filaments did not form spontaneously. As shown in Fig. 4a, CHMP1B–IST1 filament formation was induced by random sequence oligonucleotides comprising double-stranded DNA (dsDNA; 60 bp), ssDNA (60 nt) or single-stranded RNA (ssRNA; 60 nt). To determine whether the CHMP1B and IST1 filaments were coassembling around the nucleic acid templates, we tested whether CHMP1B–IST1 filaments could protect dsDNA from DNase I digestion and found that the DNA-templated CHMP1B–IST1 filaments protected both short (60 bp) and plasmid-length dsDNAs^59^ (Fig. 4b). Similarly, DNA-bound CHMP1B–IST1 filaments also prevented dsDNA from activating the innate immune sensor cGAS (Extended Data Fig. 4b). Thus, nucleic acids promote formation of CHMP1B–IST1 copolymers that can protect DNA against nuclease digestion and cGAS recognition.

**Extended Data Fig. 5:**
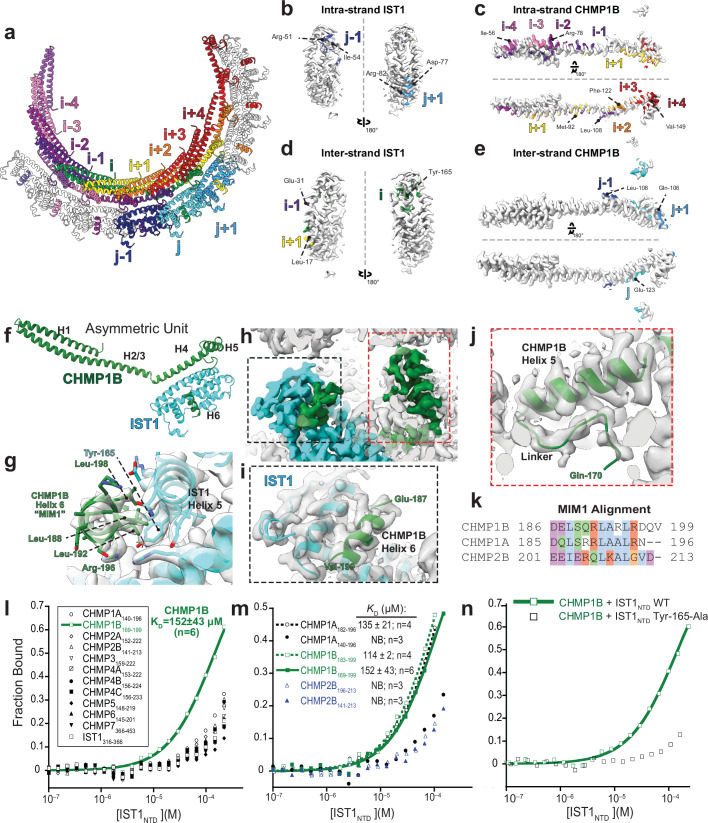
CHMP1B/IST1 filament structure and interactions. **a**, Homo- and hetero-molecular interactions of CHMP1B and IST1 within the dsDNA-nucleated filament. Ribbon diagram of domain swapped CHMP1B ‘i’ (CHMP1B_i_) and IST1 ‘j’ (IST1_j_) subunits within the assembled dsDNA-containing CHMP1B/IST1 filament. The image shows a partial helical turn (9 subunits) of the CHMP1B-IST1 copolymer with contacting subunits above and below the central ‘i’ and ‘j’ molecules (different colors). **b**, Intra-strand IST1 interactions (>3 Å) within the filament mapped onto the IST1_j_ subunit. IST1_j-1_ interacts with N-terminal residues of helix 2 of IST1_j_ at Arg-51 and Ile-54. IST1_j+1_ contacts C-terminal residues of the opposing side of helix 2 on IST1_j_ near Arg-82 and Asp-77. **c**, Intra-strand CHMP1B interactions mapped onto surface density of the CHMP1B_i_ subunit. Eight CHMP1B molecules (i-4 to i + 4) contacts the CHMP1B_i_ subunit. Major contact residues are indicated on the isolated CHMP1B_i_ density. **d**, Inter-strand IST1-CHMP1B interactions mapped onto surface density of the IST1_j_ subunit. CHMP1B_i_, CHMP1B_i-1_, and CHMP1B_i+1_ contact the IST1_j_ molecule. CHMP1B_i_ interactions on IST1_j_ occur in a hydrophobic pocket between the IST1_j_ helix 1/2 hairpin and perpendicular helix 5 near IST1_j_ residue Tyr-165. **e**, Inter-strand IST1-CHMP1B interactions mapped onto surface density of the CHMP1B_i_ subunit. CHMP1B_i_ helix 4/5 contact three IST1 subunits (j-1, j, and j + 1). CHMP1B_i_ helix 6 primarily contacts IST1_J_. **f**, CHMP1B and IST1 asymmetric unit. Inner (CHMP1B) and outer (IST1) strands of the double-stranded filament are interwoven via the flexible linker between CHMP1B helix 5 and helix 6 that connects to the IST1 monomer closest to CHMP1B helix 4. **g**, C-terminal CHMP1B MIM1 helix 6 contacts IST1. Hydrophobic residues Leu-188, Leu-192, Leu-198 along CHMP1B helix 6 (residues 186-199, Microtubule-Interacting Motif 1, MIM1) contact IST1 helix 5, including Tyr-165. **h-j**, CHMP1B MIM1 helix 6 resides on the tube exterior. View through the CHMP1B/IST1 filament between protein layers, highlighting positions of CHMP1B helix 5 (j) and helix 6 (i). A low contour threshold of the symmetrized map shows a structured loop extending from the CHMP1B helix 5 C-terminus to Gln-170, followed by untraceable density until residue Glu-187 (j). **k**, ESCRT-III MIM residue sequence sequence alignment highlighting chemically similar residues in CHMP1B, CHMP1A, and CHMP2B MIM1 helices. **l**, The IST1 N-terminal domain (NTD) binds specifically to the C-terminal end of CHMP1B. Fluorescence polarization binding isotherms for IST1_NTD_ binding to C-terminal peptides from all 12 human ESCRT-III proteins. Curves are averages of three independent experiments and only CHMP1B_169-199_ (K_D_ = 152 ± 43 μM) binds with a K_D_ < 400 μM. **m**, IST1_NTD_ binds specifically to the C-terminal helix 6 MIM1 elements of CHMP1A and CHMP1B. Fluorescence polarization binding isotherms for short and long CHMP1A/1B/2B peptides. Note that CHMP1A_182-196_ MIM1 helix binds IST1_NTD_ better than a longer CHMP1A_140-196_ construct, explaining the lack of binding for the CHMP1A construct in panel l. Curves are averages from numbers of experiments indicated in the legend. **n**, IST1 Tyr-165 is critical for CHMP1B MIM binding. Fluorescence polarization binding isotherms for CHMP1B_169-199_ interacting with IST1_NTD_ WT (green) or IST1_NTD_ Y165A (black). Curves are averages of three independent experiments. The binding experiments in panels l-n show that the CHMP1B MIM1 element binds IST1 in solution.

The three different CHMP1B–IST1–nucleic acid complexes formed in vitro were imaged by cryo-EM and reconstructed at high resolution (Fig. 4c and Extended Data Fig. 4c–f), which allowed us to build atomic models of the protein filaments. Recombinant CHMP1B proteins used for the in vitro assemblies contained a Met136Val substitution that retains the ability of CHMP1B to coassemble with IST1 into filaments, similar to the wild-type consensus sequence (UniProt Q7LBR1) and a variant used in previous work^53,59^. All three complexes formed tubes comprising one-start, right-handed, double-stranded filaments with strong internal densities for the nucleic acid (magenta in Fig. 4c and Extended Data Fig. 4c,d). This extraluminal density was positioned at the same location as that seen in the cellular assemblies (Fig. 3e). The ssDNA and dsDNA copolymers exhibited similar helical parameters and charge density maps (dsDNA, rise = 3.025 Å, twist = 20.034°, Resolution=2.7 Å; ssDNA, rise = 3.028 Å, twist = 20.047°, resolution = 3.0 Å), whereas the ssRNA copolymer formed a narrower tube with distinct helical symmetry (rise = 3.203 Å, twist = 21.180°, resolution = 3.1 Å). Full details of all the interprotein contacts for the filaments formed by CHMP1B–IST1 around dsDNA are provided in Extended Data Fig. 5a–e. In all three cases, the nucleic acid strands tracked along the groove between adjacent rungs of the CHMP1B strand, sandwiched between the basic CHMP1B N terminus and basic residues along CHMP1B helices 1 and 2 (Fig. 4c).

The molecular models revealed that the C terminus of CHMP1B snorkeled through the IST1 layer to the filament exterior (Extended Data Fig. 5f,g). This allowed the C-terminal α6 helix (also known as the type I MIT-interacting motif, MIM1) to contact IST1, as previously observed in the membrane-bound version of the CHMP1B–IST1 copolymers^57^ (Extended Data Fig. 5f–j). We showed that this interaction was specific by testing whether isolated MIM peptides from all 12 human ESCRT-III proteins could bind the IST1 N-terminal domain (IST1_NTD_; residues 1–189) and found that only the minimal CHMP1B MIM1 peptide bound with a dissociation constant of <400 µM in a fluorescence polarization anisotropy assay (Extended Data Fig. 5l). Extending the minimal MIM1 peptide sequences of CHMP1B and CHMP1A further increased their binding affinity to IST1_NTD_ (to <150 µM), whereas a longer control MIM peptide from CHMP2B still failed to bind (Extended Data Fig. 5k,m). A hydrophobic residue on IST1 α5 (Tyr165) is buried by CHMP1B α6 in the CHMP1B–IST1 copolymers (Extended Data Fig. 5g). We confirmed that this residue contributes to the CHMP1B MIM–IST1 interaction because the IST1 Tyr165Ala substitution eliminated CHMP1B MIM1 binding (as compared to wild-type CHMP1B; Extended Data Fig. 5n). Thus, the C-terminal MIM motifs of both CHMP1 proteins make specific contacts with IST1, as seen in our structures of CHMP1B–IST1–nucleic-acid copolymers, and these contacts would presumably need to be relieved for the CHMP1 MIM elements to bind VPS4 proteins^60^.

**Extended Data Fig. 6:**
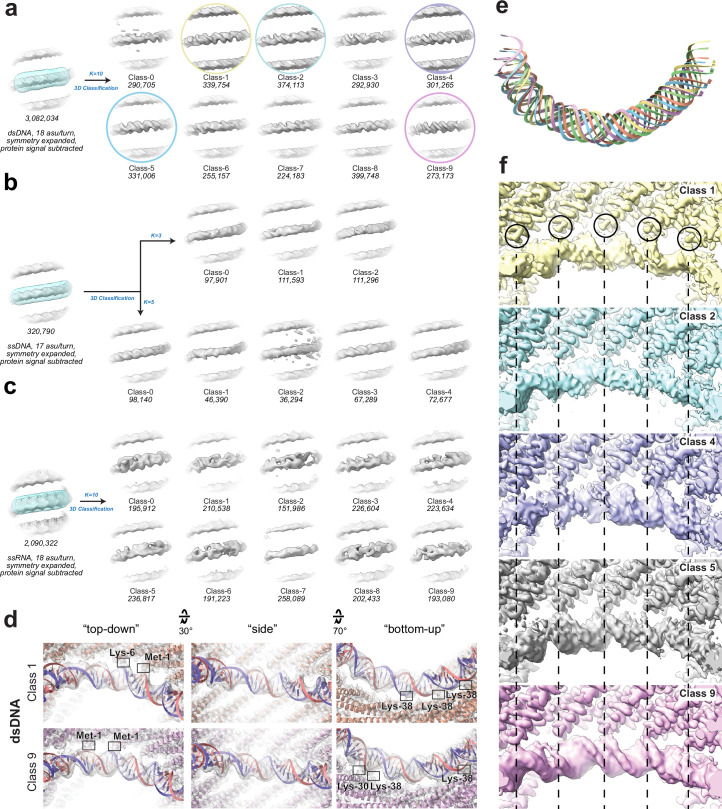
Focused nucleic acid classifications. **a**, Focused classifications of dsDNA density. Focused classification of the dsDNA density with protein signal subtracted into ten classes. Classification mask (cyan) indicates the focused reconstruction area. Identity and symmetry expanded particle numbers indicated below each class. Classes highlighted with colored circles exhibited extended stretches of density with strong dsDNA features (see panel f). Views of Class 1 and Class 9 are shown in panel d. **b**, Two focused classifications of ssDNA density. Focused classification of the ssDNA density with protein signal subtracted into three (k = 3) or five (k = 5) classes. Classification mask (cyan) indicates the focused reconstruction area. Identity and symmetry expanded particle numbers indicated below each class. **c**, Focused classifications of ssRNA density. Focused classification of the ssRNA density with protein signal subtracted into ten classes. Classification mask (cyan) indicates the focused reconstruction area. Identity and symmetry expanded particle numbers indicated below each class. **d**, Focused classification of dsDNA density within the CHMP1B/IST1 assembly. Protein signal subtraction and mask-focused classification on segments of the nucleic acid strands from classes 1 and 9 (see panel a) restores signal from dsDNA as reconstructed from the original, non-subtracted image, together with CHMP1B and dsDNA molecular models. Interacting residues are indicated. **e**, Overlay of fitted dsDNA models. Models of poly-AT dsDNA flexibly fit into the density for the different classes from panel a. Neighboring registers are shifted by approximately 3.4 Å. **f**, N-terminal CHMP1B density within different dsDNA CHMP1B/IST1 focused classifications. Black circles highlight the aligned N-termini of each chain of the CHMP1B density aligned across 5 classes from the focused cryo-EM dsDNA reconstructions (from panel a). Note that the N-terminal densities are consistently positioned near dsDNA backbone phosphates from the bound dsDNA helices (dashed lines across classes).

The nucleic acid polymers did not follow the helical symmetry of the protein filaments and, consequently, they appeared as strong but featureless densities in 3D reconstructions in which particle alignment was driven by the high symmetry of the protein coat (Fig. 4c). To resolve the nucleic acid and CHMP1B contacts in greater detail, we used a focused classification, signal subtraction and segmental reconstruction procedure (Extended Data Fig. 6a–c). This analysis recovered the dimensions of dsDNA, including the major and minor grooves of the double helix. Modeling dsDNA into the individual dsDNA classes (1, 2, 4, 5 and 9) in a fixed protein register showed a continuum of nucleic-acid-binding conformations in subclassified focused refinements (Extended Data Fig. 6d–f). Similar analysis of the ssDNA-templated and ssRNA-templated filaments showed single-stranded nucleic acid density in the same helical groove as the dsDNA substrate but major molecular features of the ssDNA or ssRNA could not be confidently modeled (Extended Data Fig. 6b,c). These data indicate that the nucleic acid–ESCRT interactions are driven by flexible, long-range electrostatics rather than a defined structural periodicity.

**Extended Data Fig. 7:**
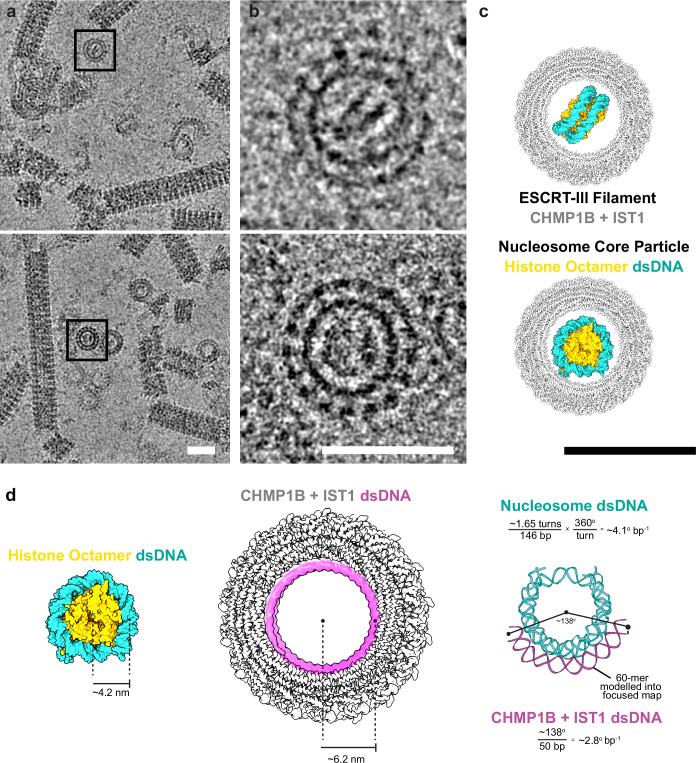
Nucleosomes within CHMP1B/IST1 filaments. **a**, CryoEM micrographs of nucleosome-templated CHMP1B/IST1 filaments. Multiple low pass filtered cryoEM micrographs of CHMP1B/IST1 filaments templated with dsDNA-wrapped dinucleosomes. **b**, Expanded views of individual nucleosome-templated CHMP1B/IST1 filaments. Expanded views show CHMP1B/IST1 filaments with lumenal densities (black boxes panel **a**) that resemble nucleosomes (see panel **c**). **c**, Structures of nucleosome within the lumens of the CHMP1B/IST1 filament. Nucleosomes with dsDNA (teal) wrapped histone core (yellow) (PDB ID:3AV2) modeled into the density from CHMP1B/IST1 only ESCRT-III filaments (EMD-6461). Nucleosomes are oriented to resemble the views from panel **b**. **d**, DNA curves less tightly within CHMP1B/IST1/dsDNA structure than in nucleosomal DNA. The figure shows that dsDNA coiled within the CHMP1B/IST1 filament (magenta) adopts a slightly more relaxed radius of curvature than dsDNA within a nucleosome (teal) (PDB ID:3AV2). Scale bars in **a-c** indicate 25 nm.

When UFBs form in cells, a complex set of cofactors can bind both the single-stranded and double-stranded segments, including RPA, PICH, BLM and nucleosomes. To determine whether ESCRT-III filaments of CHMP1B and IST1 could accommodate such bulky genomic substrates, we reconstituted CHMP1B–IST1 filaments in the presence of dsDNA-wrapped dinucleosomes. Using the same stringent salt conditions that prevent untemplated CHMP1B–IST1 filament assembly, we tested whether dinucleosomes could template helical ESCRT-III filament assembly at molar ratios of 1 dinucleosome to 100 monomers of CHMP1B–IST1. Cryo-EM analyses revealed that 25-nm-diameter filaments formed (Extended Data Fig. 7a) and numerous end-on views (~4 per micrograph) showed extra density within the lumens (Extended Data Fig. 7b). Hence, nucleosomes can be accommodated within the ESCRT-III filament. Geometrical modeling using a histone core (PDB 3AV2) (Extended Data Fig. 7c) further supported the idea that CHMP1B–IST1 luminal dimensions can accommodate nucleosomal dsDNA^61^. Indeed, dsDNA is more tightly wound in nucleosomes (radius of curvature: 4.2 nm; 4.1° per bp) than within helical CHMP1B–IST1 filaments (6.2 nm, 2.8° per bp) and modeled nucleosomes fit snugly within CHMP1B–IST filament lumens (Extended Data Fig. 7d).

These experiments revealed that, although CHMP1B and IST1 are best understood as membrane-binding or membrane-remodeling factors^53,57,62–67^, they can also unexpectedly copolymerize around nucleic acids. CHMP1B–IST1 filaments formed distinct helical tubes around DNA versus RNA, with different numbers of protein subunits per turn; ~18 for DNA templates versus ~17 for RNA templates (Fig. 4c and Extended Data Fig. 4c,d). Importantly, the helical parameters and internal densities of the templated CHMP1B–IST1 tubes formed in vitro matched those of the class II (DNA) and class III (RNA) tubes formed in transfected cells (Fig. 3e and Extended Data Fig. 4a). We did not observe left-handed class IV tubes in our in vitro assembly reactions, although analogous left-handed CHMP1B–IST1 helical tubes can form around lipids in vitro^57^. Lastly, we found that dinucleosome constructs stimulate CHMP1B–IST1 assembly and that the luminal dimensions of CHMP1B–IST1 tubes can accommodate nucleosomes (Extended Data Fig. 7a–c). In summary, CHMP1B–IST1 filaments can assemble around both DNA and RNA templates in cells and in vitro and we hypothesize that the dsDNA template found within cellular class II tubes came from cellular nucleic acids, perhaps including transfected plasmid DNA.

### ESCRT-III subunits transfer onto mis-segregated DNA at the reforming nucleus

**Fig. 5:**
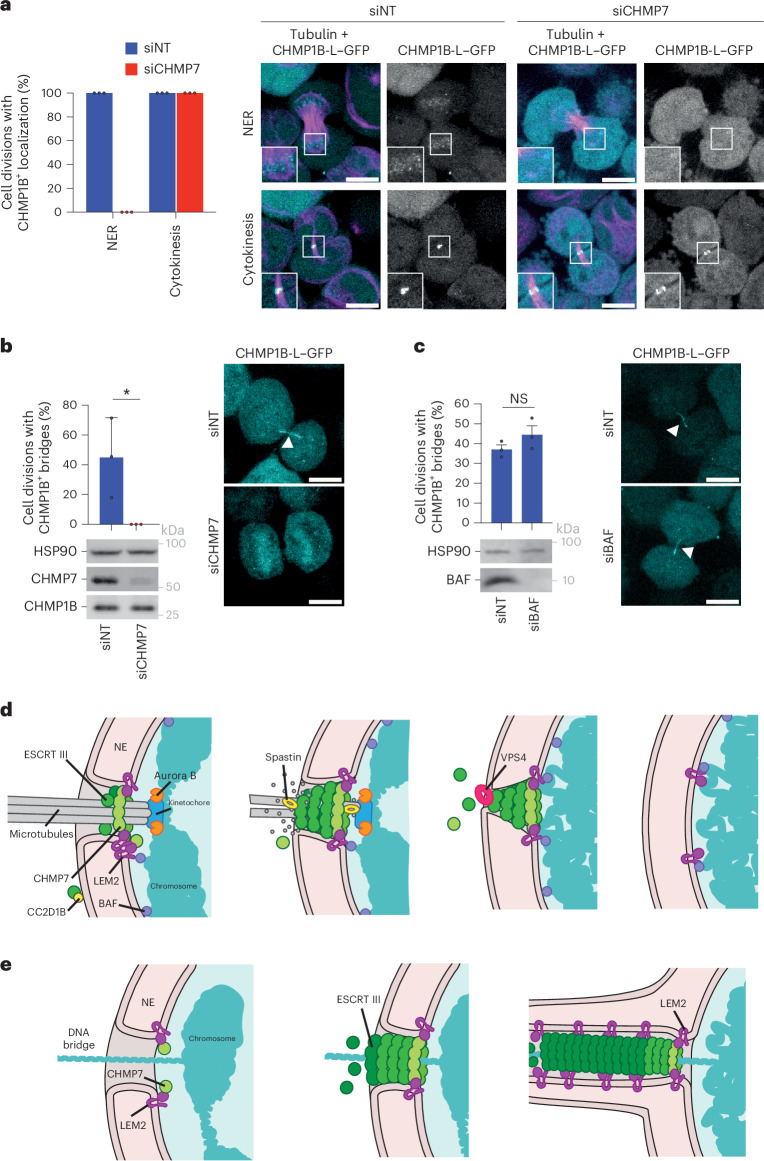
Transfer of ESCRT-III to UFBs initiates at the reforming NE. **a**, CHMP7 depletion with siRNA (siCHMP7) prevents CHMP1B recruitment to the reforming NE but does not alter recruitment to the midbody. HeLa^CHMP1B-L–GFP^ cells were transfected with the indicated siRNAs, incubated with SiR–tubulin (magenta) and treated with 80 nM ICRF-193 for 1 h before imaging. Quantification of CHMP1B-L–GFP (cyan) localization to the reforming NE and the midbody during mitosis (siNT, *n* = 29 cells; siCHMP7, *n* = 24 cells). **b**, Transfer of CHMP1B to UFBs requires localization to the nascent NE. HeLa^CHMP1B-L–GFP^ cells were transfected with the indicated siRNAs and treated with 80 nM ICRF-193 for 1 h before imaging. Quantification of cells with CHMP1B-L–GFP-positive bridges (white arrowhead) that formed during each cell division (siNT, *n* = 29 cells; siCHMP7, *n* = 24 cells). Western blot analysis using the indicated antibodies. **c**, Mitotic CHMP1B bridge formation does not require BAF. HeLa^CHMP1B-L–GFP^ cells were treated with siNT (*n* = 34 cells) and siBAF (*n* = 37 cells). Cells that failed to divide and/or localize CHMP1B-L–GFP to the reforming NE were excluded. **d**, Model for normal closure of postmitotic NE fenestrations during late anaphase. The panels illustrate the stepwise action of ESCRT and associated cofactors in removing spindle microtubules and closing NE fenestrations. **e**, Model for initiation of a protective sleeve surrounding a mis-segregated DNA bridge at the closing NE. The panels illustrate how ESCRT-III subunits and the extended nuclear membrane could begin to encase mis-segregated DNA bridges. Scale bars, 10 µm (**a**–**c**). Representative images in **a**–**c** are from three independent experiments. Statistical analysis was conducted using an unpaired two-tailed *t*-test (**b**) or paired two-tailed *t*-test (**c**). Error bars in **b**,**c** represent the mean ± s.d.; independent experiments are plotted as dots. *P ≤ 0.05, **P ≤ 0.01, ***P ≤ 0.001 and ****P ≤ 0.0001. Source data

The studies described above established that ESCRT-III associates with unresolved UFBs after mitotic NER and that DNA can nucleate formation of CHMP1B–IST1 filaments that surround the DNA. Therefore, we reasoned that ESCRT-III might transfer from sites of NE hole closure onto mis-segregated DNA when reforming NE encircles a UFB rather than spindle microtubules. To test this idea, we selectively interfered with ESCRT-III recruitment to the reforming NE by depleting CHMP7, which is the specialized ESCRT factor that initiates recruitment of downstream ESCRT-III subunits to NE holes following activation by the INM protein LEM2 (refs. ^24–26^). As expected, CHMP7 depletion inhibited CHMP1B-L–GFP recruitment to the reforming NE but did not alter recruitment of CHMP1B-L–GFP to the midbody during cytokinesis (Fig. 5a). Critically, CHMP7 depletion also completely abrogated CHMP1B-L–GFP incorporation into unresolved UFBs (Fig. 5b), demonstrating that transfer of CHMP1B to UFBs requires recruitment to the nascent NE through LEM2–CHMP7.

**Extended Data Fig. 8:**
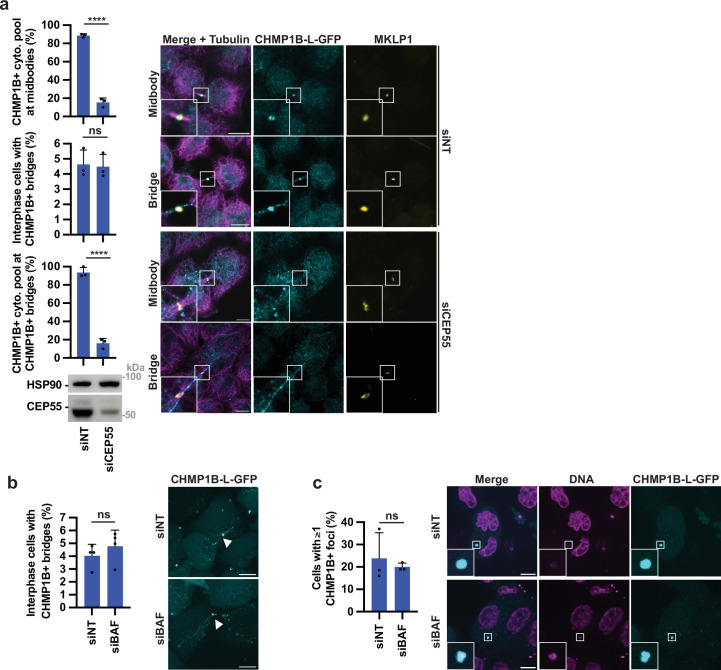
Midbody origins of the CHMP1B cytokinetic pool and demonstration that BAF is dispensable for forming CHMP1B interphase bridges and for clustering on transfected DNA. **a**, Midbody origin of the CHMP1B cytokinetic pool at persistent UFBs. HeLa^CHMP1B-L-GFP^ cells were transfected with siNT or siCEP55 and subsequently treated with 80 nM ICRF-193 for 24 h. Cells were then fixed and stained with anti-GFP (cyan), anti-MKLP1 (yellow) and anti-tubulin (magenta) antibodies. Top-left panel: Quantification of CHMP1B-L-GFP cytokinetic pools at midbodies (siNT n = 61 cells, siCEP55 n = 64 cells). Middle-left panel: Quantification of interphase cells connected by CHMP1B-L-GFP positive bridges (siNT n = 324 cells, siCEP55 n = 334 cells). Bottom-left panel: Quantification of persistent UFBs with CHMP1B-L-GFP cytokinetic pools (siNT n = 31 cells, siCEP55 n = 31 cells). Right panels show representative cell images. **b**, CHMP1B bridges do not require BAF to form or persist during interphase. HeLa^CHMP1B-L-GFP^ cells were transfected with the indicated siRNAs and treated with 80 nM ICRF-193 for 1 h before imaging. Quantification of interphase cells (siNT n = 2058 cells; siBAF n = 2034 cells) with CHMP1B-L-GFP (cyan) positive bridges (white arrowhead). **c**, CHMP1B clusters containing transfected DNA do not require BAF to form. HeLa^CHMP1B-L-GFP^ cells were co-transfected with pCR3.1 and the indicated siRNAs, followed by labelling with SPY555-DNA (magenta). Quantification of cells (n = 400 cells in both siNT and siBAF) containing one or more CHMP1B-L-GFP (cyan) positive foci. Representative images in **a,c** are from 3 independent experiments, while **b** is from 4 independent experiments. Scale bars in **a-c** are 10 µm. Statistical analysis performed in **a-c**, unpaired two-tailed t-test. Error bars in **a-c** represent mean ± SD, independent experiments are plotted as dots. *P* > 0.05 was considered not significant (ns), while *P* ≤ 0.05 (*), *P* ≤ 0.01(**), *P* ≤ 0.001(***), *P* ≤ 0.0001 (****) were considered significant. Source data

Cytokinetic cells that contain persistent UFBs contain two localized pools of CHMP1B protein: a ‘UFB pool’ that crosses the midbody and connects the nuclei and a separate ‘cytokinetic pool’ that localizes to the central region of the midbody and colocalizes with MKLP1, a component of the centralspindlin complex that facilitates abscission of the plasma membrane^68^ (Extended Data Fig. 8a). To test whether these pools originate independently, we depleted CEP55, which recruits early-acting ESCRT factors to the midbody and is, therefore, required for assembly of cytokinetic ESCRT-III filaments^29,30,34^. As expected, recruitment of CHMP1B-L–GFP to the midbody was inhibited in CEP55-depleted cells, whereas the transfer of CHMP1B-L–GFP to persistent UFBs was not altered by this treatment (Extended Data Fig. 8a). The cytokinetic pool of ESCRT-III remained associated with persistent UFBs after the midbody tubulin was disassembled, as shown by the subset of CHMP1B that colocalizes with MKLP1 in these bridges (Extended Data Fig. 8a). However, this MKLP1-associated CHMP1B-L–GFP in interphase DNA bridges was selectively eliminated upon CEP55 depletion, consistent with its distinct midbody origin (Extended Data Fig. 8a). Therefore, a separate DNA-associated pool of CHMP1B coexists at interphase UFBs with an ESCRT-III pool that is recruited by midbody CEP55 during cytokinesis (Extended Data Fig. 8a).

We also tested whether CHMP1B incorporation into persistent UFBs requires BAF, a nuclear factor that connects dsDNA from chromatin and INM-embedded LEM proteins during NER^69–71^. In BAF-depleted cells, an LEM2–CHMP7 redundant mechanism facilitates NER^72^. Importantly, BAF depletion by siRNA did not alter the formation of CHMP1B-L–GFP bridges during cell division or interphase (Fig. 5c and Extended Data Fig. 8b). Similarly, CHMP1B-L–GFP retained its association with transfected DNA in BAF-depleted cells (Extended Data Fig. 8c), thus excluding indirect associations of ESCRT-III and DNA through BAF on persistent UFBs or transfected DNA. These results support a model whereby ESCRT-III meets persistent UFBs at the reforming NE and then forms a protective coat around the mis-segregated DNA (Fig. 5d,e).

### NoCut requires CHMP1B to protect cells against unresolved UFBs

**Fig. 6:**
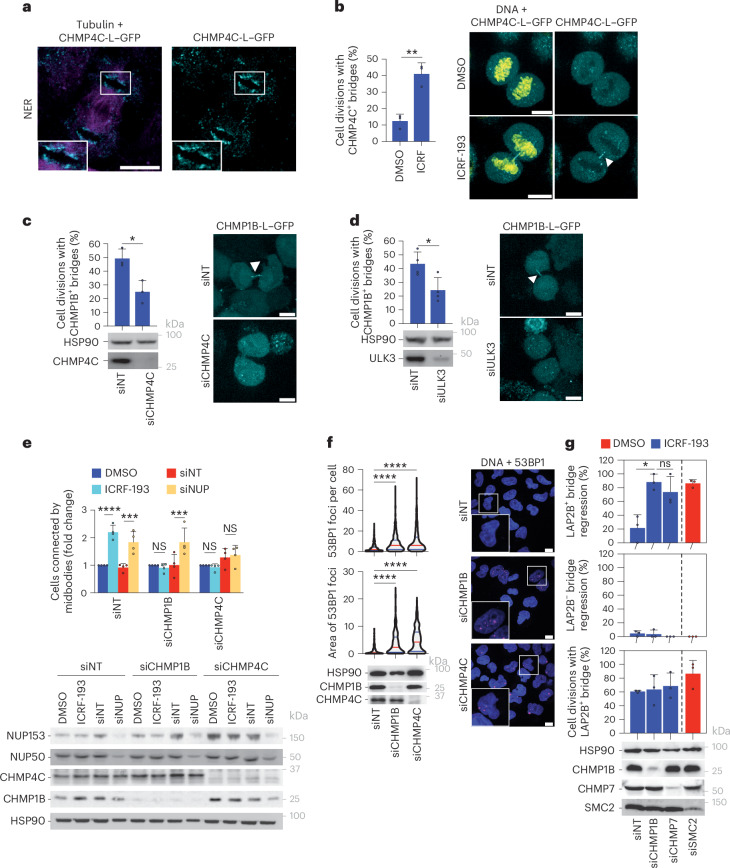
NoCut checkpoint machinery components are required for efficient coating of UFBs by ESCRT-III. **a**, CHMP4C is recruited to the reforming NE during mitosis. HeLa^CHMP4C-L–GFP^ cells were fixed and stained with anti-GFP (cyan) and anti-tubulin (magenta). Representative localization of CHMP4C-L–GFP during NER. **b**, ICRF-193 induces CHMP4C-positive bridges during mitosis. HeLa^CHMP4C-L–GFP^ cells were labeled with SPY555-DNA (yellow) and treated with DMSO (*n* = 40 cells) or 80 nM ICRF-193 (*n* = 38 cells) for 1 h before live-cell imaging. Quantification of CHMP4C-L–GFP-positive bridges (cyan, white arrowhead) that formed during cell division. **c**, CHMP4C is required for the efficient coating of UFBs with CHMP1B during mitosis. HeLa^CHMP1B-L–GFP^ cells were transfected with siNT (*n* = 29 cells) or siCHMP4C (*n* = 36 cells), followed by treatment with 80 nM ICRF-193 for 1 h before live-cell imaging. Quantification of cells with CHMP1B-L–GFP-positive bridges (cyan, white arrowhead) that formed during each cell division. Western blot analysis using the indicated antibodies. **d**, ULK3 is required for efficient coating of UFBs with CHMP1B during mitosis. HeLa^CHMP1B-L–GFP^ cells were transfected with siNT (*n* = 70 cells) or siULK3 (*n* = 43 cells), followed by treatment and quantification as described in panel **c**. Western blot analysis using the indicated antibodies. **e**, CHMP1B is differentially required to sustain NoCut function following different types of mitotic errors. HeLa cells were transfected with siNT, siCHMP1B or siCHMP4C. Cells were subsequently treated with DMSO (siNT, *n* = 1,304 cells; siCHMP1B, *n* = 1,253 cells; siCHMP4C, *n* = 1,271 cells), 160 nM ICRF-193 (siNT, *n* = 1289 cells; siCHMP1B, *n* = 1,271 cells; siCHMP4C, *n* = 1,261 cells) or cotransfected with siNT (siNT, *n* = 1,316 cells; siCHMP1B, *n* = 1,279 cells; siCHMP4C, *n* = 1,305 cells) or nucleoporin siRNA (siNUP) (siNT, *n* = 1,285 cells; siCHMP1B, *n* = 1,297 cells; siCHMP4C, *n* = 1,256 cells) for 24 h before fixation and staining with an anti-tubulin antibody. Quantification of the fold change in percentage of cells connected by midbodies (*n* = 4 independent experiments). Western blot analysis using the indicated antibodies. **f**, CHMP1B protects HeLa cells against DNA damage. HeLa cells were depleted of CHMP1B or CHMP4C (positive control) for 48 h before fixation and staining for 53BP1 foci (a measure of dsDNA breaks). The numbers (top) and areas (bottom) of 53BP1 foci per cell were quantified (siNT, *n* = 470 cells; siCHMP1B, *n* = 422 cells; siCHMP4C, *n* = 451 cells) and representative cell images and expansions are shown (right). Western blot analysis using the indicated antibodies. **g**, CHMP1B and CHMP7 are required to prevent binucleation of cells treated with ICRF-193. HeLa^YFP-LAP2B^ cells were transfected with the indicated siRNAs, then treated with DMSO or 80 nM ICRF-193 for 1 h and imaged every 10 min for 48 h using live-cell microscopy. Top, quantification of cell divisions that had YFP–LAP2B-positive bridges that regressed to form binucleated cells is shown (siNT, *n* = 50 cells; siCHMP7, *n* = 31 cells; siCHMP1B, *n* = 41 cells; siSMC2, *n* = 54 cells). Middle, cell divisions that were not positive for YFP–LAP2B bridges that regressed were scored (siNT, *n* = 40 cells; siCHMP7, *n* = 25 cells; siCHMP1B, *n* = 25 cells; siSMC2, *n* = 6 cells). Bottom, total cell divisions, with and without YFP–LAP2B-positive bridges were also quantified (siNT, *n* = 90 cells; siCHMP7, *n* = 56 cells; siCHMP1B, *n* = 66 cells; siSMC2, *n* = 60 cells). Western blot analysis using the indicated antibodies. Scale bars, 10 µm (**a**–**d**,**f**). Representative images in **a**–**d,f**, are from three independent experiments. Statistical analysis was conducted using an unpaired two-tailed *t*-test (**b**–**d**), Tukey’s multiple-comparison test (**e**,**g**) or Dunn’s multiple-comparison test (**f**). Error bars in **b**–**e**,**g** represent the mean ± s.d.; independent experiments are plotted as dots. *P ≤ 0.05, **P ≤ 0.01, ***P ≤ 0.001 and ****P ≤ 0.0001. Source data

**Extended Data Fig. 9:**
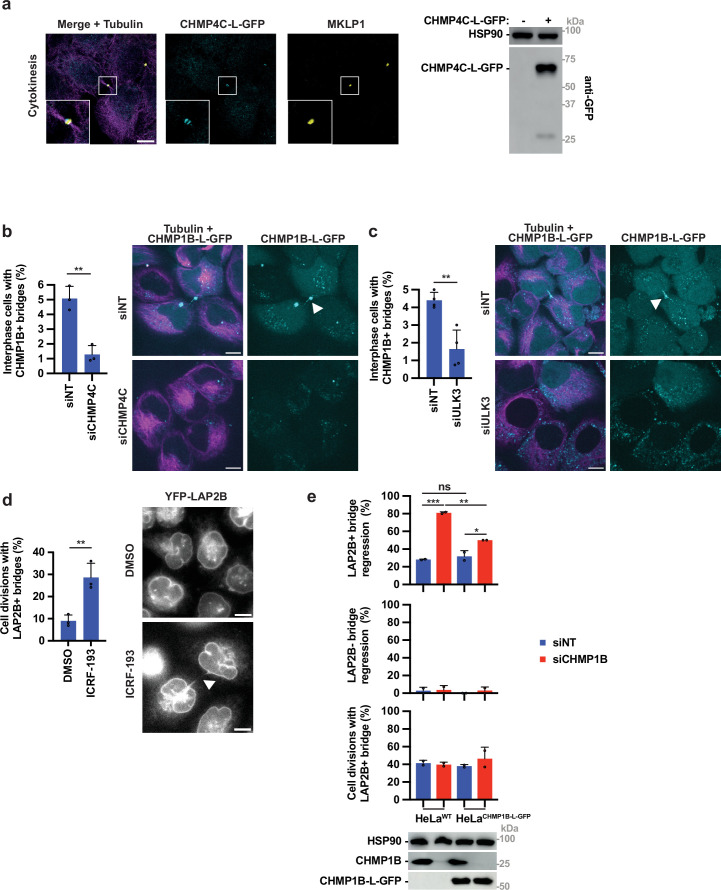
Validation of the HeLa^CHMP4C-L-GFP^ cells and further demonstration that NoCut checkpoint machinery components are required for efficient coating of UFBs by ESCRT-III. **a**, Validation of CHMP4C-L-GFP cells. Left panels: Western blot of HeLa and HeLa^CHMP4C-L-GFP^ cells with the indicated antibodies. Right panel: HeLa^CHMP4C-L-GFP^ cells were fixed and stained with anti-GFP (cyan), anti-MKLP1 (yellow) and anti-tubulin (magenta) antibodies. Images show representative localization of CHMP4C-L-GFP to the midbody during cytokinesis. **b**, CHMP4C is required for efficient CHMP1B coating of persistent UFBs. HeLa^CHMP1B-L-GFP^ cells were transfected with siNT (n = 317 cells) or siCHMP4C (n = 313 cells), treated with 80 nM ICRF-193 for 24 h prior to imaging and incubated with SiR-tubulin (magenta). Quantification of interphase cells connected by CHMP1B-L-GFP bridges (cyan; white arrowhead). **c**, ULK3 is required for efficient CHMP1B coating of persistent UFBs. HeLa^CHMP1B-L-GFP^ cells were transfected with siNT (n = 503 cells) or siULK3 (n = 453 cells), followed by treatment and quantification as described in panel b. **d**, ICRF-193 induces LAP2B positive bridges during mitosis. HeLa^YFP-LAP2B^ cells were treated with DMSO (n = 697 cells) or 160 nM ICRF-193 (n = 600 cells) and imaged by live-cell microscopy for 24 h. Quantification of LAP2B positive bridges (grey; white arrowhead) that formed during mitosis. **e**, CHMP1B-L-GFP rescues binucleation of cells treated with ICRF-193. HeLa^mCh-LAP2B^ and HeLa^CHMP1B-L-GFP mCh-LAP2B^ cells were transfected with siNT or siCHMP1B, then treated with 80 nM ICRF-193 for 1 h, and then imaged every 10 mins for 48 h using live-cell microscopy. Top panel: quantification of midbody regression in cells with mCh-LAP2B bridges (HeLa^mCh-LAP2B^ siNT n = 32 cells; HeLa^mCh-LAP2B^ siCHMP1B n = 21 cells and HeLa^CHMP1B-L-GFP mCh-LAP2B^ siNT n = 22 cells; HeLa^CHMP1B-L-GFP mCh-LAP2B^ siNT n = 20 cells). Middle panel: Quantification of midbody regression in cells that lacked mCh-LAP2B bridges (HeLa^mCh-LAP2B^ siNT n = 46 cells; HeLa^mCh-LAP2B^ siCHMP1B n = 32 cells and HeLa^CHMP1B-L-GFP mCh-LAP2B^ siNT n = 36 cells; HeLa^CHMP1B-L-GFP mCh-LAP2B^ siNT n = 25 cells). Bottom panel: Quantification of cell divisions irrespective of mCh-LAP2B bridge status were also quantified (HeLa^mCh-LAP2B^ siNT n = 78 cells; HeLa^mCh-LAP2B^ siCHMP1B n = 53 cells and HeLa^CHMP1B-L-GFP mCh-LAP2B^ siNT n = 58 cells; HeLa^CHMP1B-L-GFP mCh-LAP2B^ siNT n = 45 cells), demonstrating that the different treatments did not alter LAP2B+ bridge frequency. Western blots with anti-HSP90, anti-CHMP1B and anti-GFP. Representative images in **a,b,d** are from 3 independent experiments, **c** is from 4 independent experiments, and **e** is from 2 independent experiments. Scale bars in **a-d** are 10 µm. Statistical analysis performed in **b-d**, unpaired two-tailed t-test, **e**, Tukey’s multiple comparisons test. Error bars in **b-e** represent mean ± SD, independent experiments are plotted as dots. *P* > 0.05 was considered not significant (ns), while *P* ≤ 0.05 (*), *P* ≤ 0.01(**), *P* ≤ 0.001(***), *P* ≤ 0.0001 (****) were considered significant. Source data

To examine the relationship among UFB formation, ESCRT-III assembly, NoCut surveillance and protection of unresolved UFBs against genome damage, we engineered stable HeLa cells that expressed a fluorescent version of CHMP4C, the specialized ESCRT-III subunit that regulates NoCut signaling^19^. CHMP4C-L–GFP localized to the midbody as expected (Extended Data Fig. 9a) and to the reforming NE (Fig. 6a), suggesting that NoCut surveillance responses could initiate during NER. Like CHMP1B, CHMP4C-L–GFP also decorated UFBs at the end of anaphase and the levels of CHMP4C-L–GFP-labeled UFBs tracked with increased UFB formation induced by ICRF-193 treatment (Fig. 6b and Supplementary Video 3). These observations suggested that CHMP4C recognition of persistent UFBs might also initiate at the reforming NE and lead to subsequent modulation of ESCRT-III polymers at mis-segregated DNA to sustain NoCut activity. In support of this model, siRNA depletion of CHMP4C reduced the frequency of CHMP1B-L–GFP internuclear bridges (Fig. 6c). Similarly, depletion of ULK3, a kinase that regulates NoCut activity by phosphorylating ESCRT-III subunits^44^, also reduced the frequency of dividing cells with CHMP1B-L–GFP bridges (Fig. 6d). Moreover, depletion of either CHMP4C or ULK3 reduced the numbers of interphase cells connected by persistent CHMP1B-L–GFP bridges even more strongly (Extended Data Fig. 9b,c). Thus, at least two ESCRT-associated regulators of the NoCut pathway promote CHMP1B localization and persistence on unresolved UFBs.

To test the idea that CHMP1B itself might also have a role in the NoCut response to mis-segregated DNA, we induced extended NoCut signaling either by partial depletion of the nucleoporins NUP153 and NUP50 or by treatment with ICRF-193 (refs. ^8,73^). As expected, both perturbations increased the proportion of cells connected by midbodies, reflecting cytokinetic delays induced by NoCut activation (Fig. 6e). Importantly, depletion of CHMP1B inhibited NoCut in response to mis-segregated DNA but not in response to NUP depletion. By contrast, control depletion of CHMP4C, the master regulator of ESCRT-dependent checkpoint activity, abrogated NoCut activity in response to both NUP depletion and ICRF-193 treatment. We then assessed genetic damage in CHMP1B‑depleted cells by quantifying nuclear foci marked by the DNA damage response protein 53BP1. CHMP1B knockdown markedly increased the number of DNA lesions and this phenotype was broadly comparable to the elevated DNA damage observed following CHMP4C depletion (Fig. 6f). The 53BP1 foci were also substantially larger in both CHMP1B‑depleted and CHMP4C‑depleted cells (Fig. 6f), consistent with the formation of nuclear bodies previously associated with unresolved UFBs^74^. This protection of the genome by CHMP1B, even in the absence of external perturbations, is consistent with high basal levels of UFBs in HeLa cells (Fig. 1b). Our data demonstrate that CHMP1B is a previously unappreciated component of the NoCut response to DNA bridges.

To analyze NoCut activities further, we assessed cytokinetic outcomes in HeLa cells that stably expressed yellow fluorescent protein (YFP)-tagged LAP2B as a live-cell marker for mis-segregated DNA. As expected, ICRF-193 increased the frequency of cell divisions with YFP–LAP2B bridges (Fig. 6g and Extended Data Fig. 9d). Similarly, SMC2 depletion resulted in a large proportion of cell divisions with visible YFP–LAP2B bridges. However, the cytokinetic outcomes of the two types of bridges differed dramatically. Whereas nearly all cell divisions with YFP–LAP2B bridges led to cytokinesis regression in SMC2-depleted cells, only 20% of these events regressed during cytokinesis when cells were treated with ICRF-193 (Fig. 6g and Supplementary Video 4).

These striking differences correlated with the preferential coating of ICRF-193-induced bridges by CHMP1B, suggesting that the selective association of CHMP1B with unresolved UFBs could protect cells against binucleation, whereas chromatin bridges such as those induced by SMC2 depletion did not trigger this protective response. In support of this model, CHMP1B depletion dramatically increased binucleation to more than 80% in cell divisions exhibiting YFP–LAP2B bridges that were induced by ICRF-193 (Fig. 6g and Supplementary Video 4). Crucially, cell divisions without persistent YFP–LAP2B bridges were scored within the same experiment and CHMP1B depletion did not increase binucleation in this context.

To test whether CHMP1B recruitment to the reforming NE was also required to mount an effective defense against persistent UFBs, we depleted CHMP7 to impair CHMP1B localization to the reforming NE (Fig. 5a). CHMP7 depletion fully recapitulated the phenotype observed in CHMP1B-depleted cells, as nearly all cell divisions regressed when LAP2B bridges induced by ICRF-193 were visible, whereas most cell divisions lacking these bridges completed cytokinesis without failure (Fig. 6g). Together, our data indicate that CHMP7-dependent CHMP1B localization to the reforming NE is required for CHMP1B association with UFBs, sustained NoCut activation and protection against tetraploidization. We note that depletion of either CHMP1B or CHMP7 did not impact the frequency of LAP2B bridges during cell division (Fig. 6g), supporting the specific role of ESCRT-III in determining the fate of unresolved UFBs but not their formation.

We then sought to evaluate whether stable expression of CHMP1B-L–GFP could rescue this regression phenotype, as this construct is resistant to CHMP1B siRNA. To investigate this, we engineered HeLa^mCh–LAP2B^ and HeLa^mCh–LAP2B/CHMP1B-L–GFP^ cells and performed binucleation experiments as described above. As a control, ICRF-193 induced regression rates of ~30% in both cells lines treated with control siRNA and depletion of CHMP1B in HeLa^mCh–LAP2B^ dramatically increased regression of mCh–LAP2B bridges to around 80% (Extended Data Fig. 9e). However, stable expression of CHMP1B-L–GFP followed by CHMP1B depletion reduced the regression rate to 50% (Extended Data Fig. 9e). This partial rescue is likely because of the subphysiological levels of the CHMP1B-L–GFP construct (Extended Data Fig. 1b). Importantly, this regression phenotype was observed only in cell divisions with mCh–LAP2B bridges, as cells lacking these bridges did not succumb to binucleation (Extended Data Fig. 9e). Thus, the CHMP1B-L–GFP construct is functional and the observed CHMP1B protection against binucleation is specific and rescuable.

### N-terminal truncation of CHMP1B interferes with NoCut protection against UFBs

**Fig. 7:**
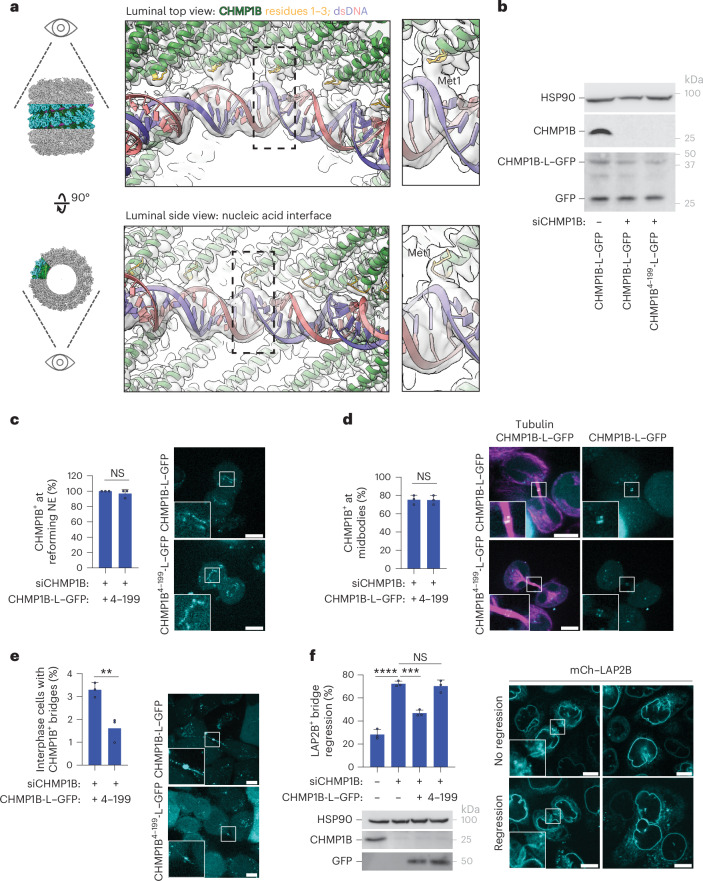
CHMP1B^4–199^ localizes less well to bridges and does not protect against binucleation associated with NoCut activation. **a**, The 3D reconstructions of dsDNA-templated CHMP1B–IST1 filament viewed down the helix axis (top) or as a slice through the side view (bottom). CHMP1B (green; three N-terminal residues highlighted in yellow) forms the inner strand and IST1 (cyan) forms the outer strand. The structures show CHMP1B–DNA contacts revealed in the density from a signal subtracted classification of the DNA density (class 9 from Extended Data Fig. 6). The dashed boxes zoom in on the dsDNA–Met1 interaction. **b**, Western blots of analysis HeLa cells stably expressing wild-type (WT) CHMP1B-L–GFP (transfected with siNT or siCHMP1B) and CHMP1B^4–199^-L–GFP (transfected with siCHMP1B) using the indicated antibodies. **c**, CHMP1B^4–199^ retains localization to the reforming NE. HeLa^CHMP1B-L–GFP^ (*n* = 22) and HeLa^CHMP1B (4–199)-L–GFP^ (*n* = 20) cells were transfected with siCHMP1B to deplete endogenous CHMP1B. Cells were imaged by live-cell microscopy through cell division. Localization of the exogenous CHMP1B constructs (cyan) to the reforming NE was quantified. **d**, CHMP1B^4–199^ retains localization to the midbody. HeLa^CHMP1B-L–GFP^ (*n* = 61) and HeLa^CHMP1B(4–199)-L–GFP^ (*n* = 60) cells were transfected with siCHMP1B to deplete endogenous CHMP1B. Cells were incubated with SiR–tubulin (magenta) and imaged by live-cell microscopy through cell division. Localization of the exogenous CHMP1B constructs (cyan) to midbodies was quantified. **e**, CHMP1B^4–199^ forms bridges less readily compared to CHMP1B^1–199^ following ICRF-193 treatment. HeLa^CHMP1B-L–GFP^ (*n* = 420) and HeLa^CHMP1B(4–199)-L–GFP^ (*n* = 309) cells were transfected with siCHMP1B to deplete endogenous CHMP1B. Cells were treated with 80 nM ICRF-193 for 24 h and then imaged. Quantification of CHMP1B-positive (cyan) bridges in interphase cells. **f**, CHMP1B^4–199^ fails to rescue binucleation of cells depleted of endogenous CHMP1B and treated with ICRF-193. HeLa^mCh–LAP2B^ cells transfected with siNT (*n* = 50), HeLa^mCh–LAP2B^ cells transfected with siCHMP1B (*n* = 32), HeLa^CHMP1B-L–GFP mCh–LAP2B^ cells transfected with CHMP1B (*n* = 39) and HeLa^CHMP1B(4–199)-L–GFP mCh–LAP2B^ cells transfected with siCHMP1B (*n* = 35) were treated with 80 nM ICRF-193 for 1 h and then imaged every 10 min for 48 h using live-cell microscopy. mCh–LAP2B (cyan) was tracked through each cell division and the proportion of regressions were quantified. Western blot analysis with anti-HSP90, anti-CHMP1B and anti-GFP are also shown. Representative images in **c**–**f** are from three independent experiments. Scale bars, 10 µm (**c**–**f**). Statistical analysis was conducted using an unpaired two-tailed *t*-test (**c**–**e**) or Tukey’s multiple-comparison test (**f**). Error bars in **c**–**f** represent the mean ± s.d.; independent experiments are plotted as dots. *P ≤ 0.05, **P ≤ 0.01, ***P ≤ 0.001 and ****P ≤ 0.0001. Source data

In our structures of CHMP1B–IST–DNA complexes, the N terminus of CHMP1B contacts the phosphate backbone (Fig. 7a and Extended Data Fig. 6d,f). To investigate the phenotypic consequences of removing this contact, we generated HeLa cells that stably expressed an N-terminal truncated CHMP1B^4–199^-L–GFP protein. This truncated construct, like its paired full-length wild-type CHMP1B-L–GFP control, was resistant to treatment with CHMP1B siRNA, allowing us to test its impact without interference from the endogenous protein. Both constructs were expressed at similar, subphysiological levels (Fig. 7b and Extended Data Fig. 1b). Importantly, both the full-length and the truncated CHMP1B constructs localized similarly to the reforming NE (Fig. 7c) and the midbody during cytokinesis (Fig. 7d). However, localization of the CHMP1B^4–199^ construct to DNA bridges induced by ICRF-193 was selectively reduced, indicating that the first three amino acids of CHMP1B are important for localizing CHMP1B to UFBs (Fig. 7e).

We also determined whether the truncated CHMP1B^4–199^ construct could rescue the regression phenotype induced by loss of endogenous CHMP1B and ICRF-193 treatment, like the full-length construct (Extended Data Fig. 9e). To do this, we also established HeLa cells that stably coexpressed mCh–LAP2B and CHMP1B-L–GFP or CHMP1B^4–199^-L–GFP. As expected, ICRF-193 treatment induced midbody regression in ~25% of cell divisions in control cells that contained endogenous CHMP1B and this level increased to almost 80% upon depletion of CHMP1B (Fig. 7f). Re-expression of wild-type CHMP1B-L–GFP partially rescued the enhanced regression phenotype (to ~45% regression), whereas reexpression of CHMP1B^4–199^-L–GFP completely failed to rescue the phenotype (Fig. 7f). Thus, the N-terminal region of CHMP1B, which contacts DNA in our structures, is required for efficient formation of CHMP1B-positive UFBs and NoCut protection against binucleation in cells.

## Discussion

We demonstrate here that the ESCRT-III subunit CHMP1B responds to and governs the NoCut mechanism that senses unresolved UFBs during mitotic closure of the NE and that IST1 and CHMP1B subunits transfer from the reforming NE onto mis-segregated UFBs. Persistent UFB coats also have an extended external NE membrane, as evidenced by the presence of the integral INM proteins LEM2 and emerin. Proper organization of this multilayered structure helps protect UFB-containing cells against tetraploidy by avoiding cleavage furrow regression during the early stages of cytokinesis, as evidenced by our observation that furrow regression increases dramatically in the absence of CHMP7 or CHMP1B (Figs. 6g and 7f and Extended Data Fig. 9e). The UFB structures coated with a multilayered membrane and ESCRTs also persist beyond cytokinesis and, as discussed below, we propose that they also protect unresolved, intercellular UFBs.

We provide multiple lines of evidence that ESCRT-positive internuclear bridges and NoCut functions are intimately linked. The selective requirement for CHMP1B to sustain NoCut in response to UFBs is consistent with a role for CHMP1B in sensing mis-segregated DNA at the reforming NE. Moreover, incorporation of CHMP4C into the persistent bridges suggests that CHMP1B–DNA complexes sustain NoCut activity. Conversely, prolonged association of CHMP1B with persistent UFBs is promoted by at least two NoCut components, namely CHMP4C and ULK3. Given that CHMP1B, IST1 and CHMP4C are all substrates of the ULK3 kinase^44^, phosphorylation of these ESCRT-III subunits may regulate their association with mis-segregated DNA. NoCut kinases could similarly regulate displacement of tight binding RPA subunits from unresolved UFBs in coordination with ESCRT coating. Interplay between DNA-associated factors and NoCut is further supported by recent work suggesting that topoisomerase IIa participates in NoCut sensing of chromatin bridges^75^, although it remains to be seen whether this mechanism also senses unresolved UFBs. The functional connection between UFB-associated factors and NoCut is further supported by recent work showing that BLM influences the activation of NoCut in response to unresolved UFBs^76^.

We showed that IST1 and CHMP1B can copolymerize into double-stranded protein filaments that encase and protect dsDNA, both in cells and in a pure recombinant system. This observation raises the possibility that CHMP1B may bind directly to the DNA component of UFBs. Consistent with this model, removal of an N-terminal CHMP1B–DNA contact observed in the recombinant CHMP1B–IST1–DNA structure reduces both UFB localization and protection against UFB-induced midbody regression and tetraploidy. We note, however, that the CHMP1B–DNA contact was not defined in molecular detail because our structures were not determined at atomic resolution, in part because the dsDNA does not follow the helical symmetry of the protein coat and because the CHMP1B N terminus is predicted to lack the N-terminal methionine residue and to be *N*-acetylated in human cells^77^. Although we cannot formally rule out the possibility that N-terminal truncation could alter protein–protein or protein–membrane interactions, these pleiotropic effects seem unlikely as the truncated CHMP1B^4–199^ protein localizes normally to the central midbody and to the reforming nuclear membrane.

**Extended Data Fig. 10:**
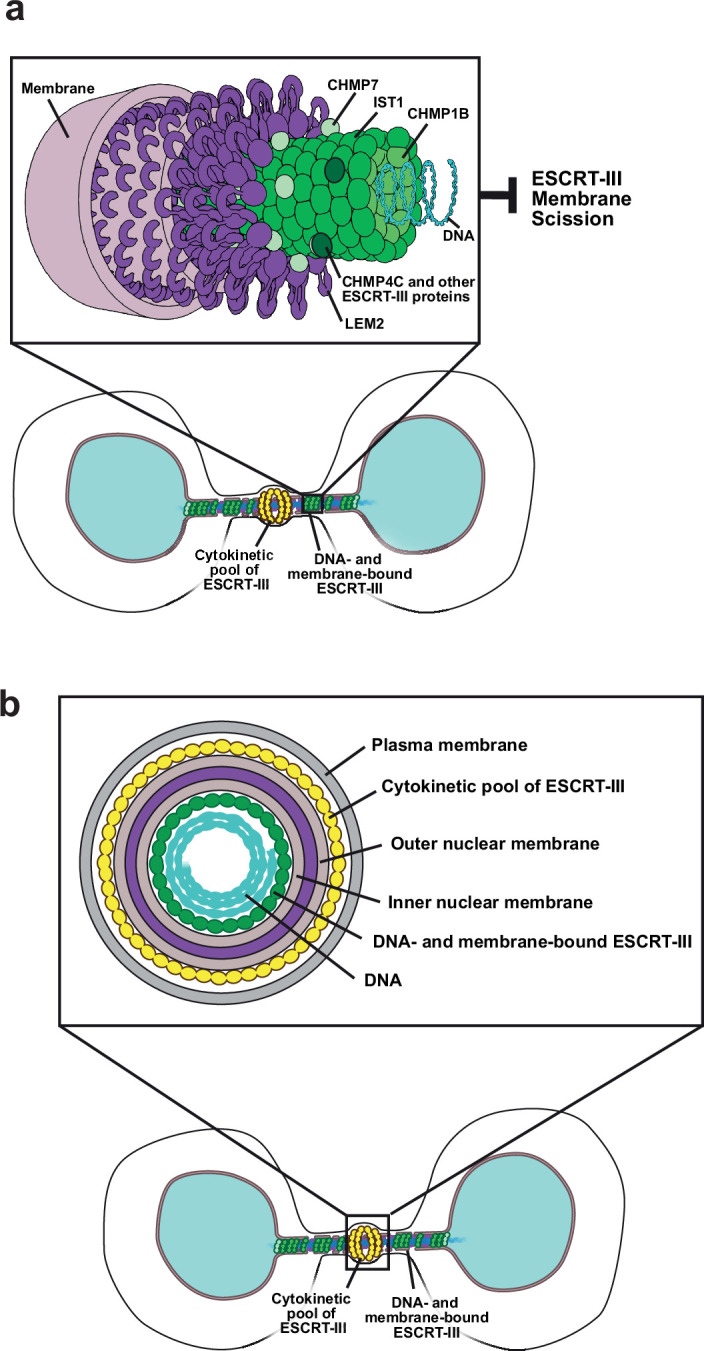
Models of ESCRT-III assemblies around persistent UFBs. **a**, Coaxial cable model for the ESCRT-III protective assemblies formed around UFBs. The upper panel emphasizes the multi-layered nature of the protective assemblies in which a central UFB is coated by CHMP1B/IST1 filaments and surrounded by other ESCRT-III proteins, all within a double bilayer derived from the NE. The lower figure illustrates that the ‘cytokinetic’ midbody and the ‘UFB’ (DNA/extended NE-associated) pools of ESCRT-III are distinct. **b**, Cross-section view of the coaxial cable model for the protective assemblies formed around UFBs. Cross-section view of the model in part a, emphasizing the multi-layered nature of the protective assemblies, the proximity of the ESCRT-III cytokinetic pool to the plasma membrane and NE, and that the UFB assembly resides within the distinct NE-derived midbody pool of ESCRT-III proteins.

Further studies are required to define the precise molecular organization of DNA, proteins and membranes at UFBs. One possibility is that they may resemble aberrant reforming nuclear membranes in which the membrane-associated ESCRT-III components are not released because the structure never closes because of the presence of a UFB. Alternatively, our data are also consistent with a ‘coaxial cable’ model for the organization of UFB assemblies in which CHMP1B binds directly to DNA (Extended Data Fig. 10a,b). In this model, DNA runs through the center of the cable and links the daughter nuclei, obstructing their sealing. The next layer of the cable comprises the CHMP1B–IST1 copolymer, which binds and wraps the central DNA. Additional layers of copolymerizing strands likely comprise other ESCRT-III proteins, including CHMP7, CHMP4B and CHMP4C, among others, which might contact the inner membrane. Lastly, the outermost wrapping of the cable is a LEM2-containing double bilayer that is contiguous with the NE.

The coaxial cable model has several attractive features. First, wrapped CHMP1B–DNA copolymers could protect persistent UFBs in interphase to prevent their catastrophic breakage by mechanical forces such as those imposed by cell migration^13^. Naked DNA is fragile and ruptures under forces of just 1–2 nN (ref. ^78^). However, the coiling of DNA within an ESCRT protein coat could dramatically buffer DNA against breakage, as evidenced by the fact that ~100-nN forces are required to break DNA in the context of chromatin^79^. ESCRT-III wrapping could also protect UFBs from recognition by detrimental proteins, including damaging nucleases such as TREX1 (ref. ^15^) and cytoplasmic innate immune DNA sensors such as cGAS^80^. Consistent with this idea, we found that CHMP1B–IST1 filaments protect DNA against both nuclease digestion and cGAS sensing in vitro (Fig. 4b and Extended Data Fig. 4b).

Interestingly, it was recently reported that ESCRT-III from the archaeal Asgard superphylum can also bind and protect DNA and chromatin^81,82^, supporting the idea that ESCRT-III nucleic acid binding is evolutionarily conserved. As cell division may be the primordial role of the ESCRT pathway^83,84^, it is noteworthy that the pathway has now been shown to participate directly in three fundamental aspects of cytokinesis: closing the reforming NE (in eukaryotes), separating the daughter cell cytoplasm through plasma membrane abscission and now sensing and protecting mis-segregated DNA that is aberrantly present during the mitotic reformation of the NE.

## Methods

### Plasmid construction

Coding sequences were amplified by PCR or generated by gene synthesis (Azenta) and inserted into the bicistronic retroviral packaging vectors pCMS28 or pNG72, as described previously^35^. A 25-nm linker followed by GFP was fused C-terminally to CHMP1B, CHMP4B or CHMP4C and the proteins were expressed at subphysiological levels, as described previously^28^. CHMP1B and IST1 expression constructs were generated by gene synthesis (Genscript) and inserted into pET28 *Escherichia* *coli* expression plasmids with N-terminal 6×His–SUMO tags^85^. Full details of plasmids and their sources are contained in the Supplementary Information.

### Cell culture

Female HeLa (CRM-CCL-2, American Type Culture Collection (ATCC)), 293T (CRL-3216, ATCC) and hTERT RPE-1 (CRL-4000, ATCC) cells were cultured and maintained in DMEM (Gibco) supplemented with 10% FBS (Sigma-Aldrich) and 20 µg ml^−1^ gentamicin (Life Technologies). To generate cell lines stably expressing proteins of interest tagged with monomeric fluorescent eGFP, YFP or mCh, 293T cells were transfected with retroviral packaging vectors, MLV-Gag-pol and pHIT VSV-G using polyethylenimine (Polysciences). After 48 h, the supernatant was harvested through a 0.22-µm Millex PVDF sterile filter and used to transduce HeLa cell lines. Cells were treated with 8 µg ml^−1^ polybrene (Merck) 15 min before transduction. Antibiotic selection was carried out 24 h later using 200 ng ml^−1^ puromycin (Sigma-Aldrich) or 500 µg ml^−1^ G418 (Invitrogen).

To generate HeLa^CHMP1B-L–HaloTag^ stable cell lines with doxycycline-inducible expression, HeLa cells were transduced with pLVX-TetOne CHMP1B-L–HaloTag lentiviral vector (Takara) and selected for 5 days with 1 µg ml^−1^ puromycin. Monoclonal populations were isolated by limiting dilution and tested for CHMP1B-L–HaloTag expression by live-cell imaging and western blotting following addition of 0.2 µg ml^−1^ doxycycline for 18–24 h.

Cell lines were regularly tested for the presence of *Mycoplasma* using MycoAlert (Lonza).

### siRNA transfections

Cells were transfected with siRNA for 48 h using Lipofectamine RNAiMax (Thermo Fisher) according to manufacturers’ instructions for the reverse transfection procedure. The siRNA oligonucleotides are listed in the Supplementary Information.

### Immunofluorescence imaging

Cells were grown on coverslips, washed once in PBS (Gibco) and fixed for 10–20 min in 4% PFA (EM Sciences) at room temperature or overnight in methanol at −20 °C. Cells were blocked with 3% BSA (Sigma-Aldrich) and 0.1% Triton X-100 (Sigma-Aldrich) in PBS for 1 h. Primary antibodies were applied for at least 1 h. After washing three times with PBS, secondary antibodies were applied for 1 h and nuclei were stained with DAPI, Hoechst 33342 (Sigma-Aldrich) or SPY555-DNA probes (Spirochrome). Coverslips were mounted with ProLong Gold Antifade Reagent (Invitrogen) on microscope slides or directly into wells of 24-well imaging plates (Eppendorf). Images were acquired using a Nikon Ti-Eclipse wide-field inverted microscope, Eclipse Ti-E inverted CSU-X1 spinning-disk confocal microscope or ECLIPSE Ti2 inverted AX/AX R confocal microscope system. Scale bars are 10 µm unless otherwise stated.

### Immunoblotting

Cell lysates were denatured in Laemmli buffer (Bio-Rad), resolved by SDS–PAGE and transferred to 0.2-µm nitrocellulose membranes (GE Healthcare). Membranes were blocked with 1% milk in 0.1% Tween-20 in Tris-buffered saline (TBS) and incubated with primary antibodies in blocking solution for 3 h at room temperature or overnight at 4 °C. Membranes were washed in 0.1% Tween-20 in TBS and incubated with the corresponding secondary antibodies conjugated with horseradish peroxidase (HRP) or near-Infrared fluorescent dyes in blocking solution for 1 h at room temperature. For HRP-conjugated secondary antibodies, membranes were further treated with Amersham ECL Prime western blotting detection reagent (GE Healthcare). Proteins were detected and quantified using a Li-Cor Odyssey Infrared scanner and software (Li-Cor Biosciences) or Image Quant LAS 400 (GE Healthcare). Antibodies and dilutions are listed in the Supplementary Information.

### Live-cell microscopy

#### Wide-field microscopy

HeLa YFP–LAP2B cells were plated in wells of a 24-well glass bottom imaging plate (Eppendorf), transfected with siRNA and incubated with DMSO, ICRF-193 or probes before imaging (as indicated in the figure legends). Cells were imaged for 24 h, on a Nikon Ti-Eclipse wide-field inverted microscope (Nikon ×40 dry objective lens) equipped with a Perfect Focus system and housed in a 37 °C chamber (Solent Scientific) fed with 5% CO_2_. Multiple fields of view were selected at various *xy* coordinates; three *z* slices were captured at a 0.96-μm spacing. Images were acquired at indicated time intervals using a Hamamatsu Orca Flash 4.0 camera (Hamamatsu Photonics), controlled by NIS-Elements software.

HeLa^LEM2–mCh/CHMP1B-L–GFP^ cells were seeded in no. 1.5H ibidi dishes coated with poly(d-lysine) and treated with 100 nM ICRF-193 or DMSO for 18 h. Cells were washed once with PBS before addition of prewarmed DMEM without phenol red before the addition of Prolong Live (1:200). Metaphase cells were picked and wide-field images were acquired using a Nikon Ti2-E microscope equipped with a Lumencor Sola SE U-NIR, MCL Piezo automated stage, Nikon Perfect Focus system and Prime 95b 25-mm scientific complementary metal–oxide–semiconductor (sCMOS) camera using a CFI PlanApo Lambda ×60/1.4 objective.

HeLa^CHMP1B-L–HaloTag^ cells were seeded in no. 1.5H ibidi dishes coated with fibronectin and treated with 100 nM ICRF-193 or DMSO for 18 h. Then, 200 nM JF635 HaloTag ligand and 200 ng ml^−1^ Hoechst were added 30 min before imaging. Images were acquired using a Nikon Ti-E inverted microscope equipped with a ×60 PlanApo oil-immersion objective and an Andor Zyla CMOS camera.

#### Spinning-disk confocal microscopy

HeLa mCh–RPA3, CHMP1B-L–GFP, CHMP4C-L–GFP and CHMP4B-L–GFP cells were plated and treated as described. Cells were imaged at a single time point (interphase cells, 80–160 nM ICRF-193 treatments for 18–24 h before imaging) or continuously until asynchronous cells had reached cytokinesis (mitotic cells, 80 nM ICRF-193 treatments for 1 h before imaging). A Nikon Eclipse Ti-E Inverted CSU-X1 spinning-disk confocal microscope (Nikon ×60 oil objective lens) equipped with an Andor Zyla sCMOS was used.

For experiments involving DNA foci, cells were transfected with 500 ng of pCR3.1 using the TransIT-LT1 transfection reagent (Mirus) according to the manufacturer’s instructions. TransIT-LT1:DNA complexes were preincubated for 30 min and added to cells. Cells were then incubated with SPY555-DNA or SPY595-DNA to visualize the DNA foci. Imaging was initiated 4 h after transfection.

EdU experiments used the Click-iT EdU Alexa 555 imaging kit (Thermo Fisher). Cells were treated with 80 nM ICRF-193 and 10 µM EdU for 24 h, fixed and stained following the manufacturer’s instructions and then imaged (relevant details in the figure legends).

### SIM

LEM2–mCh CHMP1B-L–GFP HeLa cells were washed once with PBS and fixed with 4% PFA for 15 min (all steps at 23 °C), before being blocked and permeabilized with 0.05% Triton X-100 and 3% normal goat serum in PBS for 1 h. Coverslips were incubated with primary antibodies overnight (GFP: AbCam, ab1218; mCh: AbCam, ab167453), washed three times with PBS and incubated with secondary antibodies (Thermo Fisher, A-11001) for 1 h. Coverslips were washed three times with PBS and stained with Hoechst reagent for 30 min. Coverslips were washed again three times with PBS and mounted with vectashield. Cells were imaged on a GE OMX-SR inverted microscope with three PCO 15-bit CMOS cameras and Toptica four-line laser launch using a Plan ApoN ×60/1.42 oil objective and immersion oil (refractive index: 1.516). Images were acquired using GE Acquire SR software in 3D structure illumination mode, then reconstructed and aligned using GE Softworx. The quality and reliability of structured illumination reconstructions were assessed using the SIMCheck Fiji plugin.

### Light microscopy image analysis and figure assembly

Wide-field, confocal and structured illumination images were analyzed in ImageJ (Fiji). To measure interphase bridge frequency, total numbers of dual-color-labeled interphase cells and interphase bridges were counted using the Cell Counter analysis plugin. Characteristic images were adjusted to minimize background and visualize features of interest. Data were graphed in GraphPad Prism 9. Error bars shown indicate the mean ± s.d. and all experiments represent at least 3 biological replicates unless otherwise stated. ImageJ was used to export final microscopy images into Illustrator (Adobe) for assembly. Photoshop (Adobe) was used to assemble videos.

### Purification of ESCRT-III proteins

#### CHMP1B

Human codon-optimized 6×His–SUMO CHMP1B-Met136Val pET28a(+) plasmid was transformed into BL21-Codon Plus (DE3) RIPL cells (Agilent). Cells were grown in Luria–Bertani medium at 37 °C to an optical density at 600 nm of 0.6–0.8, protein expression was induced with 1 mM IPTG and the temperature was shifted to 19 °C for 12–16 h. Cells were harvested and frozen at −80 °C. All subsequent steps and pH measurements were performed at 4 °C unless otherwise noted. Thawed cells were suspended in lysis buffer (50 mM Tris pH 8 at 4 °C, 300 mM NaCl, 10 mM imidazole, 1 mM DTT and 0.5 mM EDTA), supplemented with lysozyme (0.2 mg ml^−1^), protease inhibitors (PMSF, pepstatin, aprotinin and leupeptin), DNase I (Roche) and 2 mM MgSO_4_. Each gram of cell pellet was suspended in 5 ml of lysis buffer and cells were ruptured by sonication with a tip sonicator (10 s on–off cycles at 50% amplitude, 5 min in total), filtered through a 100-μm nylon filter (Falcon) and emulsified using an Emulsiflex C3 homogenizer (Avestine). The lysate was further processed with the addition of 0.125% (w/v) sodium deoxycholate and centrifuged at 35,000*g* for 1 h; the supernatant was filtered through a 0.45-µm syringe filter. Clarified lysate was loaded onto a Cytiva His-Trap HP 5-ml affinity column on an Akta Pure fast protein liquid chromatography (LC) instrument and washed with five column volumes of affinity buffer A (50 mM Tris pH 8, 300 mM NaCl, 10 mM imidazole and 1 mM DTT). The fusion protein was eluted with an isocratic step to 35% affinity buffer B (50 mM Tris pH 8, 300 mM NaCl, 500 mM imidazole and 1 mM DTT) to ~112 mM imidazole and dialyzed in Slide-A-Lyzer G3 dialysis cassettes (12.5-kDa molecular weight cutoff (MWCO); Thermo Scientific) with His-tagged ULP1 protease (0.75 mg) to cleave the His–SUMO tag during dialysis (50 mM Tris pH 8.0, 150 mM NaCl and 1 mM DTT). Solution containing the cleaved fusion protein was applied to fresh cOmplete His-Tag purification resin (Roche) to remove the His–SUMO tag and His-tagged ULP1 (cleaved CHMP1B does not bind the cOmplete resin). CHMP1B was purified to homogeneity by Superdex-75 size-exclusion chromatography in CHMP1B SEC buffer (50 mM Tris pH 8.0, 300 mM KCl, 1 mM DTT and 5% glycerol (v/v); (Cytiva). Typical protein yields were 1–3 mg l^−1^ culture. The CHMP1B-Met136Val identity was confirmed by mass spectrometry (MS; Mr_Calc_ = 22,076.3 Da, Mr_Exp_ = 22,076.2 Da).

#### IST1

A human codon-optimized 6×His–SUMO IST1 pET28c(+) plasmid was used for expression and purification of full-length IST1. The same method for purifying CHMP1B was used to purify IST1 with the following deviations. Lysis buffer (1 M KCl, 50 mM HEPES pH 7.5, 20 mM imidazole, 5 mM β-mercaptoethanol (BME) and 5% glycerol (v/v)) supplemented with 0.125% sodium deoxycholate (w/v), lysozyme (0.2 mg ml^−1^), protease inhibitors (PMSF, pepstatin, aprotinin and leupeptin) and DNase I (Roche). His-trap wash buffer (1 M KCl, 50 mM HEPES pH 8.0, 20 mM Imidazole, 5 mM BME and 5% glycerol (v/v)) and elution buffer (1 M KCl, 50 mM HEPES pH 7.5, 750 mM imidazole, 5 mM BME and 5% glycerol (v/v)) were used for affinity purification (35% isocratic elution step, ~275 mM imidazole), followed by dialysis in a Slide-A-Lyzer G3 dialysis cassettes (12.5-kDa MWCO; Thermo Scientific) with His-tagged ULP1 protease (0.75 mg) and equilibration into dialysis buffer (50 mM Tris pH 8.0, 150 mM NaCl and 1 mM DTT). The cleaved fusion protein solution was applied to fresh cOmplete His-Tag purification resin (Roche) to remove the His–SUMO tag and His-tagged ULP1 (cleaved IST1 does not bind the resin). IST1 was purified by Superdex-75 size-exclusion chromatography in IST1 SEC buffer (50 mM HEPES pH 8.0, 300 mM KCl, 5 mM BME and 5% glycerol (v/v); Cytiva). Typical protein yields were ~30 mg l^−1^ culture and the IST1 identity was confirmed by MS (Mr_Calc_ = 39,749.5 Da, Mr_Exp_ = 39,749.4 Da).

#### IST1_NTD_ (residues 1–189) and IST1_NTD_-Tyr165Ala

IST1_NTD_ and IST1_NTD_-Tyr165Ala were purified as described previously for IST1_NTD_^74^. Yields for IST1_NTD_-Tyr165Ala were 5 mg l^−1^ culture and the IST1_NTD_-Tyr165Ala identity was confirmed by MS (Mr_Calc_ = 21,643.67 Da, Mr_Exp_ = 21,643.67 Da).

### Intact protein MS

Reversed-phase LC–MS was performed using LC–MS-grade acetonitrile, formic acid, Tris-HCl and DTT (Burdick & Jackson, Honeywell; Sigma-Aldrich) on an Eksigent Ekspert nanoLC 425 system (SciEx) coupled to a Bruker MAXIS ETD II quadrupole time-of-flight MS instrument. Samples were diluted 1:1 with 0.1% formic acid in water. Then, 5-µl samples were separated on reverse-phase gradients (buffer A: 0.2% formic acid in water; buffer B: 0.2% formic acid in acetonitrile) at a flow rate of 10 µl min^−1^ at 60 °C for 56 min (5% buffer B to 95% B over 35 min) on a 10-cm (inner diameter: 1 mm) Waters Acquity ultrahigh-performance LC BEH column. Data analysis was performed using Bruker Compass DataAnalysis (version 5.2) software. Deconvolution was performed using maximum entropy with the instrument resolving power at 80,000 and high resolution.

### C-terminal ESCRT-III peptides

ESCRT-III C-terminal peptides were expressed as His–SUMO fusion proteins (except for synthetic CHMP1B_169__–199_, CHMP1A_182__–196_, CHMP1B_183__–199_ and CHMP2B_196__–213_, made by the University of Utah DNA/Peptide Synthesis Core) in 2-l cultures of BL21-Codon Plus (DE3) RIPL cells (Agilent) in ZYP-5052 autoinduction medium^86^ and purified at 4 °C. Expressing cells were lysed by sonication (40 ml l^−1^ of culture in 50 mM Tris pH 7.2, 150 mM NaCl, 5 mM imidazole, 2 mM DTT, 0.5 mM EDTA and 0.125% sodium deoxycholate, supplemented with lysozyme, protease inhibitors and DNase I (Roche)). Clarified lysates were incubated with 10 ml of cOmplete His-Tag purification resin (Roche) for 20 min, washed with ten column volumes of wash buffer (50 mM Tris pH 7.2, 500 mM NaCl, 5 mM imidazole, 5 mM DTT and 0.5 mM EDTA) followed by ten column volumes of wash buffer containing 150 mM NaCl. The His–SUMO affinity tag was removed from resin-bound peptides by overnight incubation with ULP1 protease (0.7 mg) in 40 ml of the 150 mM NaCl wash buffer. Cleaved peptides were collected from the column flowthrough and dialyzed against Q-Sepharose-binding buffer (25 mM sodium phosphate pH 6.5, 50 mM NaCl, 2 mM DTT and 0.5 mM EDTA) and further purified by Q-Sepharose anion-exchange chromatography (Cytiva) with a linear gradient from 50 mM to 1 M NaCl. Fractions containing peptides were pooled and dialyzed against gel filtration buffer (25 mM Tris pH 7.2, 150 mM NaCl, 1 mM TCEP and 0.5 mM EDTA) and purified by Superdex-75 size-exclusion chromatography (Cytiva). Typical peptide yields were 4.5 mg l^−1^ culture (IST1). Purified ESCRT-III C-terminal fragments contain nonnative ‘GC’ residues at their N termini and masses were confirmed either before labeling (CHMP4C, CHMP1A_140__–196_ and CHMP1B_143__–199_) or after fluorescence labeling (all other peptides; +dye yields a mass shift of 463.4 Da) by MS (Supplementary Information).

Fluorescence labeling was performed by the University of Utah DNA/Peptide Synthesis Core as described previously^44^. Briefly, peptides were labeled in DMSO at 4 °C with approximately 1.3-fold molar excess of Oregon Green 488 maleimide (Life Technologies/Molecular Probes 06,034) dissolved in a 1:1 solution of acetonitrile–DMSO. Reaction progress was monitored by HPLC and labeled peptides were separated from free dye and residual unlabeled peptides using the reversed-phase LC conditions described above. Peptide fractions were dried under vacuum and redissolved in water; concentrations were calculated using the absorbance of Oregon Green 488 at 491 nm (Extinction coefficient 83,000 cm^−1^ M^−1^ in 50 mM potassium phosphate, pH 9).

### Plastic-embedded thin-sectioned TEM

HeLa cells were seeded as monolayers in six-well cell culture plates and grown to 80% confluency. The medium (Gibco DMEM) was replaced with fresh medium before cotransfection with mammalian expression vectors for IST1 and CHMP1B (pCAG-MYC-IST1 (1.5 µg) and pCAG-CHMP1B-FOS (1.5 µg)) using Lipofectamine 2000. Cells were harvested 24 h after transfection using a rubber cell scraper and incubated with cold fixative solution (2.5% glutaraldehyde, 1% PFA and 0.1 M cacodylate buffer pH 7.2) for 30 min.

Pelleted cells were gently rinsed three times with cacodylate buffer and postfixed with buffered 2% osmium tetroxide solution for 1 h at room temperature, rinsed with cacodylate buffer and distilled H_2_O, prestained for 1 h with saturated filtered uranyl acetate and dehydrated using an ethanol-graded series for 5 min each (50%, 70%, 95%, 95%, 100%, 100%, 100%) followed by pure acetone (three times for 5 min). Pelleted cells were then infiltrated with 50% epon resin and acetone for 1 h, 75% epon resin overnight and 100% epon resin over 8 h with three changes. Cells were embedded in polymerized resin at 60 °C for 24 h.

Ultrathin (70 nm) sections were cut with a diamond knife (Diatome) and an ultratome Leica UC6 (Leica Microsystems). Grids with sections were poststained with saturated uranyl acetate in distilled H_2_O for 20 min and with lead citrate for 10 min and imaged at 120 kV using a JEOL-JEM 1400 Plus EM instrument.

### Production and extraction of CHMP1B–IST1 filaments from cells

HeLa cells (CRM-CCL-2, ATCC), cultured and maintained in DMEM (Gibco) supplemented with 10% FBS (Sigma-Aldrich) were seeded as monolayers on 10-cm plates and grown to 80% confluency. The medium was replaced with fresh medium and cotransfected with mammalian expression vectors for IST1 and CHMP1B (pCAG-MYC-IST1 (6 µg) and pCAG-CHMP1B-FOS (6 µg)) using Lipofectamine 2000. Cells were harvested 24 h after transfection with a rubber cell scraper and resuspended in 1 ml of PBS. Resuspended cells were lysed by dounce homogenization in a 2-ml glass dounce (50×) and the resulting lysate clarified by sequential differential centrifugation at 300*g* (5 min) and 5,000*g* (5 min) at 4 °C to remove membrane-bound fractions. Clarified lysate was further spun at 30,000*g* (1 h) at 4 °C (Beckman L8-55MR ultracentrifuge in a SW55Ti swinging bucket rotor). Pellets with CHMP1B–IST1 filaments were resuspended in ~40 μl of PBS and vitrified as described below.

### Nuclease protection assays

Nucleic-acid-nucleated CHMP1B–IST filaments were formed using 100-µl aliquots of 50 mM Tris pH 7.0, 350 mM NaCl, 5% glycerol, 5 mM β-mercaptoethanol and 5 µg of 60-mer synthetic dsDNA in the absence (control) or presence of 20 µM CHMP1B and IST1 (each) by overnight dialysis (23 °C, in 25 mM Tris pH 8.0 and 25 mM NaCl). dsDNA or dsDNA:protein complexes were then incubated at 23 °C with DNase I (New England Biolabs, M0303S) supplemented with 0.5 mM CaCl_2_ and 2.5 mM MgCl_2_ at a final concentration of 0.05 U per µg of DNA. Reactions were quenched (5, 10, 20, 30 and 60 min) by mixing with 10 mM EDTA, 10 mM EGTA and 150 mM NaCl in 200 µl of phenol–chloroform–isoamyl alcohol solution. Nucleic acids were recovered by centrifugation at 23 °C for 10 min at 16,000*g*, the aqueous phase was recovered, 100 µl of phenol–chloroform–isoamyl alcohol was added and the solution was centrifuged at 23 °C for a further 10 min at 16,000*g*. The resulting aqueous phase was treated with 10 µl of glycogen (5 mg ml^−1^ stock), 0.1 volumes of 3 M sodium acetate (pH 5.5) and 0.7 volumes of isopropyl alcohol, followed by centrifugation at 16,000*g* at 4 °C for 30 min. The supernatant was aspirated and the pellet was vortexed in 500 µl of ice cold 70% ethanol followed by centrifugation at 16,000*g* for 10 min. The supernatant was removed and the pellet was vortexed in 20 µl 10 mM Tris pH 7.4 and 50 mM NaCl, followed by incubation for 1 h at 37 °C and further vortexing. The entire 20 µl of each reaction was electrophoresed on a 1% agarose gel and stained with SyberSafe green dye (Invitrogen). Gel densitometry analysis was performed with ImageJ.

### cGAS sensing assay

CHMP1B–IST1 and dsDNA (20 μM CHMP1B, 20 μM IST1 and 79 ng of linearized pUC19 plasmid dsDNA) were coassembled by dialysis (12 h, 23 °C, 50 mM Tris-HCl pH 8.0, 125 mM NaCl and 1 mM DTT). Control assembly reactions lacked either CHMP1B–IST1 or dsDNA. Assembly reactions were then diluted 1:10 into cGAS assay reactions (50 mM Tris pH 7.5, 100 nM NaCl, 40 μM inositol hexakisphosphate, 1 mg ml^−1^ BSA, NTPs (1.124 mM ATP, 323 μM GTP, 173 μM UTP and 25 μM CTP) and 2.2 mM MgCl_2_)^87^ and serially diluted fourfold in assembly reaction buffer. To test for DNA activation and cGAMP production, the CHMP1B–IST1–dsDNA complexes and controls were incubated with full-length recombinant human cGAS (10 nM in 100-μl reaction volumes, 8 h, 37 °C). Completed reactions were held at 4 °C for short term storage or frozen at −80 °C. cGAMP levels in each sample were determined by ELISA (Arbor Assays) following the manufacturer’s instructions. Samples were diluted 2–100-fold in assay buffer to fall within the linear range of a standard curve (0.05–20 pmol ml^−1^ purified 2′3′-cGAMP) and the pUC19 dsDNA concentrations in each sample were double-checked and normalized by qPCR. Data were analyzed and plotted using GraphPad Prism software.

### CHMP1B–IST1–nucleic acid complexes for structure determination

#### Nucleic-acid-bound CHMP1B–IST1 filaments

First, 100-μl aliquots of 50 mM Tris pH 7.0, 300 mM NaCl, 5% glycerol and 5 mM β-mercaptoethanol containing 16 µM CHMP1B, 16 µM IST1 and 5.33 µM oligo (320 μM bases/base pairs) of the corresponding nucleic acid (1:20 protein:base/base pair molar ratio) were dialyzed overnight (23 °C, 25 mM Tris pH 8.0 and 125 mM NaCl). Assemblies were concentrated by centrifugation (2,152*g* for 10 min 23 °C). Next, 75 μl of the supernatant was removed and the pelleted filaments were resuspended with the remaining 25 μl, thereby concentrating the reaction fourfold.

#### Dinucleosome templated CHMP1B–IST1 filaments

Dinucleosome templates contained two recombinant histone octamers wrapped by a 442-bp dsDNA template with an intervening 60-bp dsDNA linker (Epicypher PN:16-3104). First, 100-μl aliquots of 25 µM CHMP1B, 15 µM IST1 and 0.25 µM dinucleosomes (1:100 dinucleosome:CHMP1B–IST1 dimer) in 50 mM Tris pH 7.0, 300 mM NaCl, 5% glycerol and 5 mM BME) were dialyzed against 125 mM NaCl and 25 mM Tris pH 8.0 (4 °C, 12 h). Large-molecular-weight assemblies were concentrated (2,152*g*, 10 min, 23 °C). Then, 25 μl of the supernatant was removed and the pelleted filaments were resuspended in the remaining 25 μl, thereby concentrating the reaction twofold.

### Sample vitrification

Cellular CHMP1B–IST1 filament solutions (3.5 µl) were applied for 1 min at 11 °C and 85% humidity to glow-discharged (10 s, 25 mA, PELCO easiGlow Glow Discharge Cleaning System) Lacey Carbon 300-mesh Cu grids (Ted Pella, 01895-F) in a GP2 plunge freezer (Leica). Grids were blotted with Whatman filter paper no. 1 for 7–11 s before plunge-freezing in liquid ethane.

Assembled recombinant CHMP1B–IST1 filament solutions (in nucleosome and bound to nucleic acid; 3.5 µl) were applied to glow-discharged (30 s, 25 mA, PELCO easiGlow Glow Discharge Cleaning System) 25-nm holey Au R0.6/1 Quantifoil 300-mesh grids (Quantifoil) in a Mark IV Vitrobot (Thermo Fisher Scientific). Grids were blotted with Whatman filter paper no. 1 for 4–6 s with a 0-mm offset at 19 °C and 100% humidity before plunge-freezing in liquid ethane. Grids were stored under liquid nitrogen until imaged.

### Cryo-EM data collection

Cryo-EM data for cellular, recombinant nucleic-acid-bound and nucleosome CHMP1B–IST1 filaments were acquired using automated TEM instruments. Cellular CHMP1B–IST1 filaments were imaged on a Titan Krios G3 (Thermo Fisher Scientific) cryo-TEM instrument operating at 300 keV and equipped with a post-GIF K3 direct electron detector (Gatan). Nucleic-acid-bound CHMP1B–IST1 filaments were imaged at 200 keV using a Glacios cryo-TEM equipped with a Selectris energy filter and a Falcon4i direct electron detector (Thermo Fisher Scientific). Nucleosome CHMP1B–IST1 filaments were imaged at 300 keV using a Titan Krios G4 cryo-TEM instrument equipped with a Selectris energy filter and a Falcon4i direct electron detector (Thermo Fisher Scientific). Data collection parameters are provided in Supplementary Table 5. Objective lens astigmatism and axial coma were minimized using automated tuning protocols before data acquisition. For EER-based datasets, frames were combined into discrete fractions (representing an exposure of approximately 1 e^−^ per Å^2^) and processed without upsampling.

### Cryo-EM reconstructions

#### Image processing and model generation workflow for cellular CHMP1B–IST1 filaments

The helical reconstruction pipeline within RELION (version 4.0-beta-2-commit-2fba16)^88^ was used to motion correct (MotionCor2)^89^ and estimate the contrast transfer function (CTF; CtfFind-4.1.14)^90^ for corrected micrographs from SerialEM and EPU datasets individually and to manually pick helical filaments after merging the two datasets. A total of 70,300 segments were extracted using a 342.8-Å box (648-pixel box for videos collected using SerialEM or 324-pixel box for videos collected using EPU; both binned into 288-pixel boxes). From the 65,700 segments retained after one cycle of two-dimensional (2D) classification (imposing particle alignment), a scarce 2D class with strong nucleic acid signal was identified (229 segments, 0.35%). The 229 segments were used to generate a 3D model (using a 250-Å-diameter featureless cylinder and imposing a 20° twist and 3 Å rise). A mask focused on the nucleic acid density within the tube lumen of this model was used in 3D classifications of a consensus helical model containing all 65,700 segments, resulting in two major populations, one of which showed strong internal nucleic acid density (10,115 segments). The bound nucleic acid was readily visible in the 3D Refine model (*C*_1_ symmetry) of the 10-K segment set and was missing in the 56-K set. Segments from both classes were imported to cryoSPARC (version 4.3.1)^91^ and each was further 3D-classified into three tube classes, (starting from a cylinder with inner diameter of 80 Å and outer diameter or 250 Å, allowing focused helical searches around a rise of 3.0 ± 1.0 Å and twist of 20° ± 3°). These three templates were used in a heterogeneous refinement step for each set of segments and the resulting classes and corresponding 3D helical models were further subjected to subsequent rounds of heterogeneous refinement and helical refinement. Reconstruction resolutions were estimated using global and local refinement protocols in cryoSPARC (version 4.3.1). This workflow is summarized in Extended Data Fig. 4 and refinement parameters are provided in Supplementary Table 1.

#### Image processing and model generation workflow for recombinant CHMP1B–IST1 filaments

Helical data processing through cryoSPARC (version 4.2.1)^91^ was used to process CHMP1B–IST1 filaments formed in vitro. Selected micrographs with optimal imaging parameters (CTF fit estimation, defocus range and estimated ice thickness) were manually picked to generate initial 2D classes for template-based autopicking. Particles were extracted into 357.4-Å (400-pixel) boxes and rounds of 2D classification executed to isolate filament classes with strong secondary-structure elements and remove false positives and intersecting particles. Consensus helical refinements were generated from featureless cylinder input models (outer diameter: 250 Å, inner diameter: 80 Å), allowing focused helical searches around a rise of 3.0 ± 0.4 Å and twist of 20° ± 3°. The 3D classifications then identified homogeneous subsets. Helical refinements were performed on all isolated classes to generate final maps for model building and analysis. The helical class with most data for each nucleic acid substrate was used for interpretation. The reconstruction resolutions for each nucleic acid substrate density maps were estimated by applying global and local refinement protocols in cryoSPARC (version 4.2.1). This workflow is summarized in Extended Data Fig. 4 and refinement and symmetry parameters are provided in Supplementary Table 2.

To generate coordinate models for the nucleic acid templated copolymer reconstructions, the membrane-bound CHMP1B (PDB 6TZ4) model and IST1 crystal structure (PDB 3FRR) were stripped of ligands and fit into the sharpened symmetrized density maps using ISOLDE (version 1.6), with default distance restraints generated from the idealized model^57,67^. Fitted coordinate models were finalized by refinement with Servalcat (version 0.4.39)^92^ (servalcat refine_spa_norefmac) with helical symmetry and geometry assessed by Molprobity (version 4.5.2)^93^. Models are deposited as asymmetric units with the helical copies stored in the BIOMTX records.

#### Focused classification of nucleic acid density from in vitro CHMP1B–IST1 copolymer filaments

Similar reconstruction procedures were performed for dsDNA, ssDNA and ssRNA datasets. From the consensus helical reconstruction, density corresponding to the protein coat was isolated using ChimeraX (version 1.6.1)^94^ and used as a template to generate a soft-edged mask for classification. The protein-encompassing mask was used along with the consensus reconstruction as inputs for partial signal subtraction in cryoSPARC (version 4.2.1) to remove protein signal. The alignment metadata were then symmetry-expanded on the basis of the helical parameters determined by the consensus helical refinement. A homogeneous reconstruction was performed on the subtracted, symmetry-expanded data and density corresponding to approximately half a rung of nucleic acid was isolated using ChimeraX (version 1.6.1) to use as a template for a soft-shaped mask used for classification. Classification without alignment was performed in cryoSPARC (version 4.2.1) on the subtracted, symmetry-expanded data with multiple (dsDNA, *k* = 10; ssDNA, *k* = 3 and 5; ssRNA, *k* = 10) classes, a high-resolution limit of 10 Å, force-hard classification = true and force redo Fourier shell correlation split = false.

To fit coordinate models into the dsDNA classes exhibiting major and minor grooves, an idealized 60-mer of poly(AT) was generated in Coot (version 0.9.8.1)^95^. The idealized 60-mer was then fitted into the density maps using ISOLDE (version 1.6)^96^ with default distance restraints generated from the idealized model. The fitted model was analyzed using ChimeraX (version 1.6.1) and Web 3DNA (https://x3dna.org/)^97^. This workflow is summarized in Extended Data Fig. 6 and refinement parameters are provided in Supplementary Table 2.

### Fluorescence polarization binding experiments

IST1_NTD_ fluorescence polarization binding experiments were performed in binding buffer (25 mM Tris pH 7.2, 300 mM NaCl, 0.1 mg ml^−1^ BSA, 0.01% Tween-20 and 1 mM DTT) with 5-10 nM fluor-labeled terminal ESCRT-III peptides (Supplementary Information) and the designated dilutions of IST1_NTD_ and IST1_NTD_-Tyr165Ala. Fluorescence polarization was measured using a Synergy Neo multimode plate reader (Biotek) with excitation at 485 nm and detection at 535 nm. Dissociation constants were calculated by fitting the increase in fluorescence polarization to a 1:1 binding equation using KaleidaGraph (Synergy Software) as described previously^98^. Each binding isotherm was measured at least three times independently and the mean ± s.d. Dissociation constant (*K*_d_) values are reported.

### Quantification and statistical analysis

All experiments and microscopy images are representative of at least three biological replicates. Error bars in quantifications are the mean ± s.d. unless otherwise stated. An unpaired Student’s *t*-test was used for the comparison of two groups. A Tukey post hoc test was used for multiple comparisons. A *P* value > 0.05 was considered not significant. *P* < 0.05 (*), *P* < 0.01 (**), *P* < 0.001 (***) and *P* < 0.0001 (****) were considered significant. Specific details of statistical analyses are included in the figure legends where relevant and in Supplementary Table 3.

### Reporting summary

Further information on research design is available in the Nature Portfolio Reporting Summary linked to this article.

## Online content

Any methods, additional references, Nature Portfolio reporting summaries, source data, extended data, supplementary information, acknowledgements, peer review information; details of author contributions and competing interests; and statements of data and code availability are available at 10.1038/s41594-026-01841-4.

## Supplementary information

Supplementary Information Supplementary Fig. 1 (and figure caption), Supplementary Tables 1, 2 and 5 and Resource Table.

Reporting Summary

Supplementary Video 1 CHMP1B replaces RPA3^+^ bridges during mitosis. Representative video of HeLa cells coexpressing mCh–RPA3 (magenta) and CHMP1B-L–GFP (cyan). Cells were incubated with 80 nM ICRF-193 and labelled with Hoechst 33342 (yellow). Images were taken every 15 s and the time is shown. White arrows indicate bridges. This video is related to Fig 1d.

Supplementary Video 2 CHMP1B encapsulates transfected DNA. Representative video of HeLa cells expressing CHMP1B-L–GFP (cyan) following transfection of pCR3.1 plasmid stained with SPY555-DNA (magenta). Frames were taken every 2 min across five *z* stacks with 1-µm steps. The time is shown. This video is related to Fig. 3a.

Supplementary Video 3 ICRF-193 induces CHMP4C bridges during mitosis. HeLa cells expressing CHMP4C-L–GFP (cyan) were labeled with SPY555-DNA (yellow) and treated with DMSO (top) or 80 nM ICRF-193 (bottom) for 1 h before imaging. Representative videos from each condition were combined. Images were taken every minute. Bridges are indicated with a white arrowhead. This video is related to Fig. 6b.

Supplementary Video 4 CHMP1B and CHMP7 are required to prevent binucleation of cells treated with ICRF-193. HeLa cells expressing YFP–LAP2B (green) were transfected with siNT, siCHMP7 and siCHMP1B and treated with 80 nM ICRF-193 for 1 h or transfected with siSMC2 and treated with DMSO. Cells were imaged every 10 min for 48 h. Time is indicated, with *t* = 0 min representing the point at which the YFP–LAP2B bridge formed. This video is related to Fig. 6g, top.

Supplementary Table 3 and 4 Supplementary Table 3: Statistical analysis. Supplementary Table 4: Western blot quantifications.

Light Microscopy Table Light microscopy table.

## Source data

Source Data Figs. 6 and 7 and Extended Data Figs. 1, 8 and 9 Unprocessed western blots.

Source Data Figs. 1, 2 and 5–7 and Extended Data Figs. 1–4, 8 and 9 Source data for all graphs.

## Data Availability

Further information and requests for resources and reagents should be directed to and will be fulfilled by the lead contact, J.M.-S. (juan.martin_serrano@kcl.ac.uk). Plasmids generated in this study were deposited to Addgene (Supplementary Information) or are available upon request from the lead contact J.M.-S. (juan.martin_serrano@kcl.ac.uk). Cryo-EM maps and models were deposited to the Electron Microscopy Data Bank (EMDB) and Protein Data Bank (PDB), respectively, with accession codes as follows: cellular CHMP1B–IST1 filaments class Ia (EMD-42922), class Ib (EMD-42923), class Ic (EMD-42925), class Id (EMD-42926), Class II (EMD-42927), Class III (EMD-42928) and Class IV (EMD-42929); recombinant CHMP1B–IST1 nucleic-acid-bound filaments dsDNA (EMD-42932, PDB 8V2S), ssDNA (EMD-42931, PDB 8V2R) and ssRNA (EMD-42930, PDB 8V2Q); focused nucleic acid classification Class 1 (EMD-42933), Class 2 (EMD-42934), Class 4 (EMD-42935), Class 5 (EMD-42936), and Class 9 (EMD-42937). Any additional information required to reanalyze the data reported in this paper is available from the lead contact upon request. Source data are provided with this paper.
