# Supplementary material for: ESCRT-III assembles around mis-segregated DNA to protect genome stability
Source: Nat Struct Mol Biol. Author manuscript; Available in PMC 2026 Aug 30. (PMC13468122; doi:10.1038/s41594-026-01841-4)
Supplement: Supplementary file 1 [file NIHMS2196638-supplement-Supplement.pdf]

nature structural & molecular biology

Supplementary information

https://doi.org/10.1038/s41594-026-01841-4

ESCRT-III assembles around mis-segregated DNA to protect genome stability

In the format provided by the authors and unedited

Supplementary Figure S1

a - IST1

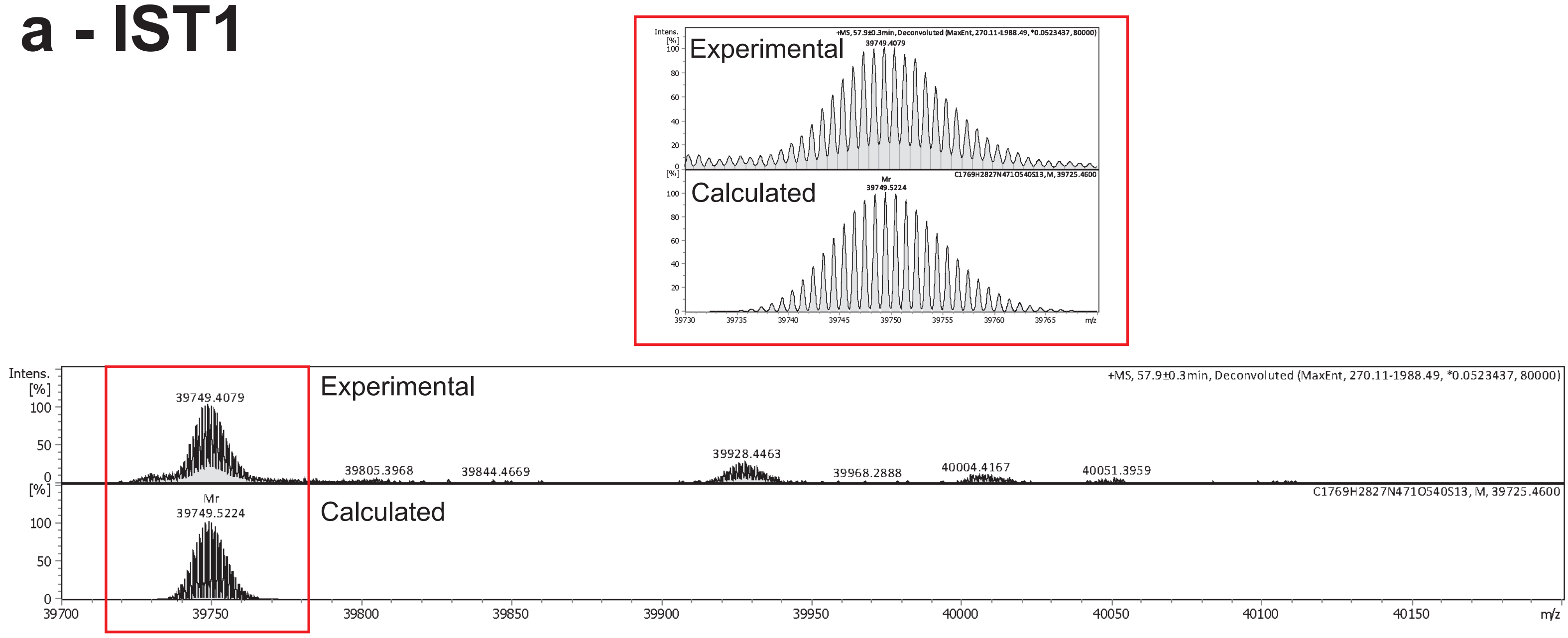

b - CHMP1B M136V

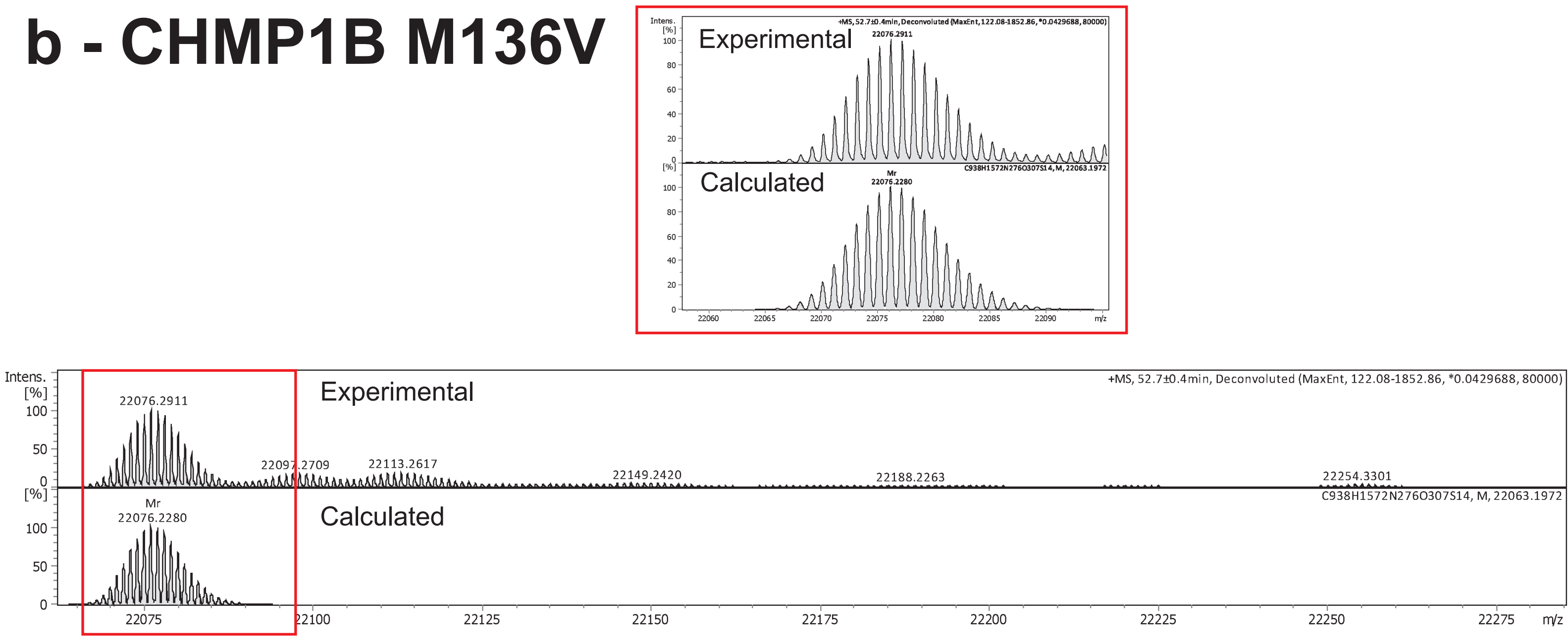

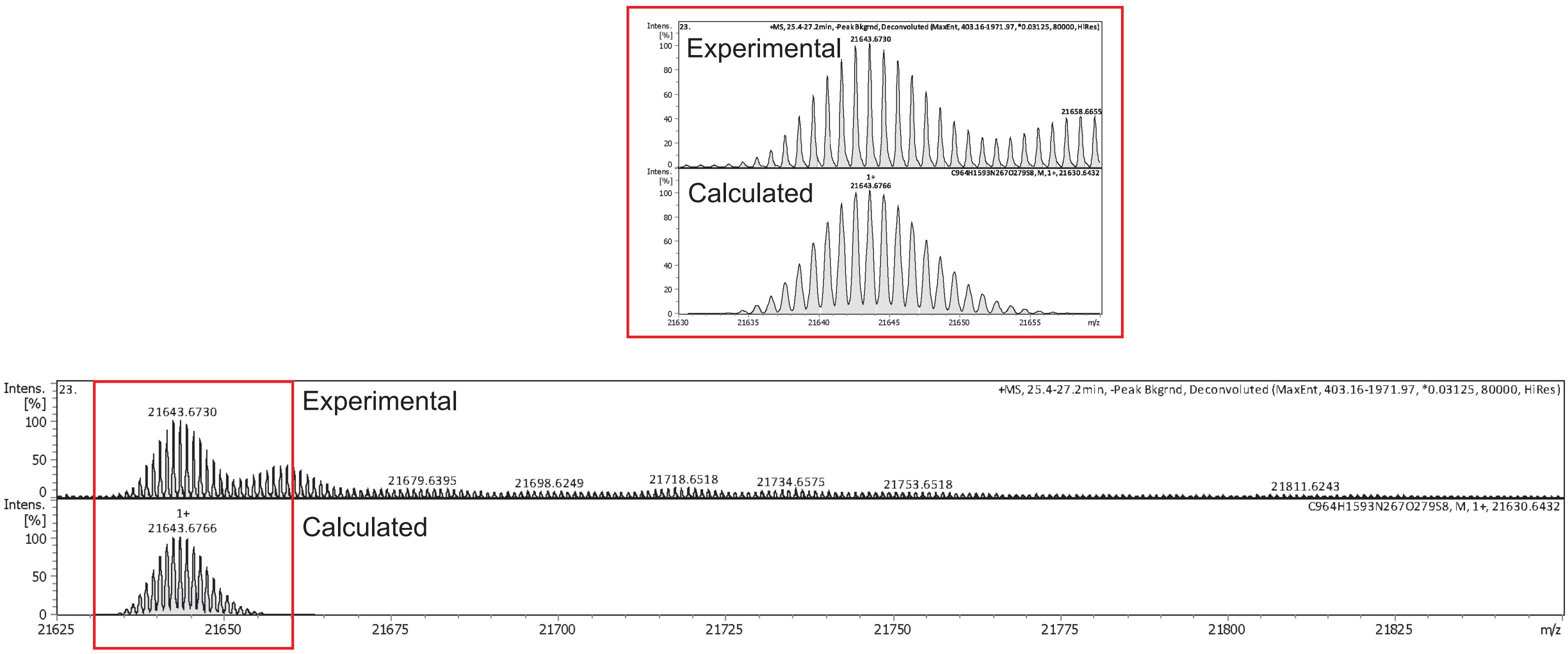

Supplementary Figure S1.
Electrospray spectra showing the whole mass measurements that demonstrate the expected identities of the three new recombinant proteins used in our studies: IST1 (**a**), CHMP1B M136V (**b**), and IST1NTD Y165A (**c**). In each case the experimental (above) and calculated spectra (below) are shown, together with expansions (boxes) showing the charge state ensembles. In all cases, the deconvoluted experimental masses were within 0.2 Da of the expected values, confirming the protein identities.

Supplementary Table 1: Cryo-EM data collection statistics for *in cellulo* over expressed CHMP1B/IST1 filaments.

	Cellular CHMP1B/IST1 filaments – Class II (EMDB-42932)	Cellular CHMP1B/IST1 filaments – Class III (EMDB-42931)	Cellular CHMP1B/IST1 filaments – Class IV (EMDB-42930)	Cellular CHMP1B/IST1 filaments – ClassIa (EMDB-42922)	Cellular CHMP1B/IST1 filaments – ClassIb (EMDB-42923)	Cellular CHMP1B/IST1 filaments – ClassIc (EMDB-42925)	Cellular CHMP1B/IST1 filaments – ClassId (EMDB-42926)
**Data collection and processing**							
Magnification	81,000	81,000	81,000	81,000	81,000	81,000	81,000
Voltage (kV)	300	300	300	300	300	300	300
Electron exposure (e–/Å^2)	46	46	46	46	46	46	46
Defocus range (μm)	-0.8 to -2.2	-0.8 to -2.2	-0.8 to -2.2	-0.8 to -2.2	-0.8 to -2.2	-0.8 to -2.2	-0.8 to -2.2
Pixel size (Å)	0.529	0.529	0.529	0.529	0.529	0.529	0.529
Symmetry imposed	C1, Helical	C1, Helical	C1, Helical	C1, Helical	C1, Helical	C1, Helical	C1, Helical
Initial particle images (no.)	70,300	70,300	70,300	70,300	70,300	70,300	70,300
Final particle images (no.)	3,070	3,065	2,005	21,059	18,235	4,418	9,125
Map resolution (Å)	4.20	4.37	4.35	3.53	3.50	5.78	4.27
FSC threshold	0.143	0.143	0.143	0.143	0.143	0.143	0.143
Map resolution range (Å)	1.31 to 44.89	1.26 to 46.20	1.27 to 48.60	2.60 to 54.31	1.02 to 35.72	1.67 to 60.64	1.23 to 39.01

Supplementary Table 2: Cryo-EM data collection, refinement and validation statistics for *in vitro* CHMP1B/IST1 nucleic acid complexes.

	CHMP1B/IST1 dsDNA bound copolymer (EMDB-42932) (PDB 8V2S)	CHMP1B/IST1 ssDNA bound copolymer (EMDB-42931) (PDB 8V2R)	CHMP1B/IST1 ssRNA bound copolymer (EMDB-42930) (PDB 8V2Q)
**Data collection and processing**			
Magnification	130,000	130,000	130,000
Voltage (kV)	200	200	200
Electron exposure (e–/Å^2)	50	50	50
Defocus range (μm)	-0.1 to -3.9	-0.1 to -3.4	-0.1 to -4.0
Pixel size (Å)	0.8936	0.8936	0.8936
Symmetry imposed	C1, Helical	C1, Helical	C1, Helical
Initial particle images (no.)	511,337	591,872	1,233,372
Final particle images (no.)	541,289	259,006	261,684
Map resolution (Å)	2.72	3.01	2.95
FSC threshold	0.143	0.143	0.143
Map resolution range (Å)	1.95 to 8.80	2.71 to 10.12	1.93 to 7.48
**Refinement**			
Initial model used (PDB code)	6TZ4, 3FRR	6TZ4, 3FRR	6TZ4, 3FRR
Model resolution (Å)	2.87	3.20	3.04
FSC threshold	0.5	0.5	0.5
Model resolution range (Å)	2.87	3.20	3.04
Map sharpening *B* factor (Å^2)	89.2	105	93.6
Model composition			
Non-hydrogen atoms	2916	2916	2916
Protein residues	368	368	368
Ligands	0	0	0
B factors (Å^2)			
Protein	89.2	89.2	89.2
Ligand	N/A	N/A	N/A
R.m.s. deviations			
Bond lengths (Å)	0.008	0.006	0.007
Bond angles (°)	1.586	1.400	1.502
Validation			
MolProbity score	0.86	0.76	0.86
Clashscore	1.18	0.34	1.34
Poor rotamers (%)	0.31	0.00	0.00
Ramachandran plot			
Favored (%)	98.34	99.17	98.90
Allowed (%)	1.66	0.83	1.10
Disallowed (%)	0.00	0.00	0.00

Supplementary Table 5: CryoEM data collection parameters

	In cellulo CHMP1B-IST1 filaments		Nucleic acid-bound CHMP1B-IST1 filaments			
**Sample**	***Dataset 1***	***Dataset 2***	**dsDNA**	**ssDNA**	**ssRNA**	**Nucleosome-bound CHMP1B-IST1 filaments**
Microscope	Titan Krios G3	Titan Krios G3	Glacios	Glacios	Glacios	Titan Krios G4
Voltage (keV)	300	300	200	200	200	300
Detector & Filter	Post-GIF K3 energy filter	Post-GIF K3 energy filter	Post-GIF Selectris X energy filter, Falcon4i	Post-GIF Selectris X energy filter, Falcon4i	Post-GIF Selectris X energy filter, Falcon4i	Post-GIF Selectris X energy filter, Falcon4i
Slit width (eV)	10	10	10	10	10	10
Acquisition Software	Serial EM	EPU	EPU	EPU	EPU	EPU
C2 Aperture (μm)	50	50	50	50	50	50
Nominal Magnification	81,000x	81,000x	130,000x	130,000x	130,000x	130,000x
Pixel Size (Å)	0.53(super-resolution)	1.06 (super-resolution)	0.8936	0.8936	0.8936	0.9286
Defocus Range (μm)	-0.8 to -2.2	-0.5 to -2.2	-0.8 to -1.8	-0.8 to -1.8	-0.8 to -1.8	-0.2 to -1.8
Total Dose ($e^-/Å^2$)	46	46	50	50	50	45
Frames / Fractions	50	50	50	50	50	50
Total Movies	1,901	1,359	12,598	3,272	6.020	6,111

Resources table

REAGENT or RESOURCE	SOURCE	IDENTIFIER
**Antibodies**		
Mouse anti-NUP153	Biolegend (1:500)	906201
Mouse anti-HSP90	Santa Cruz (1:10000)	sc-13119
Rabbit anti-CHMP4C	Lampire Biologicals (1:200)	Custom
Rabbit anti-NUP50	Ullman lab (1:500)	Custom
Rabbit anti-CHMP1B	Sundquist lab (1:500)	Custom
Mouse anti-Tubulin	Abcam (1:1000)	ab7291
Mouse anti-RPA2	Abcam (1:100)	ab2175
Rabbit anti-Emerin	Santa Cruz (1:300)	sc-25284
Rabbit anti-IST1	Sundquist lab (1:10000)	Custom
Chicken anti-GFP	Abcam (1:1000)	ab13970
Rabbit anti-Lamin B1	Abcam (1:1000)	ab16048
Rabbit anti-CHMP7	Proteintech (1:1000)	16424-1-AP
Rabbit anti-mCh	Abcam (1:1000)	ab167453
Mouse anti-BAF	Abcam (1:1000)	ab129184
Rabbit anti-ULK3	Abcam (1:1000)	ab124947
Mouse anti-CEP55	Proteintech (1:1000)	67460-1-Ig
Rabbit anti-MKLP1	Santa Cruz (1:1000)	sc-867
Rabbit anti-LEM2	Sigma-Aldrich (1:100)	HPA017340
Rabbit anti-53BP1	Novus Biologicals (1:750)	NB100-304
HRP-linked anti-mouse IgG	Cell Signalling Technology (1:1000)	7076S
HRP-linked anti-rabbit IgG	Cell Signalling Technology (1:1000)	7074S
Goat anti-mouse IgG (H+L) (DyLight 680 Conjugate)	Cell Signalling Technology (1:10000)	5470S
Goat anti-mouse IgG (H+L) (DyLight 800 Conjugate)	Cell Signalling Technology (1:10000)	5151S
Mouse anti-GFP	Abcam (1:1000)	ab1218
Goat anti-mouse IgG (H+L) Alexa Fluor 488	Thermofisher (1:2000)	A-11001
Goat anti-rabbit IgG (H+L) Alexa Fluor 568	Themofisher (1:2000)	A-11011

Mouse anti-GFP	Roche (1:1000)	11814460001
Rabbit anti-CHMP4B	Proteintech (1:1000)	13683-1-AP
Goat anti-chicken IgY (H+L) Alexa Fluor 488	Thermofisher (1:1000)	A-11039
Goat anti-mouse IgG (H+L) Alexa Fluor 555	Thermofisher (1:1000)	A-21424
Goat anti-mouse IgM (H) Alexa Fluor 488	Thermofisher (1:1000)	A-21042
Donkey anti-rabbit IgG (H+L) Alexa Fluor 555	Thermofisher (1:1000)	A-31572
Goat anti-rouse IgG (H+L) Alexa Fluor 647	Thermofisher (1:1000)	A-21244
**Chemicals and peptides**		
**Chemicals**		
Lipofectamine RNAiMAX	Thermofisher	13778075
Polyethylenimine (PEI)	Polysciences	23966-1
Hoechst 33258	Sigma-Aldrich	861405
SiR-tubulin kit	Spirochrome	SC002
SPY555-DNA	Spirochrome	SC201
SPY595-DNA	Spirochrome	SC301
Click-iT EdU Imaging Kit, Alexa Fluor 555 dye	Thermofisher	C10338
ICRF-193	Sigma-Aldrich	14659
PBS	Gibco	10010-015
DMEM	Gibco	41966-029
TrypLE Express	Gibco	12605-010
Dimethyl Sulfoxide (DMSO)	Sigma-Aldrich	41639
Polybrene	Merck	TR-1003-G
PBS, pH 7.2	Gibco	12549079
PFA	Electron Microscopy Sciences	15710-S
BSA	Sigma-Aldrich	A9647-100G
Triton X-100	Sigma-Aldrich	T9284-500ML
Laemmli Sample Buffer	Bio-Rad	1610747
Uranyl acetate	Electron Microscopy Sciences	22400
Osmium Tetroxide	Electron Microscopy Sciences	19110

Embed 812 Embedding Kit	Electron Microscopy Sciences	14121
Paraformaldehyde 3% Glutaraldehyde 0.35% In 0.1M Sodium Cacodylate Buffer pH7.4	Electron Microscopy Sciences	15949
Acetone	Electron Microscopy Sciences	10015
**Peptides**		
IST1 316-366:<br>GCPADNYDNFVLPELPSVPDTLPTA SAGASTSASEDIDFDDLSRRFEELKK KT	This paper	Calculated Mass (Da) 6196<br>Measured Mass (Da) 6192
CHMP1A 182-196:<br>CSQEDQLSRRLAALRN	This paper	Calculated Mass (Da) 2324<br>Measured Mass (Da) 2323
CHMP1A 140-196:<br>GCTTPQEQVDSLIMQIAEENGLEVL DQLSQLPEGASAVGESSVRSQEDQ LSRRLAALRN	This paper	Calculated Mass (Da) 6369<br>Measured Mass (Da) 6365
CHMP1B 169-199:<br>CPQGQTGSVGTSVASAEQDELSQR LARLRDQV	This paper	Calculated Mass (Da) 3852<br>Measured Mass (Da) 3850
CHMP1B 183-199: CAEQDELSQRLARLRDQV	This paper	Calculated Mass (Da) 2594<br>Measured Mass (Da) 2593
CHMP2A 152-222: GCGDEEDEEESDAVVSQVLDELGL SLTDELSNLPSTGGSLSVAAGGKKA EAAASALADADADLEERLKNLRRD	This paper	Calculated Mass (Da) 7939<br>Measured Mass (Da) 7939
CHMP2B 141-213:<br>GCMINDTLDDIFDGSDDEEESQDIVN QVLDEIGIEISGKMAKAPSAARSLPS ASTSKATISDEEIERQLKALGVD	This paper	Calculated Mass (Da) 8448<br>Measured Mass (Da) 8446
CHMP2B 196-213:<br>CTISDEEIERQLKALGVD	This paper	Calculated Mass (Da) 2483<br>Measured Mass (Da) 2482
CHMP3 159-222:<br>GCEEAEMEIDRILFEITAGALGKAPS KVTDALPEPEPPGAMAASEDEEEEE EALEAMQSRLATLRS	This paper	Calculated Mass (Da) 7525<br>Measured Mass (Da) 7525
CHMP4A 153-222:<br>GCGDDVDEDELLEELEELEQEELAQ ELLNVGDKEEEPSVKLPSVPSTHLP AGPAPKVDEDEEALKQLAEWVS	This paper	Calculated Mass (Da) 8375<br>Measured Mass (Da) 8372
CHMP4B 156-224:	This paper	Calculated Mass (Da) 8424

GCGEEFDEDELMAELEELEQEELDK NLLEISGPETVPLPNVPSIALPSKPAK KKEEEDDDMKELENWAGSM		Measured Mass (Da) 8423
CHMP4C 156-233: GCGDDFDEDELMAELEELEQEELNK KMTNIRLPNVPSSSLPAQPNRKPGM SSTARRSRAASSQRAEEEDDDIKQL AAWAT	This paper	Calculated Mass (Da) 8907 Measured Mass (Da) 8904
CHMP5 148-219: GCALSRSYGTPELDEDDLEAELDAL GDELLADEDSSYLDEAASAPAIPEG VPTDTKNKDGVLVDEFGLPQIPAS	This paper	Calculated Mass (Da) 8187 Measured Mass (Da) 8186
CHMP6 145-201: GCAGSFTQEDEDAILEELSAITQEQI ELPEVPSEPLPEKIPENVPVKARPRQ AELVAAS	This paper	Calculated Mass (Da) 6835 Measured Mass (Da) 6835
CHMP7 366-453: GCAGGVTNGLDFDSEELEKELDILL QDTTKEPLDLPDNPRNRHFTNSVPN PRISDAELEAELEKLSLSEGGLVPSS KSPKRQLEPTLKPL	This paper	Calculated Mass (Da) 10315 Measured Mass (Da) 10314
**Deposited data**		
Cellular CHMP1B-IST1 Copolymer, Protein only, Class Ia	This paper	EMD-42922
Cellular CHMP1B-IST1 Copolymer, Protein only, Class Ib	This paper	EMD-42923
Cellular CHMP1B-IST1 Copolymer, Protein only, Class Ic	This paper	EMD-42925
Cellular CHMP1B-IST1 Copolymer, Protein only, Class Id	This paper	EMD-42926
Cellular CHMP1B-IST1 Copolymer, Nucleic acid bound, Class II	This paper	EMD-42927
Cellular CHMP1B-IST1 Copolymer, Nucleic acid bound, Class III	This paper	EMD-42928
Cellular CHMP1B-IST1 Copolymer, Nucleic acid bound, Class IV	This paper	EMD-42929
Recombinant CHMP1B-IST1 Copolymer, dsDNA bound	This paper	EMD-42932 PDB:8V2S
Recombinant CHMP1B-IST1 Copolymer, dsDNA bound focused refinement, Class1	This paper	EMD-42933
Recombinant CHMP1B-IST1 Copolymer, dsDNA bound focused refinement, Class2	This paper	EMD-42934

Recombinant CHMP1B-IST1 Copolymer, dsDNA bound focused refinement, Class4	This paper	EMD-42935
Recombinant CHMP1B-IST1 Copolymer, dsDNA bound focused refinement, Class5	This paper	EMD-42936
Recombinant CHMP1B-IST1 Copolymer, dsDNA bound focused refinement, Class9	This paper	EMD-42937
Recombinant CHMP1B-IST1 Copolymer, sDNA bound	This paper	EMD-42931 PDB: 8V2R
Recombinant CHMP1B-IST1 Copolymer, ssRNA bound	This paper	EMD-42930 PDB: 8V2Q
**Experimental models: Cell lines**		
HeLa	ATCC	CRM-CCL-2
293T	ATCC	CRL-3216
**Oligonucleotides**		
CHMP7 siRNA 5'-GGGAGAAGAUUGUGAAGUUUUUU-3'	Dharmacon	Custom
ON-TARGET plus siControl non-targeting pool	Dharmacon	D-001810-10-20
CHMP4C siRNA 5-'CTCACTCAGATTGATGGCACA-3'	Qiagen	SI04279674
NUP153 siRNA 5'-GGACUUGUUAGAUCUAGUU-3'	Dharmacon	Custom
NUP50 siRNA 5'-GGAGGACGCUUUUCUGGAUTT-3'	Dharmacon	Custom
CHMP1B siRNA 5'-UGGACAAAUUCGAGCACCAUUUU-3'	Dharmacon	Custom
BAF siRNA 5'-GGCCUAUGUUGUCCUUGGC-3'	Ambion	Custom
ULK3 siRNA 5'-GCAAGGCUCUGGACUUCUU-3'	Dharmacon	D-004949-01-0050
CEP55 siRNA 5'-GGAGAAGAAUGCUUAUCAAAUU-3'	Dharmacon	Custom
dsDNA random 60mer (base pairs) 5'- AGATTTGAGGTAAACCAA TTGAGCACATAGTCGCGCTAT CCGACAATCTCCAAATTATAA -3'	IDT	Custom
ssDNA random 60mer (bases)	IDT	Custom

5'- AGATTTGAGGTAAACCAA TTGAGCACATAGTCGCGCTAT CCGACAATCTCCAAATTATAA -3'		
ssRNA random 60mer (bases)<br>5'- rArGrArUrUrUrGrArGrGrUrA rArArCrCrArArUrUrGrArGrC rArCrArUrArGrUrCrGrCrGrC rUrArUrCrCrGrArCrArArUrC rUrCrCrArArArUrUrArUrArA -3'	IDT	Custom
**Recombinant DNA**		
pET28a(+) 6xHIS-SUMO-CHMP1B	Genscript Inc.	Addgene: 212115
pET28c(+) 6xHIS-SUMO-IST1	Genecript Inc.	Addgene: 212114
pCMS28 mCh-RPA3	This paper	
pNG72 CHMP1B-L-GFP	This paper	
pMGF196 LEM2-mCh	Addgene	#97005
pNG72 GFP	https://doi.org/10.1073/pnas.1805504115	
pNG72 CHMP4C-L-GFP	https://doi.org/10.1016/j.isci.2020.101244	
pNG72 YFP-LAP2B (residues 244-454)	DOI: 10.1126/science.1217180	
MLV Gag-pol	Paul Bieniasz	
pHIT-VSVG	Paul Bieniasz	
pNG72 CHMP4B-L-GFP	https://doi.org/10.1016/j.devcel.2018.11.012	
pCAG-Myc-IST1-FL	This paper	Internal: WISP 07-77<br>DNASU: HsCD00751713<br>Uniprot: P53390-4
pCAG-CHMP1B-FOS	This paper	Internal: WISP 23-01<br>Addgene: 211825<br>Uniprot: Q7LBR1
pGEX IST1 1-189	This paper	Internal: WISP 07-74<br>DNASU: HsCD00520979<br>Uniprot: P53390-4
pGEX IST1 1-189 Y165A	This paper	Internal: WISP 09-55<br>DNASU: HsCD00520956<br>Uniprot: P53390-4
pCA528 CHMP1A 140-196	This paper	Internal: WISP 18-01<br>Addgene: 104593

		Uniprot: Q9HD42
pCA528 CHMP2A 152-222	This paper	Internal: WISP 18-03<br>Addgene: 104594<br>Uniprot: O43633
pCA528 CHMP2B 141-213	This paper	Internal: WISP 18-04<br>Addgene: 104595<br>Uniprot: Q9UQN3
pCA528 CHMP3 159-222	This paper	Internal: WISP 18-05<br>Addgene: 104599<br>Uniprot: Q9Y3E7
pCA528 CHMP4A 153-222	This paper	Internal: WISP 18-06<br>Addgene: 108256<br>Uniprot: Q9BY43
pCA528 CHMP4B 156-224	This paper	Internal: WISP 18-07<br>Addgene: 104596<br>Uniprot: Q9H444
pCA528 CHMP4C 156-233	This paper	Internal: WISP 18-09<br>Addgene: 108257<br>Uniprot: Q96CF2
pCA528 CHMP5 148-219	This paper	Internal: WISP 18-10<br>Addgene: 108259<br>Uniprot: Q9NZZ3
pCA528 CHMP6 145-201	This paper	Internal: WISP 18-11<br>Addgene: 104597<br>Uniprot: Q96FZ7
pCA528 CHMP7 366-453	This paper	Internal: WISP 18-12<br>Addgene: 104598<br>Uniprot: 104598
pCA528 IST1 316-366	This paper	Internal: WISP 14-121<br>DNASU: HSCD00696258<br>Uniprot: P53390-4
**Software**		
CryoSPARC	https://cryosparc.com/	V42.1 and V4.3.1
RELION	https://github.com/3dem/relion	v4.0-beta-2-commit-2fba16
ISOLDE		V1.6.0

ImageJ (Fiji)	https://fiji.sc, https://doi.org/10.1038/nmeth.2019	V2.9.0/1.53t
Prism	https://www.graphpad.com	V10.1.0
NIS Elements AR	https://www.microscope.healthcare.nikon.com/	V4.13.05
Servalcat	https://github.com/keitaroyam/servalcat	V0.4.39
Molprobity	https://github.com/rlabduke/MolProbity	V4.5.2
