# Supplementary material for: ESCRT-III assembles around mis-segregated DNA to protect genome stability
Source: Nat Struct Mol Biol. 2026 Jul 15;33(8):1236–51. doi: 10.1038/s41594-026-01841-4 (PMC13468122; doi:10.1038/s41594-026-01841-4)
Supplement: Supplementary file 1 [file 41594_2026_1841_MOESM2_ESM.pdf]

nature portfolio

Corresponding author(s): Adam Frost, Wes Sundquist and Juan Martin-Serrano

Last updated by author(s): 26/03/26

Reporting Summary

Nature Portfolio wishes to improve the reproducibility of the work that we publish. This form provides structure for consistency and transparency in reporting. For further information on Nature Portfolio policies, see our Editorial Policies and the Editorial Policy Checklist.

Statistics

For all statistical analyses, confirm that the following items are present in the figure legend, table legend, main text, or Methods section.

n/a	Confirmed	
☐	☒	The exact sample size (*n*) for each experimental group/condition, given as a discrete number and unit of measurement
☐	☒	A statement on whether measurements were taken from distinct samples or whether the same sample was measured repeatedly
☐	☒	The statistical test(s) used AND whether they are one- or two-sided<br>*Only common tests should be described solely by name; describe more complex techniques in the Methods section.*
☒	☐	A description of all covariates tested
☒	☐	A description of any assumptions or corrections, such as tests of normality and adjustment for multiple comparisons
☐	☒	A full description of the statistical parameters including central tendency (e.g. means) or other basic estimates (e.g. regression coefficient) AND variation (e.g. standard deviation) or associated estimates of uncertainty (e.g. confidence intervals)
☐	☒	For null hypothesis testing, the test statistic (e.g. *F*, *t*, *r*) with confidence intervals, effect sizes, degrees of freedom and *P* value noted<br>*Give P values as exact values whenever suitable.*
☒	☐	For Bayesian analysis, information on the choice of priors and Markov chain Monte Carlo settings
☒	☐	For hierarchical and complex designs, identification of the appropriate level for tests and full reporting of outcomes
☒	☐	Estimates of effect sizes (e.g. Cohen's *d*, Pearson's *r*), indicating how they were calculated

Our web collection on statistics for biologists contains articles on many of the points above.

Software and code

Policy information about availability of computer code

Data collection	NIS Elements AR V4.13.05 was used to collect images on Nikon microscopes.<br>Thermo Scientific EPU V3.4 and SerialEM V4.1.0 beta were used the collect Cryo-EM data.
Data analysis	ImageJ (Fiji) V2.9.0/1.53t was used to display and analyze images.<br>Prism V10.1.0 was used to display quantifications and perform statistical tests.<br>Cryo-EM data processing and structure building used the following software: RELION-v4.0-beta-2-commit-2fba16, CryoSPARC-v4.3.1 (and v4.2.1), ChimeraX-1.6.1, ISOLDE-v1.6, and COOT-v0.9.8.1.

For manuscripts utilizing custom algorithms or software that are central to the research but not yet described in published literature, software must be made available to editors and reviewers. We strongly encourage code deposition in a community repository (e.g. GitHub). See the Nature Portfolio guidelines for submitting code & software for further information.

Data

Policy information about availability of data

All manuscripts must include a data availability statement. This statement should provide the following information, where applicable:

- Accession codes, unique identifiers, or web links for publicly available datasets
- A description of any restrictions on data availability
- For clinical datasets or third party data, please ensure that the statement adheres to our policy

Source data has been provided. All other data supporting the findings of this study are available on request from the corresponding author.

Research involving human participants, their data, or biological material

Policy information about studies with human participants or human data. See also policy information about sex, gender (identity/presentation), and sexual orientation and race, ethnicity and racism.

Reporting on sex and gender	No human participants or human data
Reporting on race, ethnicity, or other socially relevant groupings	No human participants or human data
Population characteristics	No human participants or human data
Recruitment	No human participants or human data
Ethics oversight	No human participants or human data

Note that full information on the approval of the study protocol must also be provided in the manuscript.

Field-specific reporting

Please select the one below that is the best fit for your research. If you are not sure, read the appropriate sections before making your selection.

[x] Life sciences   [ ] Behavioural & social sciences   [ ] Ecological, evolutionary & environmental sciences

For a reference copy of the document with all sections, see nature.com/documents/nr-reporting-summary-flat.pdf

Life sciences study design

All studies must disclose on these points even when the disclosure is negative.

Sample size	No sample-size calculations were performed. Details of sample size are found in their respective figure legends. We chose sample sizes based on previous experiments which would sufficiently detect meaningful differences.
Data exclusions	No data were excluded.
Replication	All data presented are representative of at least three biological replicates.
Randomization	Cells from the same culture plate were seeded into separate wells of a plate, treatments and transfections were performed in parallel and suitable controls were used.
Blinding	Investigators were not blinded during acquisition or analysis as this was not feasible.

Reporting for specific materials, systems and methods

We require information from authors about some types of materials, experimental systems and methods used in many studies. Here, indicate whether each material, system or method listed is relevant to your study. If you are not sure if a list item applies to your research, read the appropriate section before selecting a response.

Materials & experimental systems

n/a	Involved in the study
☐	☒ Antibodies
☐	☒ Eukaryotic cell lines
☒	☐ Palaeontology and archaeology
☐	☒ Animals and other organisms
☒	☐ Clinical data
☒	☐ Dual use research of concern
☒	☐ Plants

Methods

n/a	Involved in the study
☒	☐ ChIP-seq
☒	☐ Flow cytometry
☒	☐ MRI-based neuroimaging

Antibodies

Antibodies used

Antibody Source (Dilution) Identifier
Mouse anti-NUP153 Biolegend (1:500) 906201
Mouse anti-HSP90 Santa Cruz (1:10000) sc-13119
Rabbit anti-CHMP4C Lampire Biologicals (1:200) Custom
Rabbit anti-NUP50 Ullman lab (1:500) Custom
Rabbit anti-CHMP1B Sundquist lab (1:500) Custom
Mouse anti-Tubulin Abcam (1:1000) ab7291
Mouse anti-RPA2 Abcam (1:100) ab2175
Rabbit anti-Emerin Santa Cruz (1:300) sc-25284
Rabbit anti-IST1 Sundquist lab (1:10000) Custom
Chicken anti-GFP Abcam (1:1000) ab13970
Rabbit anti-Lamin B1 Abcam (1:1000) ab16048
Rabbit anti-CHMP7 Proteintech (1:1000) 16424-1-AP
Rabbit anti-mCherry Abcam (1:1000) ab167453
Mouse anti-BAF Abcam (1:1000) ab129184
Rabbit anti-ULK3 Abcam (1:1000) ab124947
Mouse anti-CEP55 Proteintech (1:1000) 67460-1-Ig
Rabbit anti-MKLP1 Santa Cruz (1:1000) sc-867
Rabbit anti-LEMD2 Sigma-Aldrich (1:100) HPA017340
Rabbit anti-53BP1 Novus Biologicals (1:750) NB100-304
HRP-linked anti-mouse IgG Cell Signalling Technology (1:1000) 7076S
HRP-linked anti-rabbit IgG Cell Signalling Technology (1:1000) 7074S
Goat anti-mouse IgG (H+L) (DyLight 680 Conjugate) Cell Signalling Technology (1:10000) 5470S
Goat anti-mouse IgG (H+L) (DyLight 800 Conjugate) Cell Signalling Technology (1:10000) 5151S
Mouse anti-GFP Abcam (1:1000) ab1218
Goat anti-mouse IgG (H+L) Alexa Fluor 488 Thermofisher (1:2000) A-11001
Goat anti-rabbit IgG (H+L) Alexa Fluor 568 Themofisher (1:2000) A-11011
Mouse anti-GFP Roche (1:1000) 11814460001
Rabbit anti-CHMP4B Proteintech (1:1000) 13683-1-AP
Goat anti-chicken IgY (H+L) Alexa Fluor 488 Thermofisher (1:1000) A-11039
Goat anti-mouse IgG (H+L) Alexa Fluor 555 Thermofisher (1:1000) A-21424
Goat anti-mouse IgM (H) Alexa Fluor 488 Thermofisher (1:1000) A-21042
Donkey anti-rabbit IgG (H+L) Alexa Fluor 555 Thermofisher (1:1000) A-31572
Goat anti-rouse IgG (H+L) Alexa Fluor 647 Thermofisher (1:1000) A-21244

Validation

All commercial primary antibodies were validated by the manufacturer (source) for detection of human protein. Primary antibodies from the Sundquist and Ullman labs were validated separately for the detection of human protein. Rabbit anti-IST1 was validated in PMCID: PMC2649257, rabbit anti-CHMP1B was validated in PMCID: PMC2919903 andrabbit anti-NUP50 was validated in PMCID: PMC2995170.

Eukaryotic cell lines

Policy information about cell lines and Sex and Gender in Research

Cell line source(s)

Hela (CRM-CCL-2), 293T (CRL-3216) and hTERT RPE1 (CRL-4000) were purchased from ATCC.

Authentication

Authentication was performed by ATCC and we also occasionally test for authenticity by direct sequencing (a University of Utah core service).

Mycoplasma contamination

Cell lines were regularly tested for the presence of mycoplasma using MycoAlert (Lonza) or or Mycoplasma PCR Detection Kit (Applied Biological Materials)

Commonly misidentified lines
(See ICLAC register)

No commonly misidentified lines were used in this study.

Animals and other research organisms

Policy information about studies involving animals; ARRIVE guidelines recommended for reporting animal research, and Sex and Gender in Research

Laboratory animals	No laboratory animals involved
Wild animals	No wild animals involved
Reporting on sex	n/a
Field-collected samples	No samples collected from the field
Ethics oversight	No ethicals approval or guidance was required

Note that full information on the approval of the study protocol must also be provided in the manuscript.

Plants

Seed stocks	No seed stocks used
Novel plant genotypes	n/a
Authentication	n/a
