# Supplementary material for: ESCRT-III assembles around mis-segregated DNA to protect genome stability
Source: Nat Struct Mol Biol. 2026 Jul 15;33(8):1236–51. doi: 10.1038/s41594-026-01841-4 (PMC13468122; doi:10.1038/s41594-026-01841-4)

Source Data Figs. 4, 5, 6, 7, ED Figs. 1, 8 & 9

Related to Fig. 4b

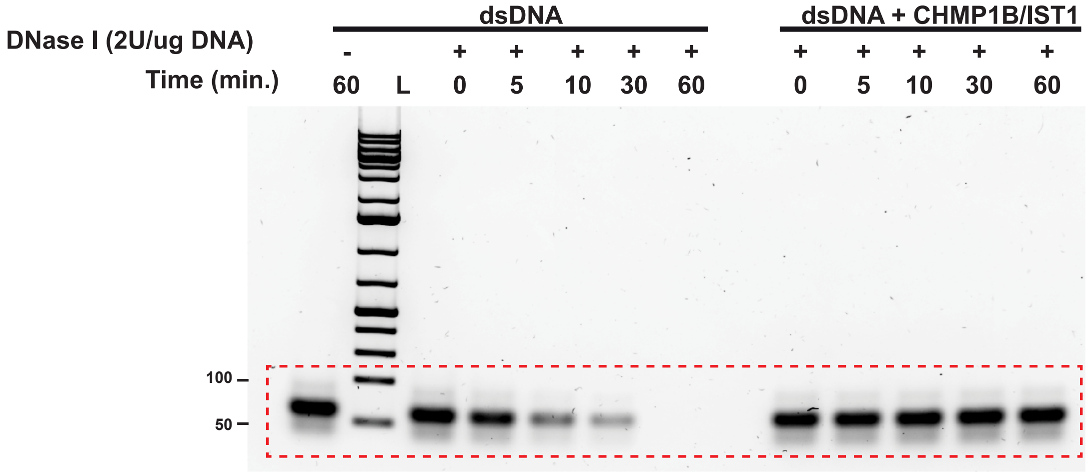

Related to Fig. 5b

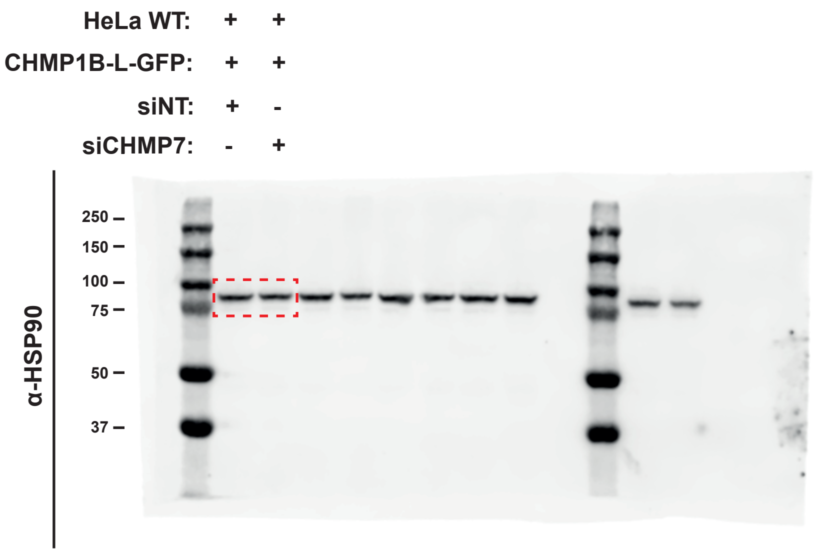

Relative density: 1 0.91

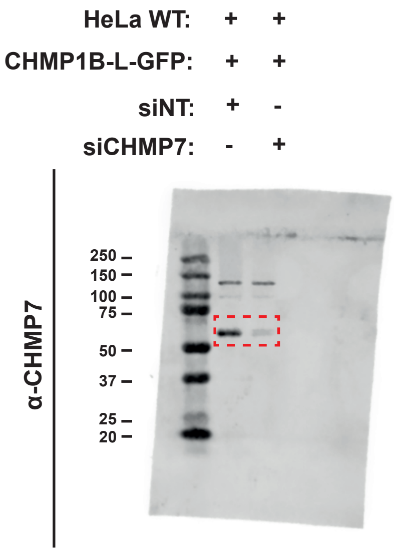

Relative density: 1 0.14
(Adjusted to HSP90)

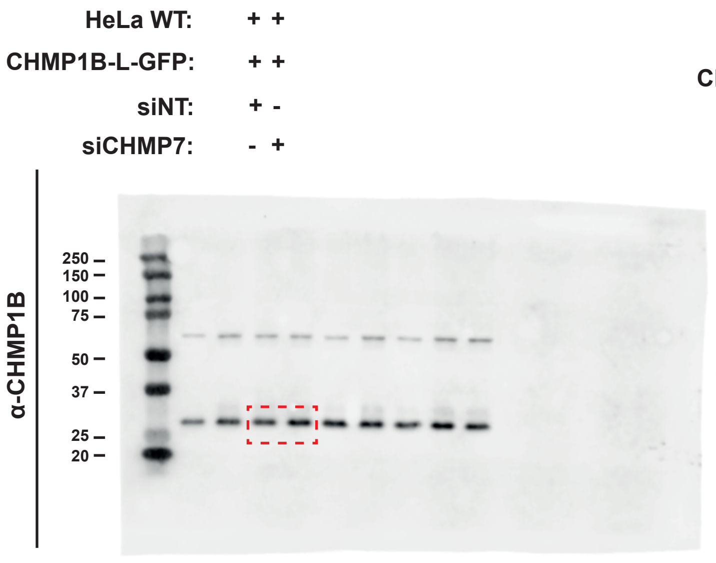

Related to Fig. 5c

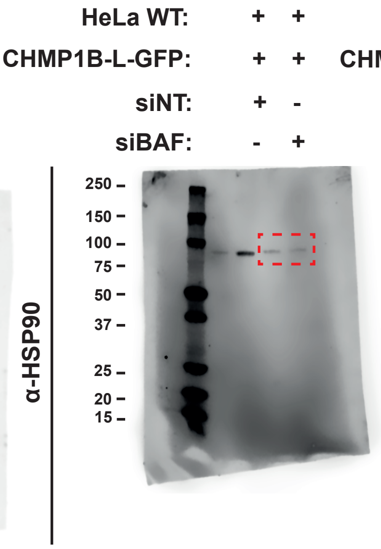

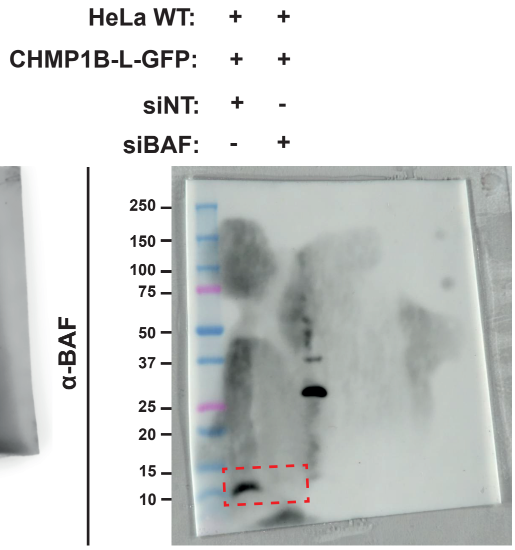

Relative density: 1 0.90 Relative density: 1 0.00
(Adjusted to HSP90)

Related to Fig. 6c

HeLa WT: + +
CHMP1B-L-GFP: + +
siNT: + -
siCHMP4C: - +

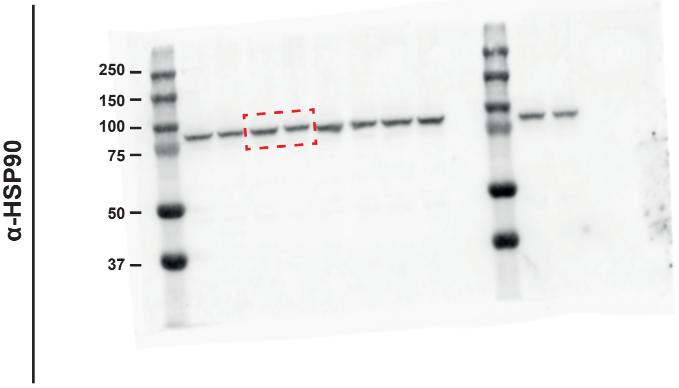

Relative density: 1 0.76

HeLa WT: + +
CHMP1B-L-GFP: + +
siNT: + -
siCHMP4C: - +

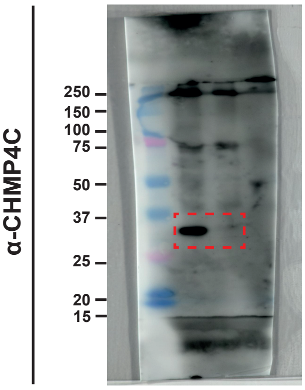

Relative density: 1 0.00
(Adjusted to HSP90)

Related to Fig. 6d

HeLa WT: + +
CHMP1B-L-GFP: + +
siNT: + -
siULK3: - +

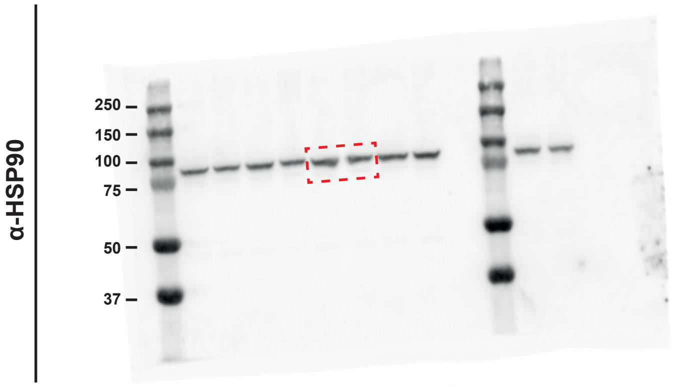

Relative density: 1 0.80

HeLa WT: + +
CHMP1B-L-GFP: + +
siNT: + -
siULK3: - +

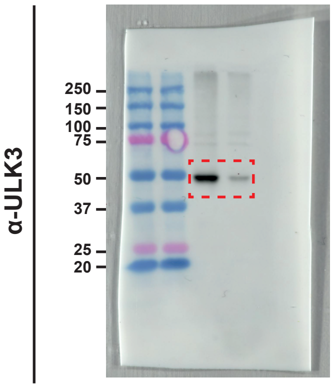

Relative density: 1 0.01
(Adjusted to HSP90)

Related to Fig. 6e

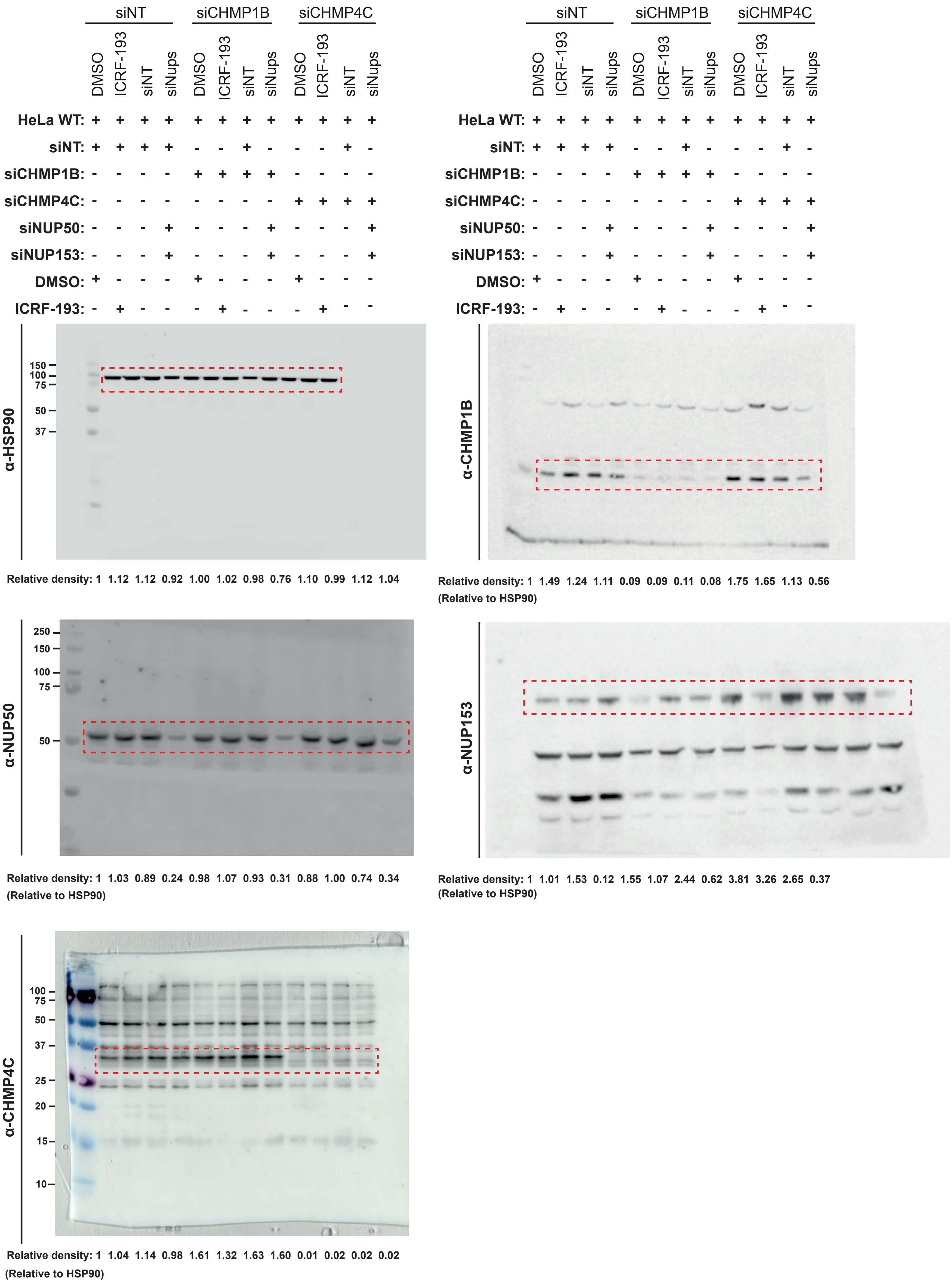

Related to Fig. 6f

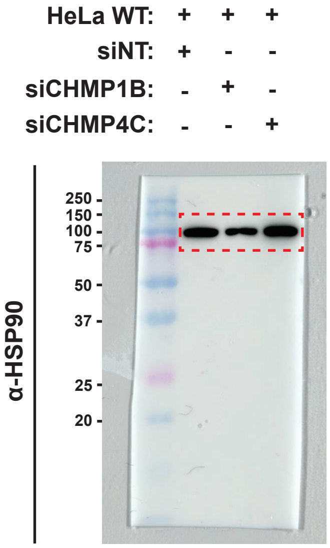

Relative density: 1 0.75 1.04

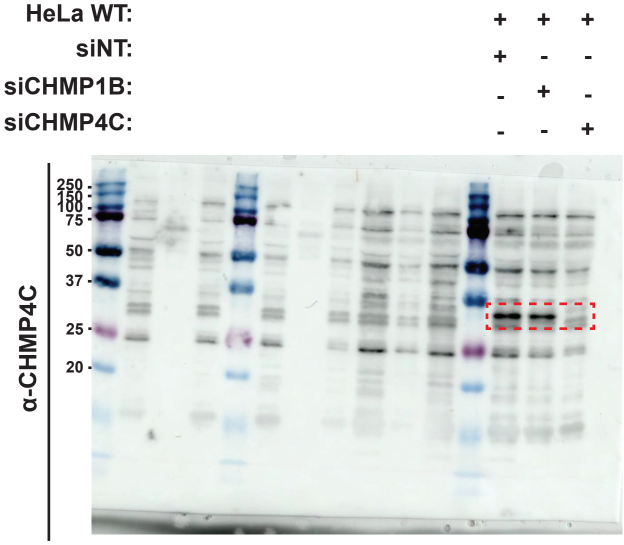

Relative density: 1 1.49 0.02
(Adjusted to HSP90)

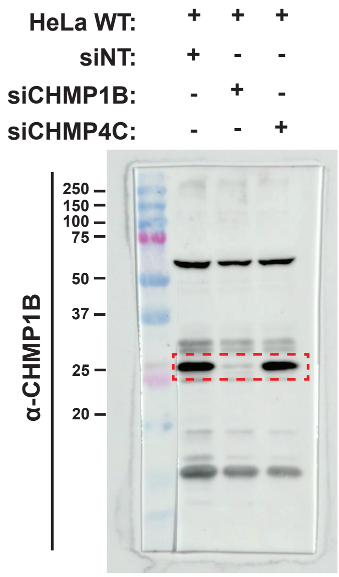

Relative density: 1 0.03 0.92
(Adjusted to HSP90)

Related to Fig. 6g

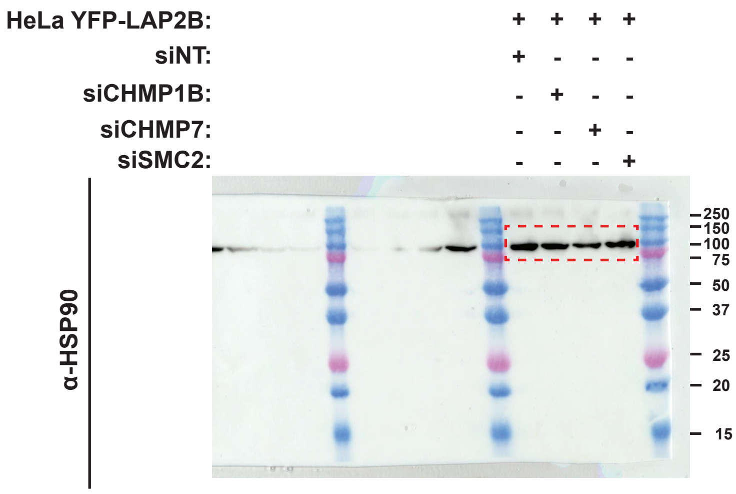

Relative density: 1 0.86 0.83 1.13

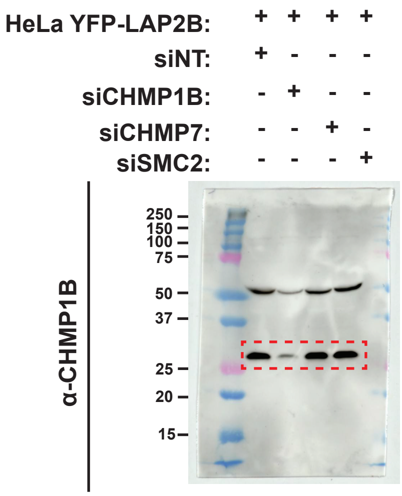

Relative density: 1 0.18 1.26 1.03
(Adjusted to HSP90)

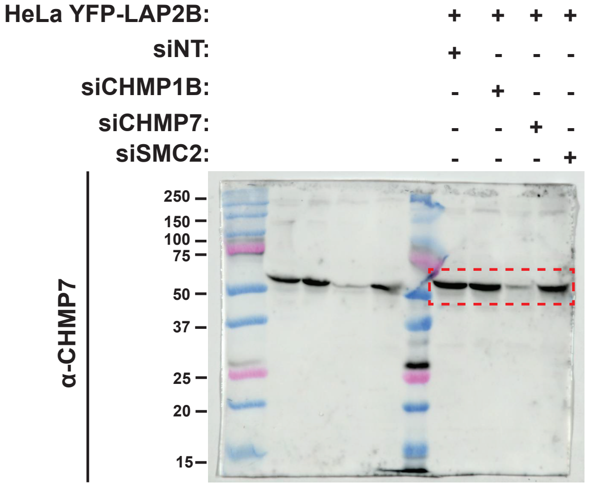

Relative density: 1 1.33 0.16 1.07
(Adjusted to HSP90)

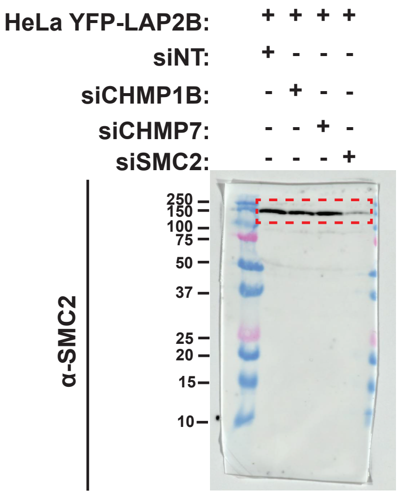

Relative density: 1 1.08 1.14 0.16
(Adjusted to HSP90)

Related to Fig. 7b

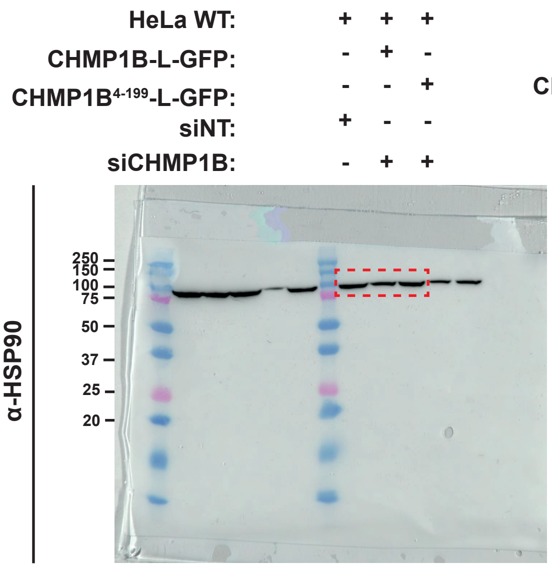

Relative density: 1 0.76 0.91

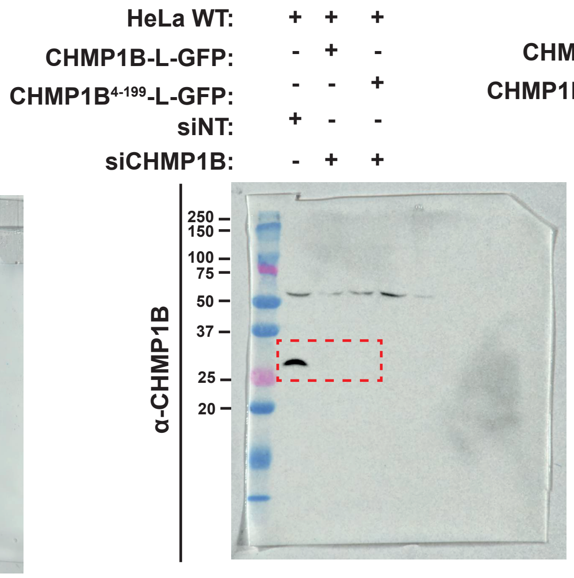

Relative density: 1 0.00 0.00
(Adjusted to HSP90)

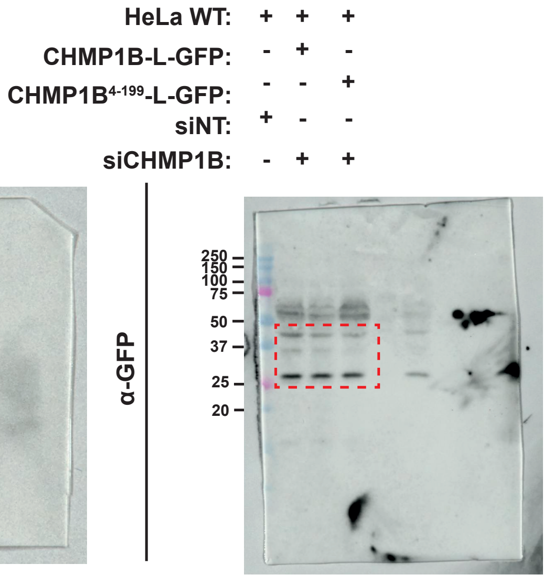

Related to Fig. 7f

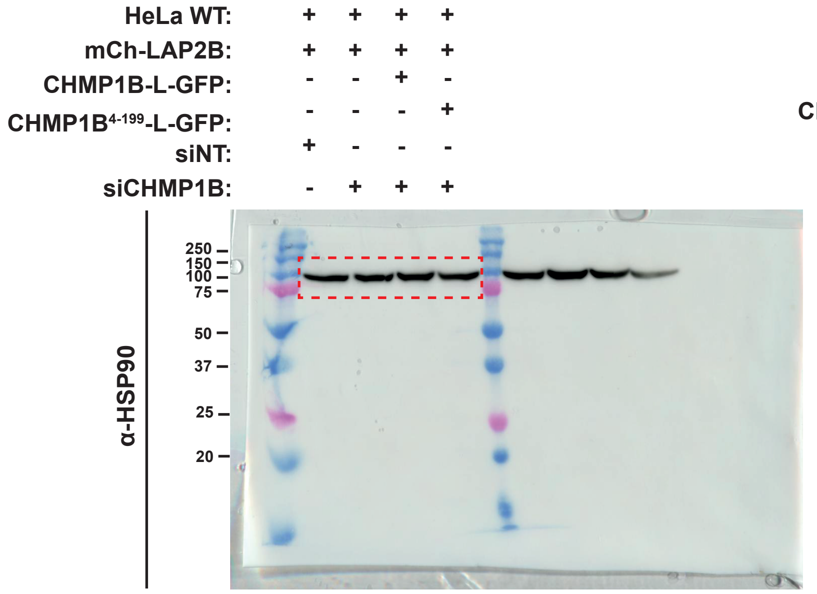

Relative density: 1.00 1.04 1.16 1.02

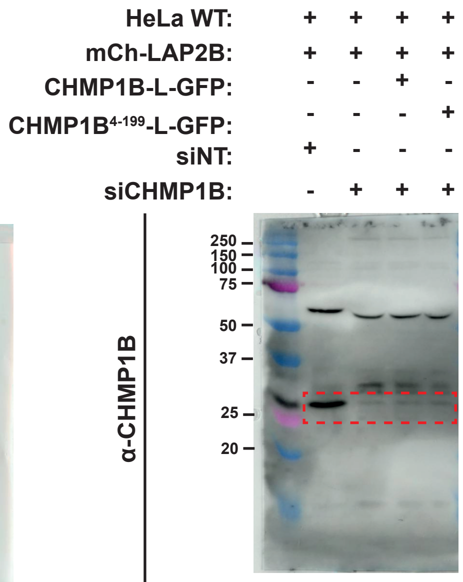

Relative density: 1.00 0.07 0.00 0.02
(Adjusted to HSP90)

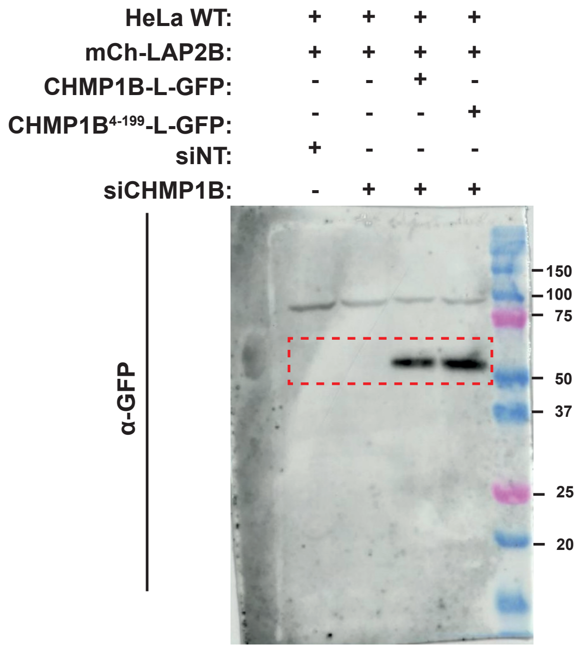

Related to Extended Data Fig. 1b

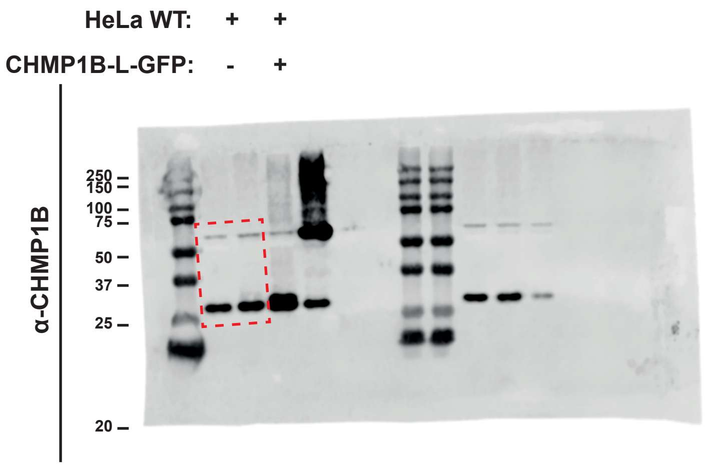

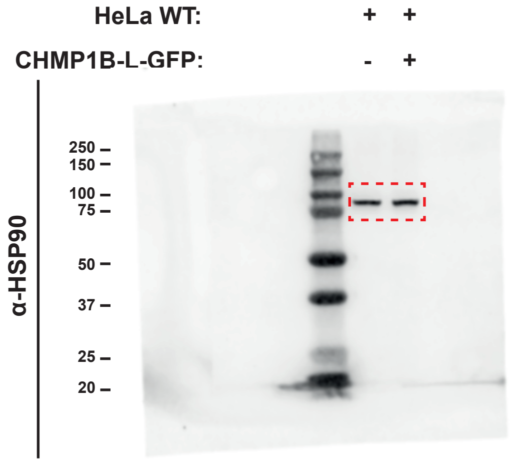

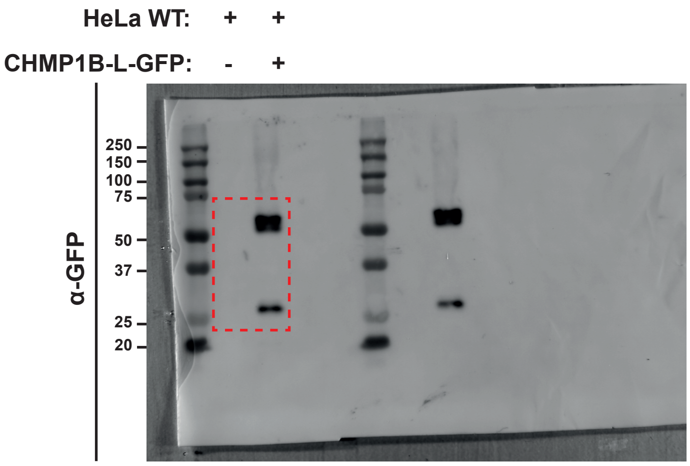

Related to Extended Data Fig. 1d

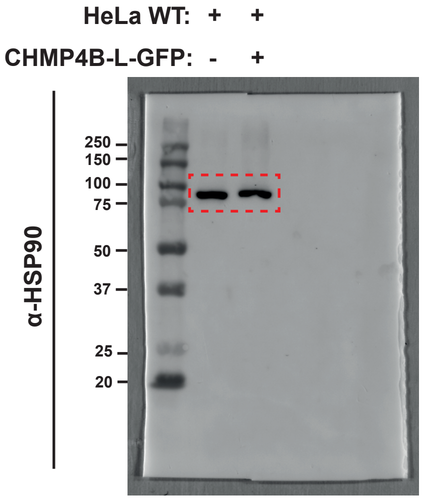

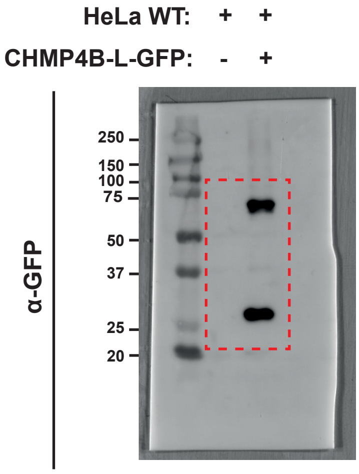

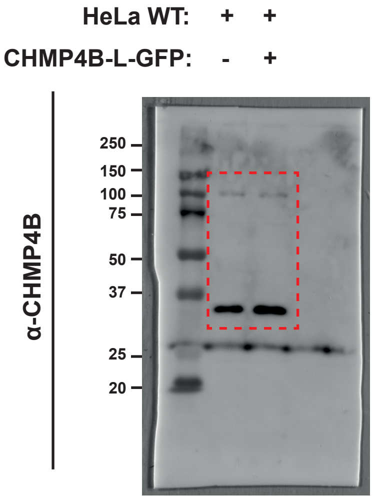

Related to Extended Data Fig. 1f

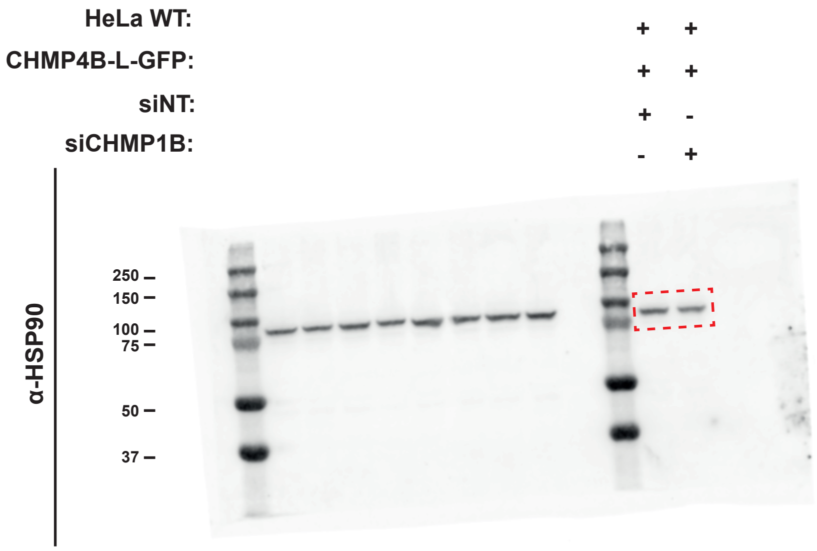

Relative density: 1 0.83

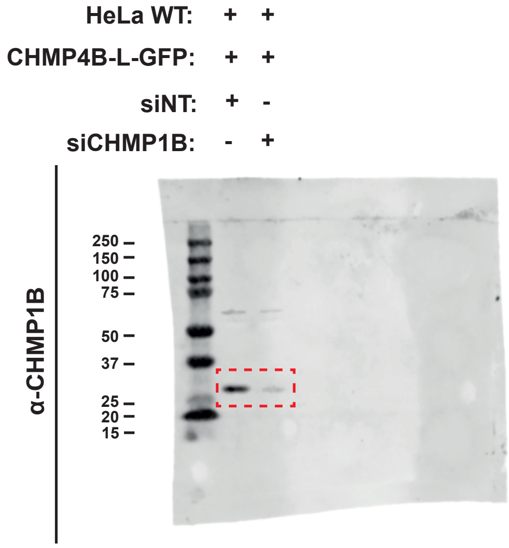

Relative density: 1 0.12
(Adjusted to HSP90)

Related to Extended Data Fig. 8a

HeLa WT: + +
CHMP1B-L-GFP: + +
siNT: + -
siCEP55: - +

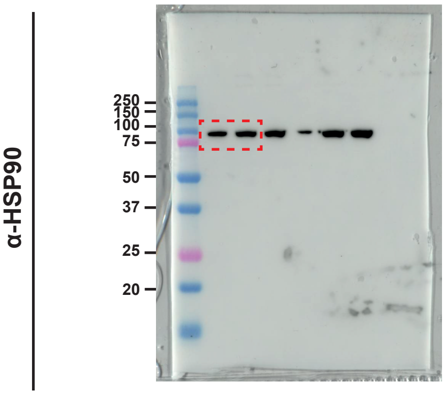

Relative density: 1 1.31

HeLa WT: + +
CHMP1B-L-GFP: + +
siNT: + -
siCEP55: - +

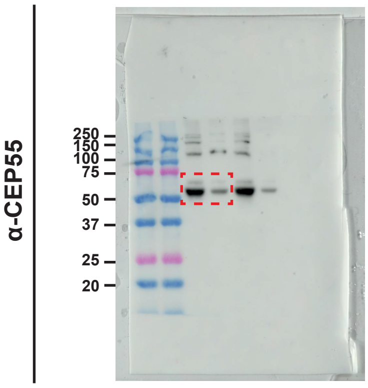

Relative density: 1 0.17
(Adjusted to HSP90)

Related to Extended Data Fig. 9a

HeLa WT: + +
CHMP4C-L-GFP: - +

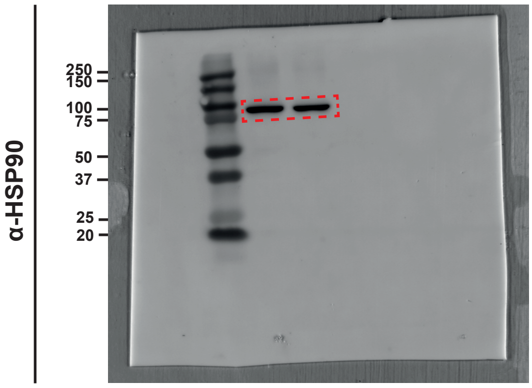

HeLa WT: + +
CHMP4C-L-GFP: - +

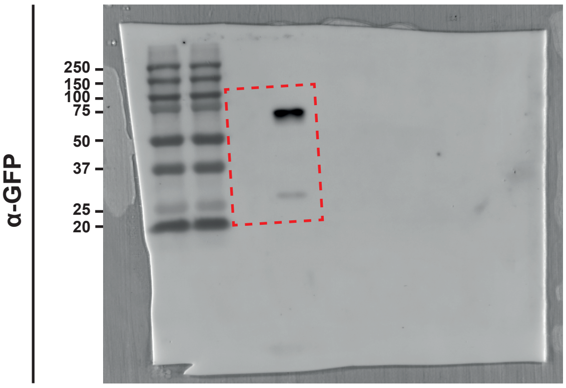

Related to Extended Data Fig. 9e

HeLa WT: + + + +
CHMP1B-L-GFP: - - + +
siNT: + - + -
siCHMP1B: - + - +

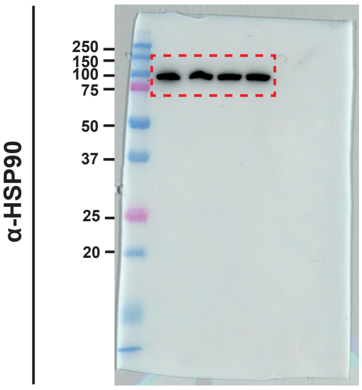

Relative density: 1 0.93 0.98 1.07

HeLa WT: + + + +
CHMP1B-L-GFP: - - + +
siNT: + - + -
siCHMP1B: - + - +

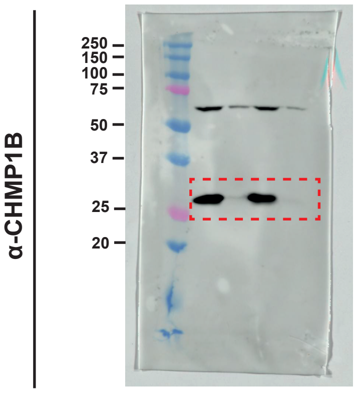

Relative density: 1 0.06 1.25 0.06
(Adjusted to HSP90)

HeLa WT: + + + +
CHMP1B-L-GFP: - - + +
siNT: + - + -
siCHMP1B: - + - +

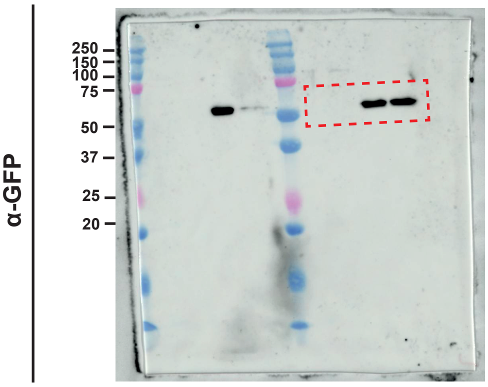

Supplement: Supplementary file 2 [file 41594_2026_1841_MOESM9_ESM.pdf]
